# *Xanthomonas campestris* sensor kinase HpaS co‐opts the orphan response regulator VemR to form a branched two‐component system that regulates motility

**DOI:** 10.1111/mpp.12901

**Published:** 2020-01-09

**Authors:** Rui‐Fang Li, Xin‐Xin Wang, Liu Wu, Li Huang, Qi‐Jian Qin, Jia‐Li Yao, Guang‐Tao Lu, Ji‐Liang Tang

**Affiliations:** ^1^ State Key Laboratory for Conservation and Utilization of Subtropical Agro‐bioresources College of Life Science and Technology Guangxi University Nanning China; ^2^ Guangxi Key Laboratory of Biology for Crop Diseases and Insect Pests Plant Protection Research Institute Guangxi Academy of Agricultural Sciences Nanning China

**Keywords:** branched two‐component system, motility, orphan response regulator, sensor kinase, *Xanthomonas*

## Abstract

*Xanthomonas campestris* pv. *campestris* (Xcc) controls virulence and plant infection mechanisms via the activity of the sensor kinase and response regulator pair HpaS/hypersensitive response and pathogenicity G (HrpG). Detailed analysis of the regulatory role of HpaS has suggested the occurrence of further regulators besides HrpG. Here we used in vitro and in vivo approaches to identify the orphan response regulator VemR as another partner of HpaS and to characterize relevant interactions between components of this signalling system. Bacterial two‐hybrid and protein pull‐down assays revealed that HpaS physically interacts with VemR. Phos‐tag SDS‐PAGE analysis showed that mutation in *hpaS* reduced markedly the phosphorylation of VemR in vivo. Mutation analysis reveals that HpaS and VemR contribute to the regulation of motility and this relationship appears to be epistatic. Additionally, we show that VemR control of Xcc motility is due in part to its ability to interact and bind to the flagellum rotor protein FliM. Taken together, the findings describe the unrecognized regulatory role of sensor kinase HpaS and orphan response regulator VemR in the control of motility in Xcc and contribute to the understanding of the complex regulatory mechanisms used by Xcc during plant infection.

## Graphical abstract

Sensor kinase HpaS regulates T3SS and swimming motility by controlling the phosphorylation of response regulators HrpG and VemR, respectively.

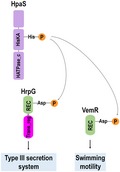

## 1 INTRODUCTION

Two‐component systems (TCSs) are common mechanisms that bacteria use to sense and respond to environmental stimuli. A typical TCS consists of two proteins: a membrane‐associated histidine sensor kinase that perceives changes in the environment and a cytoplasmic response regulator that enables the cell to alter its behaviour accordingly (Stock *et al*., 2000; Buschiazzo and Trajtenberg, 2019). In general, on sensing an appropriate environmental stimulus, the sensor kinase protein autophosphorylates at a highly conserved histidine residue in the transmitter domain. Subsequently, the phosphate group is transferred to an aspartic acid residue in the N‐terminal receiver domain of the cognate response regulator, resulting in a conformational change and the activation of its C‐terminal output domain, which frequently has DNA‐binding capacity. As a result, these types of TCSs play an essential role in responding to changes in the environment through phenotype modification via alteration of gene expression (Dziejman and Mekalanos, 1995; Stock *et al*., 2000).

In the majority of cases, the genes encoding proteins that make up the TCS components are often co‐transcribed as an operon in the genome. However, there are now several examples of orphan sensor kinases and response regulators described in the literature, for example EpsW in *Myxococcus xanthus* (Black *et al*., 2015) and RocA in *Streptococcus* (Lynskey *et al*., 2019). Nevertheless, due to the lack of obvious cognate partners, these are less well characterized. TCSs have been described as showing a high level of specificity, with sensor kinases controlling only their cognate response regulators (Laub and Goulian, 2007), although continued research into TCSs has also revealed a phenomenon of cross talk, in which a non‐cognate sensor kinase phosphorylates a response regulator in the absence of the cognate kinase (Siryaporn and Goulian, 2008; Guckes *et al*., 2013; Agrawal *et al*., 2015). Moreover, an increasing number of TCS architectures with two or more interacting sensor kinases or response regulators have also been described (for review, see Buelow and Raivio, 2010; Francis and Porter, 2019). The phosphorylation pathway in these TCSs is branched, with more than one source or target of phosphotransfer. These branched pathways present a “many‐to‐one” structure, in which multiple sensor kinases phosphorylate a single response regulator, or a “one‐to‐many” structure, in which one sensor kinase phosphorylates multiple response regulators (for a review, see Laub and Goulian, 2007).

Several TCSs with a “one‐to‐many” structure have been reported. Arguably, the most well‐studied example is the sensor kinase CheA that phosphorylates both response regulators CheY and CheB, which control the chemotaxis system of *Escherichia coli* (Szurmant and Ordal, 2004). A variation of this system is found in *Rhodobacter sphaeroides*, where sensor kinase CheA_2_, which is a protein with the same domain structure as CheA from *E. coli*, can phosphorylate all five chemotaxis response regulators, CheB_1_, CheB_2_, CheY_3_, CheY_4_ and CheY_6_, and the CheA_3_ (Scott *et al*., 2010). These examples demonstrate the level of complexity in these TCSs in bacteria.

*Xanthomonas campestris* pv. *campestris* (Xcc) is the causal agent of black rot diseases of cruciferous crops worldwide and is an important model for studying bacterial plant infection (Vicente and Holub, 2013). This pathogen encodes a number of virulence factors, such as type III secretion system (T3SS)‐dependent effectors (Büttner and Bonas, 2002), cyclic glucans (Rigano *et al*., 2007), lipopolysaccharides (Braun *et al*., 2005), extracellular polysaccharide (EPS, also called xanthan gum) (Katzen *et al*., 1998), and a series of extracellular enzymes including amylase, endoglucanase, polygalacturonate lyase, and protease (Dow and Daniels, 1994). These virulence factors can suppress, interfere with, or induce the innate immunity responses of host plants. Xcc contains an extensive array of genes encoding TCSs, a feature that probably reflects the adaptability this organism needs during its life cycle. Recent analysis has revealed that an Xcc genome generally encodes 32 histidine kinase sensors, 54 response regulators, and 20 histidine‐containing phosphotransfer (HPt) domain proteins (da Silva *et al*., 2002; Qian *et al*., 2005; Vorhölter *et al*., 2008). However, the functions and possible significance of many of these signalling proteins remains largely unexplored. Several TCSs have been defined to play important roles in regulating Xcc virulence and biofilm formation. The TCS RpfC/RpfG positively modulates the expression of extracellular enzymes and EPS, and negatively regulates the biosynthesis of a diffusible signal factor (*cis*‐11‐methyl‐2‐dodecenoic acid) that functions in cell‐to‐cell communication by controlling the *rpf* (regulation of pathogenicity factor) gene cluster (Tang *et al*., 1991; He *et al*., 2006; Ryan *et al*., 2007). ColS_XC1050_/ColR_XC1049_, which is a global regulatory system, regulates various cellular processes, including proliferation, virulence, hypersensitive response (HR), and stress tolerance (Qian *et al*., 2008; Zhang *et al*., 2008). RavS/RavR regulates EPS synthesis, biofilm production, and motility by altering cellular cyclic‐di‐guanosine monophosphate (GMP) levels and RavR (XC_2228), annotated as a cyclic‐di‐GMP phosphodiesterase A, is involved in cyclic‐di‐GMP hydrolysis (He *et al*., 2009).

The protein hypersensitive response and pathogenicity‐associated sensor (HpaS) is one of the most conserved sensor kinase proteins in Xcc genomes (Li *et al*., 2014) and plays a key role in the perception of environmental signals, contributing to virulence and the HR. Recent data suggest that HpaS and response regulator hypersensitive response and pathogenicity G (HrpG) form a TCS (Li *et al*., 2014). Mutation of *hpaS* or *hrpG* almost completely abolishes HR induction and virulence in Xcc (Li *et al*., 2014). In the same study it was demonstrated that HpaS physically interacts with and phosphorylates HrpG. Additionally, HrpG has been shown to modulate the expression of an AraC‐family transcription factor HrpX and a MarR‐family transcription factor HpaR, which contribute to the regulation of the T3SS in Xcc (Wengelnik *et al*., 1996; Wei *et al*., 2007). However, HrpG could not account for the full regulatory scope of HpaS or the additional phenotypes this sensor kinase appeared to regulate. This suggests that additional regulatory elements are under the control of HpaS.

In this work, we have established that the sensor kinase HpaS regulates distinct downstream functions in Xcc through a second pathway by co‐opting response regulator VemR (XC_2252). VemR is an orphan response regulator previously shown to be involved in controlling EPS production, cell motility, and virulence in Xcc (Tao and He, 2010). However, a cognate sensor kinase for VemR has not been identified to date. We reveal that HpaS is required for virulence, EPS production, motility, and stress tolerance. Additionally, we present genetic and biochemical evidence to showing that HpaS regulates swimming motility specifically through VemR. Taken together, the results reported here call attention to the importance of branched TCSs in Xcc and contribute to understanding of the regulatory mechanisms used by the sensor kinase HpaS via the orphan response regulator VemR to allow Xcc to adapt to changing environments.

## 2 RESULTS

### 2.1 HpaS influences the expression of genes involved in a series cellular process in Xcc

The full HpaS protein is 413 amino acids in length; it contains a signal peptide, two transmembrane regions, and HAMP, PAS, HisKA, and HATPase_c domains (Figure S1). Our previous work demonstrated that the HpaS controls a very select set of genes important for the HR and virulence of Xcc (Li *et al*., 2014). To gain broader insight into the regulatory role of HpaS in Xcc a transcriptome analysis was conducted. For this, Xcc wild‐type 8004 and the *hpaS* mutant strain (designated ∆*hpaS*) (Table S1) were grown to the mid‐exponential phase (OD_600_ = 0.6) in NYG medium (see Experimental procedures), a medium that has been widely used in studies of the morphology, biology, and preservation of Xcc (Daniels *et al*., 1984b). RNA samples were extracted from two independent biological replicates.

Following bacterial RNA extraction, library construction, and sequencing, the generated data were analysed to assess differential gene expression (see Experimental procedures). Of the 4,273 annotated protein‐coding genes of Xcc strain 8004, 547 genes were found to be differentially expressed by 2‐fold or more (Table S2). Of these, 337 were up‐regulated and 210 were down‐regulated. To corroborate the RNA‐Seq data, 25 differentially expressed genes (DEGs) were selected randomly for the validation by semiquantitative reverse‐transcription PCR (RT‐PCR). Expression of these selected genes was consistent with the data from the transcriptome analyses (Table S3).

**Figure 1:**
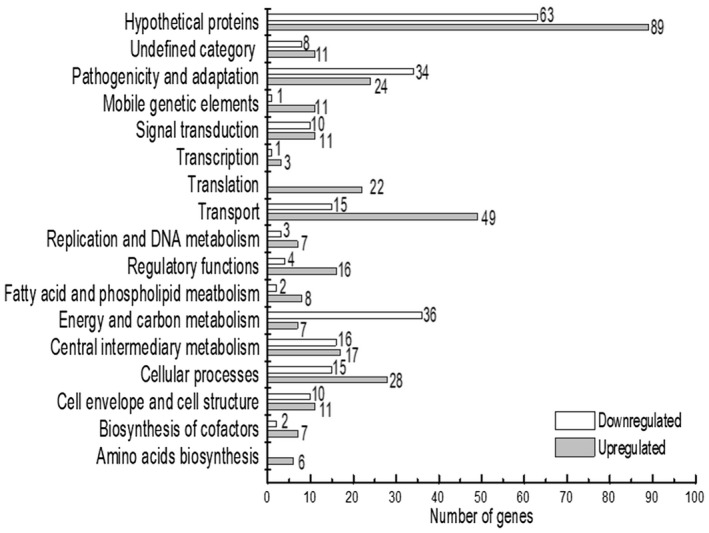
HpaS affects the expression of numerous genes in *Xanthomonas campestris* pv. *campestris* (Xcc). Functional categories of differential expressed genes in Δ*hpaS* mutant. Genome‐scale transcriptome profiling of Xcc strains cultured in NYG medium were investigated by RNA‐sequencing and 547 genes were found differentially expressed by 2‐fold or more in Δ*hpaS* mutant (Table S2). These genes were broadly categorized according to their biological function (He *et al*., 2007; Qian *et al*., 2005)

Functional clustering analysis, according to the annotation of the Xcc strain 8004 genome (Qian *et al*., 2005; He *et al*., 2007), was carried out where the majority of the 547 genes regulated by HpaS were assigned to functional categories that are based on cluster of genes (COG), including “transport”, “pathogenicity and adaption”, “cellular process”, “energy and carbon metabolism”, “central intermediary metabolism”, “translation”, “signal transduction”, “cell envelope and cell structure”, and “regulatory functions”. A total of 171 genes were predicted to encode hypothetical proteins or have not been given a functional category to date (Figure 1 and Table S2). The most dominant functional categories that genes were assigned to were “transport” and “pathogenicity and adaption” (Figure 1).

HpaS had a significant impact on genes that contribute to EPS, extracellular enzymes, motility, and stress tolerance (Figure 1 and Table S2). For example, *XC_1660*, *XC_1661*, *XC_1664*, *XC_1665*, *XC_1667*, *XC_1688*, and *XC_1689* or genes that encode the proteins involved in EPS synthesis were down‐regulated in the Δ*hpaS* mutant. *XC_0738*, *XC_0739*, *XC_0740*, and *XC_0744* that encode proteins involved in the type II secretion system, and *XC_3376* and *XC_3377* that encode extracellular proteases were up‐regulated in the Δ*hpaS* mutant. Notably, 21 chemotaxis‐associated genes (*XC_0286*, *XC_0336*, *XC_0638*, *XC_1412*, *XC_1413*, *XC_1414*, *XC_1801*, *XC_1802*, *XC_2136*, *XC_2223*, *XC_2233*, *XC_2300*, *XC_2303*, *XC_2304*, *XC_2308*, *XC_2309*, *XC_2318*, *XC_2320*, *XC_2321*, *XC_2504*, *XC_3724*) were found to be up‐regulated in the Δ*hpaS* mutant, and six flagellum‐related genes (*XC_2234*, *XC_2235*, *XC_2245*, *XC_2247*, *XC_2259*, *XC_2266*) involved in swimming motility were down‐regulated. Taken together, the findings suggest that under the conditions tested HpaS has a broader regulatory role than previously observed.

### 2.2 HpaS controls diverse cellular processes including EPS production, extracellular enzyme activity, cell motility, and tolerance to various stresses

HpaS has been shown to be important for HR and virulence of Xcc (Li *et al*., 2014). However, the data above suggest that this signalling protein has a greater regulatory role than originally envisioned. To further investigate whether HpaS contributes to the regulation of other cellular processes, we conducted a series of basic phenotypic tests to examine the influence that the mutation of *hpaS* might have on EPS production, extracellular enzymes (cellulose, amylase and protease) secretion, cell motility, and adaption to stress and antimicrobials (see Experimental procedures).

**Figure 2:**
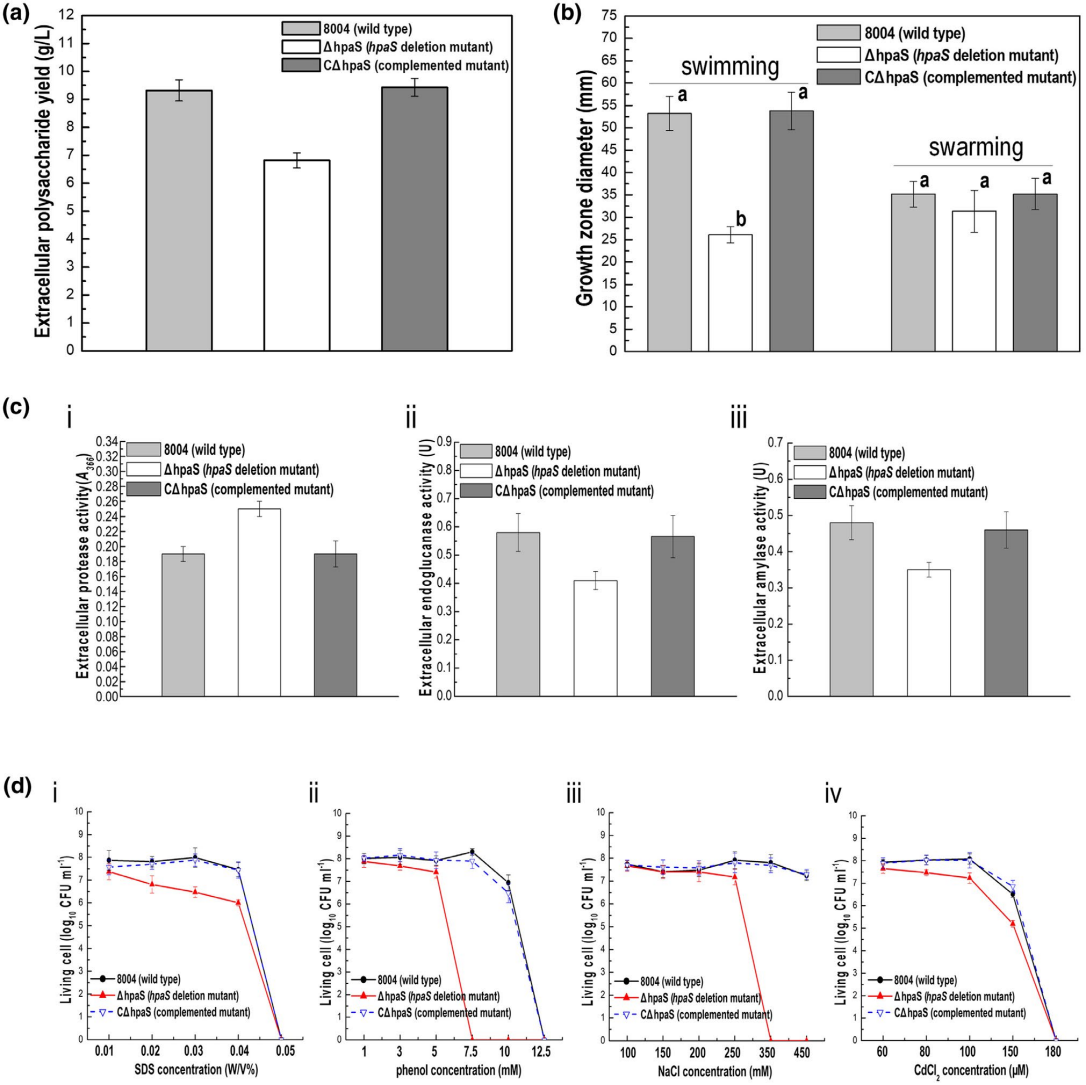
HpaS positively regulates extracellular polysaccharide (EPS) production, motility, and stress tolerances but negatively regulates extracellular protease in *Xanthomonas campestris* pv. *campestris* (Xcc). (a) EPS yield of tested Xcc strains. The wild type, Δ*hpaS* mutant, and the complemented strain CΔ*hpaS* were cultured in NY medium containing 2% glucose for 3 days before EPS was extracted and quantified. (b) Motility of tested Xcc strains. Xcc strains inoculated on “swim” (0.28% agar) medium plates and “swarm” (0.6% agar) medium plates for 4 and 3 days at 28 °C, respectively. Colony diameters of each strain were measured. Data are shown as means ± *SD*. (c) Mean relative quantity of extracellular enzymes produced by tested Xcc strains cultured in NYG medium for 24 hr. Protease (i), endoglucanase (ii), and amylase (iii). (d) Stress tolerances in tested Xcc strains. Survival experiments were performed by subculturing strains overnight on fresh NYG agar plates supplemented with different concentrations of SDS (i), phenol (ii), NaCl (iii), and CdCl_2_ (iv). The surviving bacterial colonies on the plates were counted after incubation for 3 days

The results show that ∆*hpaS* produced about 26.9% less EPS than the wild type (Figure 2a and Table S1). Importantly, the EPS yield was restored towards wild‐type levels by complementation of the ∆*hpaS* strain (designated CΔ*hpaS*), where *hpaS* expressed in trans (Figure 2a and Table S1). The Δ*hpaS* mutant also displayed decreased swimming motility (tested on 0.28% wt/vol agar plates) compared to the wild type. As shown in Figure 2b, the diameter of the zones of growth resulting from migration away from the inoculation points on swimming plates were about 2.6 cm for the Δ*hpaS* mutant and 5.2 cm for the wild type. As analysed by the *t* test, the mean radius of the mutant was significantly shorter than that of the wild type (*p* = .05 by *t* test). The diameters of the complemented strain and the wild‐type strain were not significantly different (*p* = .05 by *t* test) (Figure 2b). Interestingly, the ∆*hpaS* mutant produced significantly more extracellular protease than the wild type (*p* = .05 by *t* test), this enhancement in the protease production was not seen in the CΔ*hpaS* strain (Figure 2c‐i). Additionally, endoglucanase and amylase production of the ∆*hpaS* mutant was reduced compared to the wild type (Figure 2c‐ii, iii).

To clarify that mutation in *hpaS* did not influence the growth of Xcc, the growth characteristics of the Xcc strains in liquid nutrient‐rich complex medium NYG and minimal medium MMX were assessed. The growth rates of the Δ*hpaS* mutant did not differ from that of the wild type (Figure S2). In addition, the doubling time was 2.2 hr in NYG medium and 4.8 hr in MMX medium comparable to the wild‐type strain.

In addition, several environmental stresses, including sodium dodecyl sulphate (SDS), phenol (organic solvent exposure), NaCl (hyperosmosis stress), and CdCl_2_ (heavy metal salt), were selected to test the ability of Xcc strains to tolerate these agents (see Experimental procedures). As shown in Figure 2d, the Δ*hpaS* mutant showed significantly decreased survival in the presence of SDS, phenol, and NaCl compared with the wild type (*p* = .05 by *t* test). However, the CΔ*hpaS* strain showed similar survival to the wild type, indicating HpaS contributes to the tolerance of various stresses in Xcc. Taken together, these results reveal that, along with HR and virulence, HpaS contributes to diverse cellular processes, including the production of extracellular enzymes and EPS, cell motility, and the tolerance to various stresses.

### 2.3 HpaS interacts with subset of response regulators including the single domain response regulator VemR

The above data revealed that HpaS has a previously unappreciated regulatory role in Xcc influencing phenotypes associated with virulence, including extracellular enzymes, EPS, and cell motility. Recent work has shown that sensor kinase HpaS and the cognate regulator HrpG play a specific role in controlling HR and virulence during Xcc plant infection (Li *et al*., 2014). However, the scope of regulation by HpaS in Xcc cannot be completely accounted for by HrpG, suggesting other response regulators might be involved in signal transduction from HpaS. To test this hypothesis, we first searched for HpaS‐interacting proteins using co‐immunoprecipitation (co‐IP) coupled with mass spectroscopy (see Experimental procedures).

**Figure 3:**
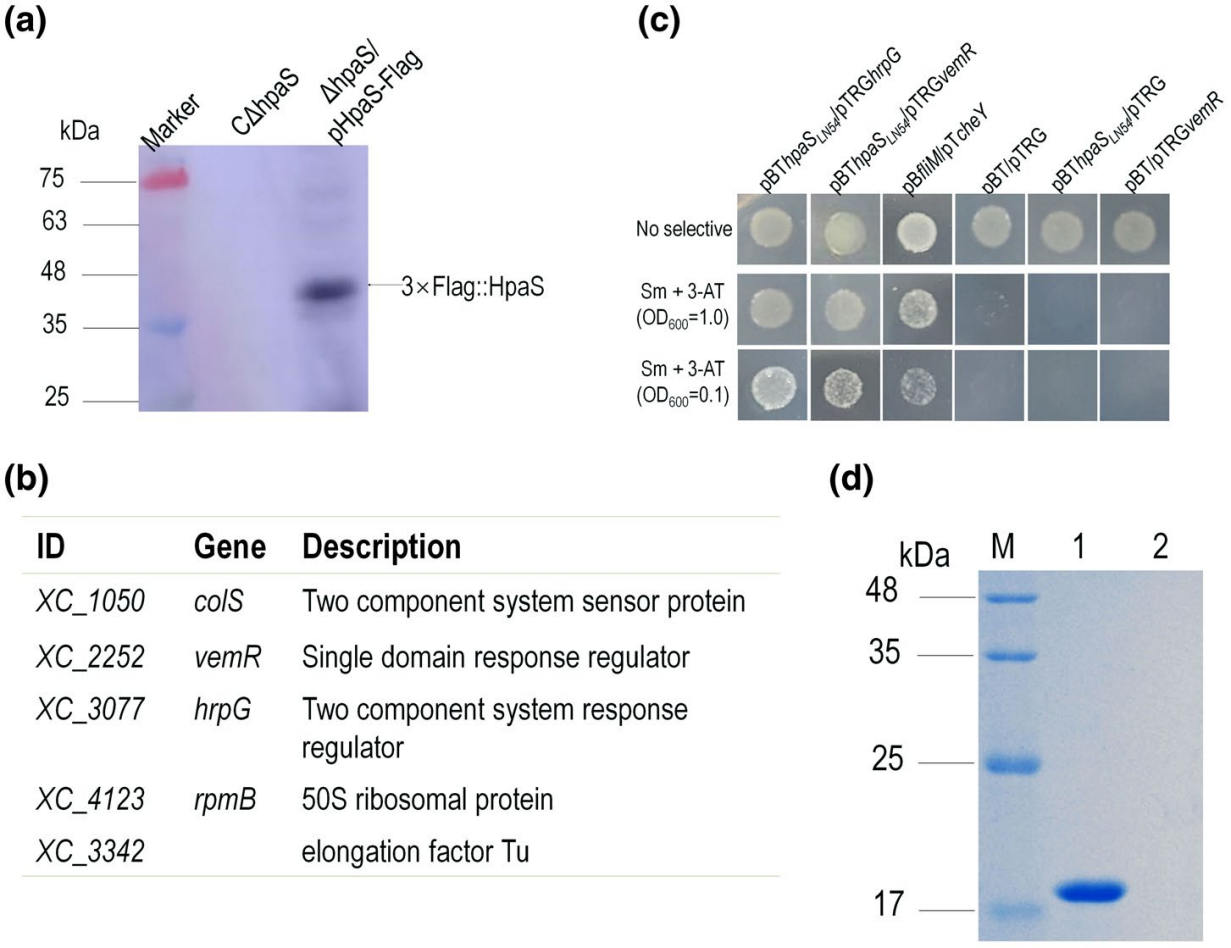
Identification of proteins that interact with the sensor HpaS. (a) Western blot of the eluted 3 × Flag::HpaS fusion protein. After co‐immunoprecipitation, a western blot assay was performed for the eluted 3 × Flag::HpaS fusion protein and control. (b) Candidate target proteins for HpaS identified by mass spectrometry. (c) Bacterial two‐hybrid experiment testing interaction between HpaS and VemR. The reporter strain XL1‐Blue MRF′ with different plasmid pairs was grown on nonselective plates (inoculated with a cell concentration of OD_600_ = 1.0) and double‐selection indicator plates (inoculated with cell concentrations of OD_600_ = 1.0 and 0.1) containing 3‐AT and streptomycin (Sm). Protein–protein interactions activate the expression of *addA* and *HIS3* genes within the reporter gene cassette of the reporter strain, resulting in resistance to 3‐AT and Sm. The reporter strain with plasmid pair pBT*hpaS_LN54_*/pTRG*hrpG* (Li *et al*.,^2014^) was used as positive control. (d) Pull‐down assay showed the interaction between HpaS and VemR. 6 × His‐tagged fusion proteins were overexpressed and purified. Bait protein HpaS was biotinylated and immobilized to streptavidin sepharose beads. The potential prey protein VemR was mixed with the bait protein and incubated. The RavR protein was used as control. After elution, samples were separated on 12% SDS‐PAGE and visualized by Coomassie blue staining. Lane 1, pull‐down of 6 × His::VemR by 6 × His::HpaS; lane 2, biotinylated 6 × His::HpaS was mixed with protein 6 × His::RavR; M, molecular mass marker

For these experiments a strain expressing 3 × Flag‐tagged fusion protein HpaS::3 × Flag was constructed (see Experimental procedures; Table S1) and the complemented strain CΔ*hpaS* (Table S1) was used as a control. A western blot assay confirmed that the 3 × Flag:: HpaS fusion protein complex could be eluted from the strain expressing the 3 × Flag‐tagged fusion protein, but not the control strain CΔ*hpaS* (Figure 3a). Protein complexes with HpaS::3 × Flag within the Δ*hpaS* mutant were purified and analysed by mass spectrometry. This analysis identified five proteins (Figure 3b). Except for the two translation related proteins XC_4123 and XC_3342, these proteins were shown to have roles in signalling and/or motility. ColS is a cognate sensor kinase for ColR that controls a variety of cellular processes (Zhang *et al*., 2008). Interestingly, HrpG, but not HpaR2, was shown to interact with HpaS under this test condition. Notably, VemR was identified to interact with HpaS (Figure 3b). VemR is a monodomain response regulator that contains only a receiver domain and no other signalling domain. This response regulator has been shown to control virulence, EPS production, and motility in Xcc (Tao and He, 2010).

Bacterial two‐hybrid assays were used to validate and extend the observations of the co‐IP analysis. Here, *hpaS* and *vemR* were amplified by PCR and cloned into the bait vector pBT and prey vector pTRG, respectively, and the resulting recombinant plasmids pBT*hpaS*_*LN54*_ and pTRG*vemR* were co‐transformed into the reporter strain XL1‐Blue MRF′ (Table S1). As shown in Figure 3c, the reporter strain XL1‐Blue MRF′ with plasmid pair pBT*hpaS*_*LN54*_ and pTRG*vemR*, similar to the positive control strain harboring pBT*hpaS*_*LN54*_ and pTRG*hrpG* (Li *et al*., 2014), grew on the double‐selective indicator plate containing 5 mM 3‐amino‐1,2,4‐triazole (3‐AT) and 12.5 μg/mL streptomycin (Sm), while the negative control strains (with plasmid pair pBT/pRGT, pBT*hpaS*_*LN54*_/pTRG or pBT/pTRG*vemR*) did not grow, suggesting that the monodomain respond regulator VemR is able to interact with the sensor protein HpaS directly. Additionally, a pull‐down biotinylated protein–protein assay was performed to validate the interaction of HpaS with VemR (see Experimental procedures). The response regulator RavR was used as a negative control. The recombinant tagged proteins HpaS, VemR, and RavR were overexpressed in *E. coli*. After purification, recombinant protein pull‐down assays were performed. The results showthat the HpaS protein could capture VemR, but not RavR (Figure 3d).

Taken together, the data from the co‐IP, bacterial two‐hybrid, and pull‐down assays indicate that the sensor protein HpaS interacts with the response regulator VemR, implying VemR might be involved in the HpaS regulatory network.

### 2.4 The gene *hpaS *shares an epistatic relationship with *vemR* in a regulatory pathway controlling cell motility

The data described above demonstrate that HpaS and VemR could physically interact but the influence of VemR on the regulatory effects of HpaS in Xcc is still to be ascertained. For certain two‐component systems, it has been shown that overexpression of the response regulators in the absence of their cognate kinases can result in constitutive expression of the gene(s) under their control, and restore the phenotypes of the sensor mutant towards the wild type (Powell and Kado, 1990; Aguilar *et al*., 2001; Dong *et al*., 2008).

**Figure 4:**
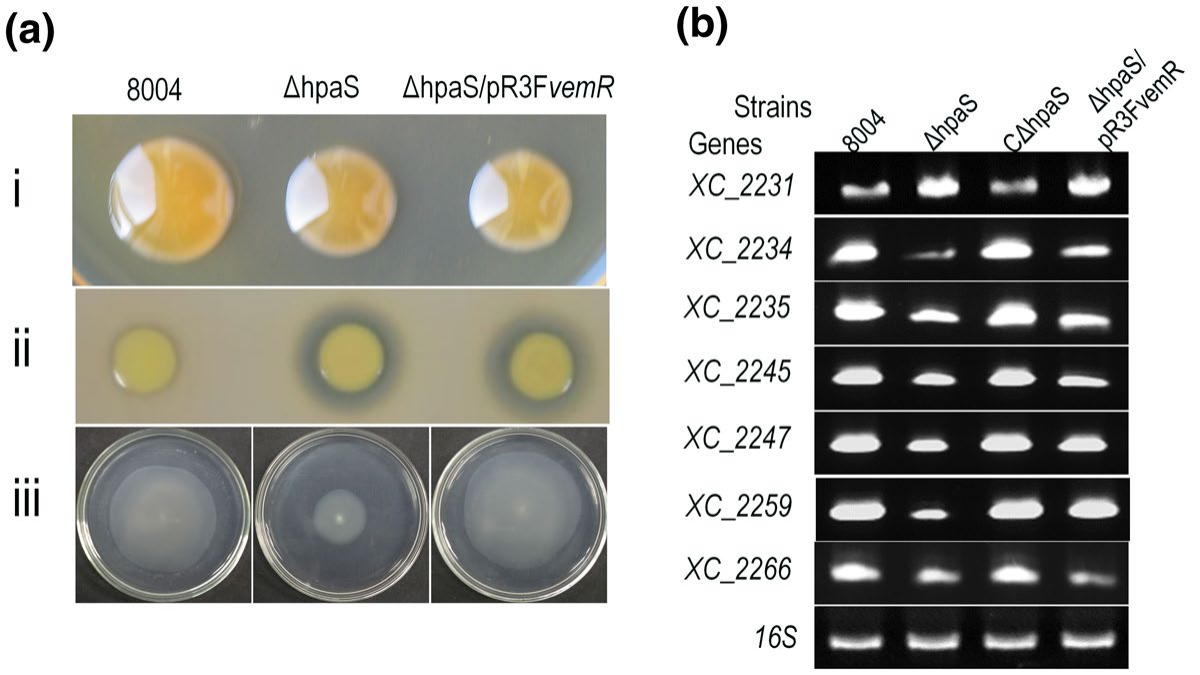
Effects of constitutively expressing *vemR* on the *hpaS* mutant. (a) The constitutively expression of *vemR* restores full cell swimming motility of the *hpaS* mutant. Xcc strains were cultured in NYG medium overnight and 2 µl of bacterial cultures (OD_600_ = 1.0) of each Xcc strain were spotted onto the corresponding agar plates followed by incubation at 28 °C for days. The representative colony morphology of Xcc strains tested for extracellular polysaccharide (EPS) (i), protease activity (ii), and swimming motility (iii) were photographed. The impaired swimming motility in the Δ*hpaS* strain could be restored to the wild‐type level by providing a plasmid bearing the *vemR* gene (strain ∆*hpaS*/pR3F*vemR*). (b) The constitutive expression of *vemR* cannot restore the expression levels of flagellum‐related genes. Xcc strains 8004, Δ*hpaS*, CΔ*hpaS*, and Δ*hpaS*/pR3F*vemR* were cultured in NYG medium to an OD_600_ = 0.6, RNA was extracted from each strain, and cDNA was generated. Semiquantitative RT‐PCR was conducted using the primer sets corresponding to the selected genes

We therefore performed an expression experiment where *vemR* was cloned and overexpressed in the ∆*hpaS* mutant background and assessed phenotype restoration (see Experimental procedures). For this experiment, the plasmid pR3F*vemR* was constructed using a promoterless *vemR* with its authentic ribosome binding site cloned into the vector pLAFR3 in an orientation that allowed the *vemR* to be driven by the *lac* promoter (Table S1). The recombinant plasmid pR3F*vemR* was introduced into the Δ*hpaS* strain by triparental conjugation, obtaining transconjugant strain Δ*hpaS*/pR3F*vemR* (Table S1). The phenotypes of the resulting Xcc strains were analysed. As shown in Figure 4a, Δ*hpaS*/pR3F*vemR* demonstrated similar swimming motility to the wild‐type strain, indicating that constitutive expression of *vemR* restores motility to wild‐type levels in Δ*hpaS*. Additionally, the empty vector pLAFR3 was also introduced into Δ*hpaS* and the wild type, as controls (Table S1). The resulting strains Δ*hpaS*/pLAFR3 and 8004/pLAFR3 presented similar motility with the Δ*hpaS* mutant and wild type, respectively (Figure S3).

The effect of constitutive expression of *vemR* on the expression of flagellum‐related genes in the *hpaS* mutant background was further explored. The expression level of selected genes were compared among the Xcc wild‐type 8004, Δ*hpaS*, and the Δ*hpaS*/pR3F*vemR* strains using semiquantitative RT‐PCR (see Experimental procedures). Interestingly, the expression level of flagellum‐related genes could not be restored by the constitutive expression of *vemR* in the *hpaS* mutant background (Figure 4b).

To further ascertain that HpaS controls swimming motility via VemR, we generated several strains carrying deletions (see Experimental procedures). We created a *vemR* deletion mutant (designated Δ*vemR*) and a *vemR* and *hpaS* double‐deletion mutant (designated Δ*hpaS*Δ*vemR*) (Table S1).

**Figure 5:**
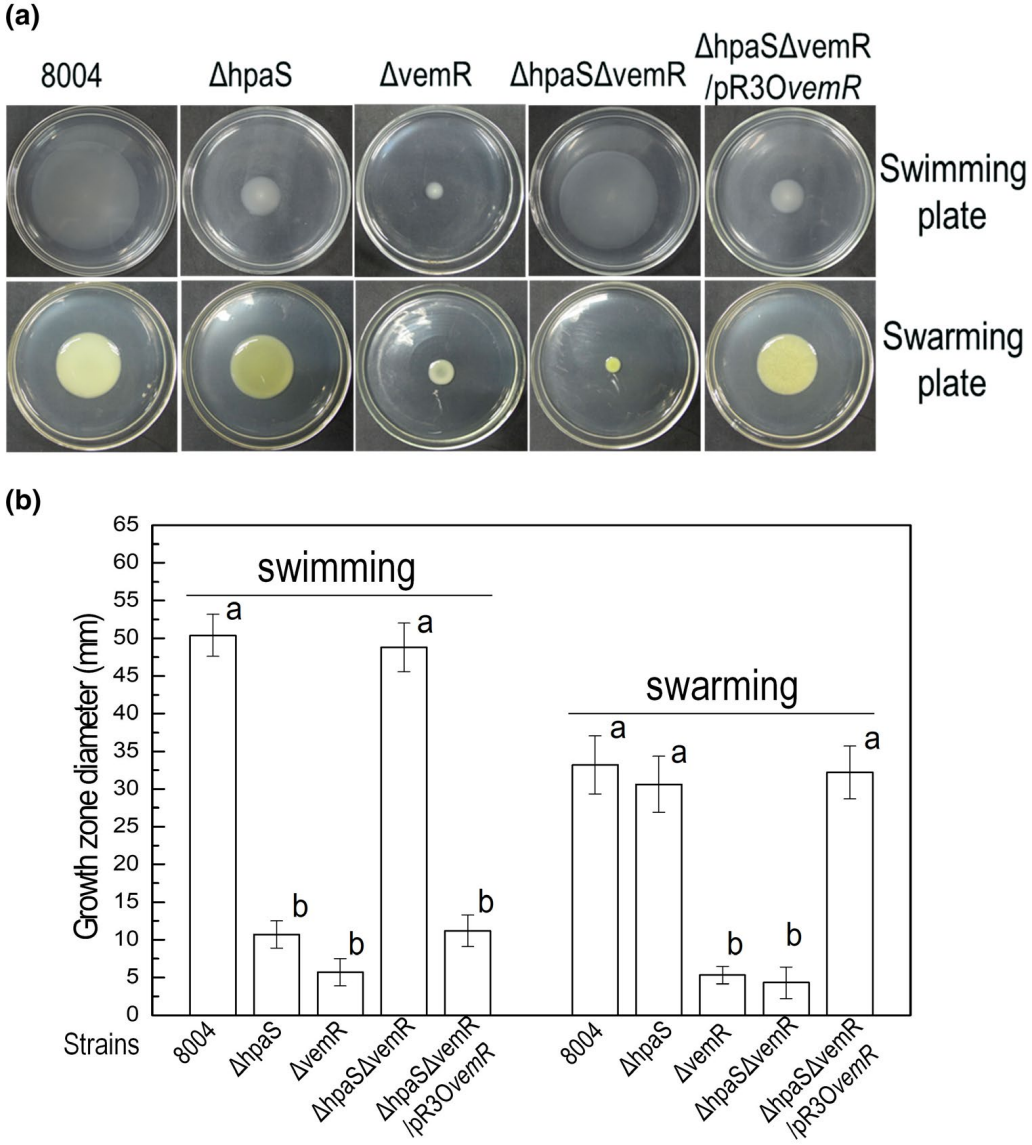
*hpaS*/*vemR* double‐deletion mutant revealed the wild‐type swimming motility. *Xanthomonas campestris* pv. *campestris* (Xcc) strains were stabbed into “swim” (0.28% agar) medium followed by incubation at 28 °C for 4 days or inoculated onto “swarm” (0.6% agar) plate and incubated for 3 days. The representative colony morphologies of Xcc strains were photographed (a) and colony diameters of each strain on the different media were measured (b). Values given are the means ± *SD* of triplicate measurements from a representative experiment, similar results were obtained in two other independent experiments. The different letters on each column indicate significant differences at *p* = .05 by *t* test. The *hpaS* and *vemR* single mutant strain present significant small colonies on swimming plates. However, the double‐deletion mutant Δ*hpaS*Δ*vemR* grows in a similar fashion to the wild type

The Δ*vemR* showed reduced swimming and swarming motility compared to the wild type (Figure 5a,b). These phenotypic changes are similar to those reported by Tao and He (2010). Interestingly, the Δ*hpaS*Δ*vemR* strain displayed similar swimming motility to the wild type, but swarming motility was reduced (Figure 5a,b). Additionally, *vemR* was introduced back into the Δ*hpaS*Δ*vemR* strain. This was achieved by introducing the plasmid pR3O*vemR* into the Δ*hpaS*Δ*vemR* strain (Table S1). The plasmid pR3O*vemR* was constructed by introducing the 381‐bp DNA fragment of the *vemR* coding sequence into the plasmid pLAFR3 (see Experimental procedures). The resulting strain Δ*hpaS*Δ*vemR*/pR3O*vemR*, when tested for motility, showed similar phenotypes to the Δ*hpaS* strain (Figure 5a,b). Together, these data suggest that the *vemR* gene has a special epistatic relationship with the *hpaS* gene and the suppressive effect of HpaS mutation on swimming motility relies on VemR. VemR may function downstream of HpaS in the signalling pathway that controls Xcc swimming motility.

### 2.5 VemR binds to the flagellum protein FliM

In many motile bacteria, the direction of rotation of flagellum is controlled by a complex of proteins at the bottom of the basal body called the switch complex, formed from multiple subunits of the proteins FliG, FliM, and FliN. Besides controlling rotation, the switch complex is also essential for flagellar assembly and the generation of torque (for review, see Porter *et al*., 2011). Several response regulators, in particular the monodomain response regulator CheY, have been shown to regulate motility by interactions with switch complex of the flagellum. Given that several flagellum‐related genes were down‐regulated in the ∆*hpaS* mutant, and that the reduced expression level of these genes in *hpaS* mutant could not be restored by the constitutive expression of *vemR*, VemR may potentially influence swimming (flagellum‐dependent) motility through the interaction with components of the flagellar motor switch complex.

**Figure 6:**
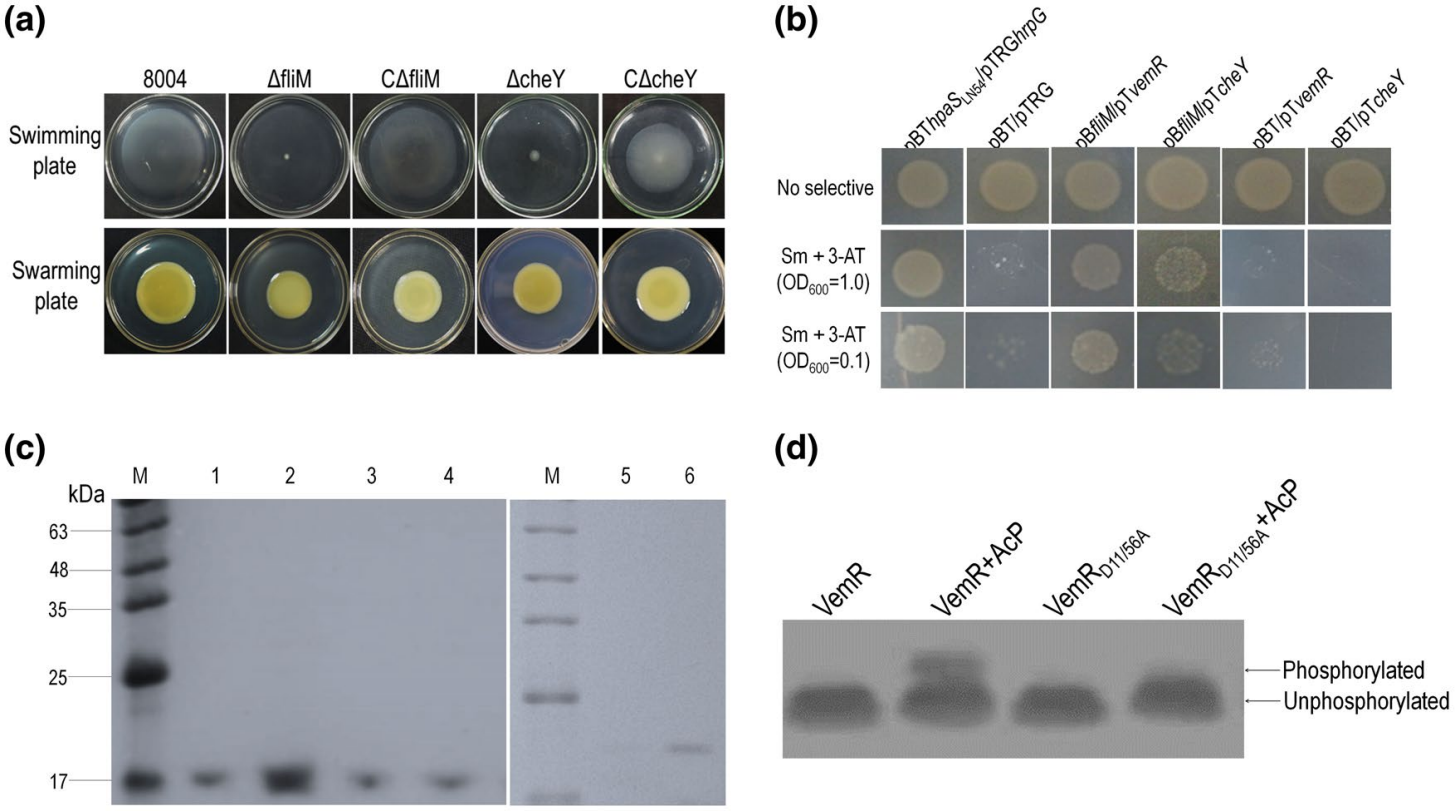
VemR interacts with the FliM component of the flagellar switch complex. (a) FliM and CheY are essential for cell swimming motility in *Xanthomonas* *campestris* pv. *campestris* (Xcc). Xcc wild‐type strain 8004, *fliM* deletion mutant strain (∆*fliM*), *cheY* deletion mutant strain (∆*cheY*), and the complemented strains C∆*fliM* and C∆*cheY* were stabbed into “swim” (0.28% agar) medium, respectively, followed by incubation at 28 °C for 4 days or inoculated onto “swarm” (0.6% agar) plate and incubated for 3 days. The representative colony morphology of Xcc strains were photographed. The ∆*fliM* and ∆*cheY* mutants formed tiny colonies on swimming plates compared to the wild type, but formed normal colonies on swarming plates. (b) Bacterial two‐hybrid assays indicate that interaction exists between FliM and VemR. The BacterioMatch II two‐hybrid system was used to test the interaction of FliM with VemR. The reporter strain XL1‐Blue MRF′ harbouring different plasmid pairs was grown on nonselective plates (inoculated with a cell concentration of OD_600_ = 1.0) and double‐selection indicator plates (inoculated with a cell concentration of OD_600_ = 1.0 and 0.1) containing 3‐amino‐1,2,4‐triazole (3‐AT) and streptomycin (Sm), respectively. Protein–protein interaction would activate the expression of the genes *HIS3* and *addA* in the reporter strain, resulting in resistance to 3‐AT and Sm. The reporter strain with plasmid pair pBT*hpaS*_LN54_/pTRG*hrpG* (Li et al., 2014) was used as positive control*.* The reporter strain with plasmid pair pBT*fliM*/pTRG*vemR* or pBT*fliM*/pTRG*cheY* formed poor colonies on the selective plates. (c) Pull‐down assays showed the interaction of FliM with phosphorylated VemR and CheY. 6 × His‐tagged fusion proteins were overexpressed and purified. Bait protein FliM was biotinylated and immobilized to streptavidin sepharose beads. The potential prey protein (VemR or CheY) with or without acetyl phosphate (AcP) was mixed with the bait protein and incubated. After elution, samples were separated on 12% SDS‐PAGE and visualized by Coomassie blue staining. Lane 1, biotinylated FliM::6 × His was mixed with protein 6 × His::VemR; lane 2, biotinylated FliM::6 × His was mixed with 6 × His::VemR treated with AcP; lane 3, biotinylated FliM::6 × His was mixed with mutant protein 6 × His::Vem_RD11/56A_; lane 4, biotinylated FliM::6 × His was mixed with mutant protein 6 × His::VemR_D11/56A_ treated with AcP; lane 5, biotinylated FliM::6 × His was mixed with protein 6 × His::CheY; lane 6, pull‐down of phosphorylated protein 6 × His::CheY by FliM::6 × His; M, molecular mass marker. (d) In vitro phosphorylation assays revealed that VemR, but not the mutant form VemR_D11/56A_, could be phosphorylated by AcP. Wild‐type VemR (5 μg) and its mutant were incubated with 50 mM AcP for 30 min at 37 °C, followed by detection on Phos‐tag SDS/PAGE gels. Controls included a reaction containing only VemR protein and no phosphorylation by AcP was observed. Bands for phosphorylated forms are shown by arrows on the right‐hand side of the panel

To examine this, FliM (XC_2267) was selected to test its potential interactions with VemR as FliM is essential for the swimming motility in Xcc (Figure 6a). Interactions of CheY (XC_2282) with FliM were also tested and used as a control. Xcc CheY, similar to its counterpart in *E. coli* (for a review, see Szurmant and Ordal, 2004), regulates swimming motility (Figure 6a). Amino acid sequence pairwise alignments using Vector NTI showed that VemR and CheY in Xcc share only 17.7% identity and 31.5% similarity (Figure S4). To test these interactions, bacterial two‐hybrid assays were employed (see Experimental procedures). The 1011‐bp *fliM* gene and 378‐bp *cheY* were amplified by PCR, and cloned into the bait vector pBT, respectively, generating the plasmids pBT*fliM* and pBT*cheY* (Table S1). Interactions were assessed in the reporter strain XL1‐Blue MRF′ (see Experimental procedures). Tests revealed that the plasmid pair pBT*fliM* and pTRG*vemR* grew weakly on the double‐selection indicator plate containing 3‐amino‐1,2,4‐triazole (3‐AT) and Sm (Figure 6b), indicating an interaction between FliM and VemR. This potential interaction was further tested by pull‐down biotinylated protein–protein assays (see Experimental procedures). For this, recombinant 6 × His‐tagged proteins FliM and VemR were overproduced and purified. The *cheY* (*XC_2282*) gene was also cloned into the expression vector to produce 6 × His‐tagged CheY to be used as a control. As shown in Figure 6c, FliM could weakly capture VemR (Figure 6c, lane 1). However, when the acetyl phosphate (AcP) was added to the reaction mix in the pull‐down biotinylated protein–protein assay, FliM captured more VemR (Figure 6c, lane 2), suggesting the interaction depends on the phosphorylation status of VemR.

According to the vast literature about response regulator activation, the aspartyl residue at position 11 in VemR is predicted to be the phosphorylation site. This residue most likely plays a key role in coordinating the catalytic Mg^2+^ cation at the reaction site (for a review, see Gao *et al*., 2019). However, the aspartyl residue at position 56 is also probably phosphorylated (Tao and He, 2010). To verify these assumptions, aspartyl residues at positions 11 and 56 were replaced with alanine in VemR (see Experimental procedures). The resulting variant VemR_D11/56A_ was purified (see Experimental procedures). The wild‐type VemR and the variant VemR_D11/56A_ were incubated with AcP, which specifically phosphorylates the acceptor aspartyl residues of certain response regulators. As shown in Figure 6d, wild‐type VemR could be phosphorylated by AcP. However, no phosphorylation of the variant VemR_D11/56A_ was observed, indicating aspartyl residues at positions 11 and/or 56 are important for phosphorylation. Additionally, interactions between FliM and the variant VemR_D11/56A_ with and without AcP were tested. As shown in Figure 6c, the amounts of VemR_D11/56A_ captured by FliM were similar to that of the wild‐type VemR (Figure 6c, lanes 1 and 3). When the AcP was added, no enhancement of captured VemR_D11/56A_ was observed (Figure 6c, lane 4), indicating that the phosphorylation is important for the VemR–FliM interaction. Overall, these combined data suggest that the phosphorylated VemR interacts with the basal body flagellum protein FliM, which is important for swimming motility in Xcc.

### 2.6 The phosphorylation of VemR is partly dependent on the presence of HpaS

No cognate kinase sensor has been identified for the orphan response regulator VemR (Tao and He, 2010). The above data suggest that HpaS interacts with VemR. This prompted us to directly test the phosphoryl group transfer between HpaS and VemR. In vitro phosphotransfer assays were conducted using the method previously described (Li *et al*., 2014). Equal amounts (1 μM) of 6 × His::VemR were added to a reaction mixture in which 6 × His::HpaS_LN54_ was autophosphorylated with [γ‐^33^P]ATP. After 10, 20, and 30 min incubation, no radioactively labelled 6 × His::VemR was observed (data not shown), indicating that HpaS could not transfer the phosphate group to VemR under our test conditions.

**Figure 7:**
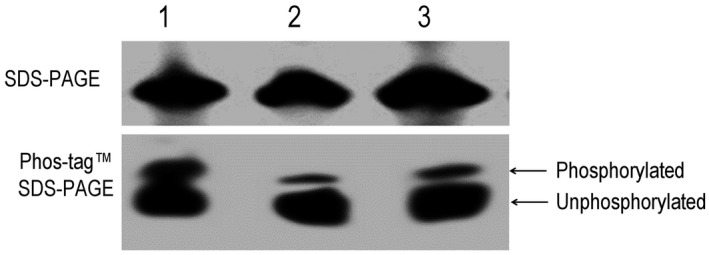
HpaS has an impact on the phosphorylation of VemR in vivo. *Xanthomonas campestris* pv. *campestris* strains were cultured in NYG medium to OD_600_ = 0.6, and bacterial cells from 10 ml culture were harvested, transferred to 100 ml MMX medium, and cultured until OD_600_ = 0.6. Total proteins were prepared and separated on SDS‐PAGE and Phos‐tag SDS‐PAGE gels, respectively, and then electrotransferred onto PVDF membrane for western blotting. The primary antibody was anti‐His‐tag antibody (Qiagen) that was used at a 1:2,500 dilution according to manufacturer's instructions. Binding of the primary antibody was detected using goat anti‐mouse IgG horseradish peroxidase conjugated secondary antibody (Bio‐Rad). Phosphorylated and unphosphorylated VemR proteins were separated by Phos‐tag SDS‐PAGE gel. Lane 1, 10 μg protein from strain Δ*vemR*/pHis*vemR*_lac_; lane 2, 10 μg protein of strain Δ*hpaS*Δv*emR*/pHis*vemR*_lac_, lane 3, 20 μg protein of strain Δ*hpaS*Δ*vemR*/pHis*vemR*_lac_

It is possible that phosphotransfer from HpaS to VemR requires specific conditions that were lacking in our in vitro experiments but potentially exist in vivo. We therefore tested whether the presence and absence of HpaS is important for VemR phosphorylation in vivo. To achieve this, a single mutant lacking *vemR* (Δ*vemR*) and a double mutant lacking both *vemR* and *hpaS* (Δ*hpaS*Δ*vemR*) were used (Table S1). Simultaneously, a 381‐bp DNA fragment containing a promoterless *vemR* open reading frame (ORF) was fused with a 6 × His‐tag coding sequence amplified by PCR and cloned into the vector pLAFR3. The resulting recombinant plasmid pHis*vemR*_lac_ was introduced into the Δ*vemR* and Δ*hpaS*Δ*vemR* strains, respectively, creating strains Δ*vemR*/pHis*vemR*_lac_ and ∆*hpaS*Δ*vemR*/pHis*vemR*_lac_ (Table S1) that constitutively express 6 × His‐tagged VemR. The phosphorylation of VemR protein in these strains was examined using the Phos‐tag SDS‐PAGE method (see Experimental procedures). As shown in Figure 7, when the same amount (10 μg) of total protein was loaded into the gel, the bands representing total VemR protein (SDS‐PAGE gel) from both strains were similar. However, the band representing the phosphorylated VemR protein from strain ∆*hpaS*Δ*vemR*/pHis*vemR*_lac_ was markedly weaker than that from strain Δ*vemR*/pHis*vemR*_lac_, implying that HpaS mutation blocks the phosphorylation of VemR. Additionally, a strain expressing Mip with a 6 × His‐tag control (Li *et al*., 2014) was also used as a negative control, and no phosphorylated Mip protein was found on Phos‐tag SDS‐PAGE gel (data not shown).

Although HpaS was unable to phosphorylate VemR in vitro, the protein affected the response regulator's phosphorylation in vivo. This suggests that under certain conditions HpaS probably transfers the phosphoryl group to the orphan regulator VemR, either directly or indirectly.

## 3 DISCUSSION

In general, a sensor kinase protein perceives signals and phosphorylates a downstream response regulator protein in order to control downstream gene expression and associated phenotypes. Our previous work demonstrated that in Xcc the sensor kinase HpaS is required for virulence and HR induction, and that HpaS forms a two‐component pathway with the response regulator HrpG that controls the T3SS (Li *et al*., 2014). In the current work we expand on these observations to show that the Xcc HpaS sensor kinase has a much greater scope of regulation than previously envisaged, involved in controlling the expression of an extensive number of genes and influencing Xcc virulence. We also demonstrate that HpaS is implicated in regulating distinct downstream functions in Xcc through a second response regulator, VemR. We provide evidence that HpaS exerts its modulatory action probably via VemR phosphorylation, either directly or indirectly. VemR in turn interacts with the flagellum motor protein FliM, eventually controlling swimming motility.

Previous studies identified and characterized the regulatory role of the orphan response regulator VemR. The *vemR* gene was shown to encode a standalone receiver (REC) domain protein and to reside in an operon that consists of the *rpoN2, vemR*, and *fleQ* genes. The *rpoN2* gene encodes a σ^54^ factor that is involved in nitrogen assimilation. The *fleQ* gene also encodes a σ^54^ factor that is essential for normal flagellation and transcription of the promoters of the *fliE*, *fliL*, *fliQ*, *flgB*, *flgG*, *flhF*, and *flhBA* genes in Xcc (Tao and He, 2010). Phenotyping demonstrated that mutation of the *vemR* gene severely affected Xcc virulence, EPS production, and motility. Interestingly, the study also demonstrated that the double‐deletion mutant Δ*vemR*/Δ*fleQ* had a phenotype similar to the single mutant Δ*fleQ*, indicating that *fleQ* might have an epistatic relationship with *vemR* in the regulation of virulence and adaptation (Tao and He, 2010). In the current study, we confirmed the regulatory role of VemR in virulence and adaptation in Xcc as seen by Tao and He (2010)**.** Additionally, we demonstrate that VemR plays an important role in controlling swimming motility in Xcc and present evidence that VemR specifically interacts with the flagellar basal body protein FliM, a previously unknown interaction. FliM is important for swimming motility in all flagellated bacteria*.*

Although monodomain response regulators are widespread in bacteria and form the second‐largest class of response regulators (Jenal and Galperin, 2009), knowledge about their cellular functions is still limited. So far, the best characterized monodomain response regulator is the chemotaxis protein CheY from *E. coli* (for review, see Szurmant and Ordal, 2004). In *E. coli* chemotaxis, stimuli at the receptors control autophosphorylation of the histidine kinase CheA, CheA being responsible for transferring the phosphoryl group to CheY. Phosphorylated CheY interacts with the FliM component of the flagellar switch complex, causing a change in flagellar rotation from counterclockwise to clockwise. Here we showed that the Xcc CheY controls swimming motility, and phosphorylation of CheY increases its affinity for FliM. However, the homology between CheY and VemR is relatively low (Figure S4), implying that VemR might function differently from FliM. Although the effect of FliM binding by VemR remains to be determined, we speculate that VemR might compete for FliM binding with CheY, which is known to control motor rotation. VemR and CheY might thus contribute to signal integration from the histidine kinases HpaS and CheA. Notably, our results also reveal that CheY in Xcc is essential in swimming motility, but not swarming motility. However, VemR controls both swimming and swarming motilities. This swarming effect of VemR seems to be irrelevant with the VemR–FliM interaction. We believe that VemR regulates the swarming motility via an HpaS‐independent signal pathway.

The function of the response regulators in most cases is controlled by phosphorylation, which is regulated by multiple enzyme activities, including phosphotransfer from cognate histidine sensor kinase or histidine phosphotransferase, dephosphorylation by auxiliary phosphatases, and autophosphorylation by small‐molecule phosphodonors such as phosphoramidate and AcP (for a review, see Gao *et al*., 2019). Until now the cognate histidine kinase of VemR has not been identified. Here we show that the constitutive expression of *vemR* restores full swimming motility to *hpaS*‐null mutant and that a double deletion of *vemR* and *hpaS* resulted in the wild‐type swimming motility phenotype, suggesting that HpaS controls swimming motility most likely via VemR protein. We also show that HpaS interacts with VemR and reduces the phosphorylation of VemR in vivo. Commonly, genes encoding both components are co‐located in the genome. However, orphan sensor kinases and response regulators have also been described. Based on the biochemical and biophysical relationship between HpaS and VemR, we suppose that HpaS is responsible for transferring the phosphoryl group to the orphan response regulator VemR, either directly or indirectly. Under certain conditions HpaS and VemR are likely to form an atypical two‐component pathway to control the swimming motility. Therefore, under certain environmental conditions HpaS autophosphorylates and directly or indirectly transfers phosphate group to VemR. It is then that phosphorylated VemR interacts with the basal body protein FliM to modulate flagellar rotation and control the swimming motility.

It does appear that VemR could exert its regulatory activity through protein–protein interactions depending on its phosphorylation state. Interestingly, Tao and He (2010) demonstrated that a deletion of *fleQ* encoding a σ^54^ factor cognate activator in a *vemR*‐mutant background restores the EPS and motility to the wild type, suggesting that FleQ functions downstream of VemR in the signalling pathway that controls EPS and motility (swimming and swarming). To confirm this, a double‐deletion mutant of *vemR*/*fleQ* was also constructed in our work and similar results were obtained (data not shown). Taking this information together, we suggest that VemR has a dual regulatory influence on swimming motility. Besides being the terminal response regulator of the HpaS/VemR pathway, VemR is probably an intermediary in a multistep signal cascade containing FleQ that regulates EPS production and swimming and swarming motilities in Xcc. This signalling cascade may be independent of HpaS but this has yet to be determined.

**Figure 8:**
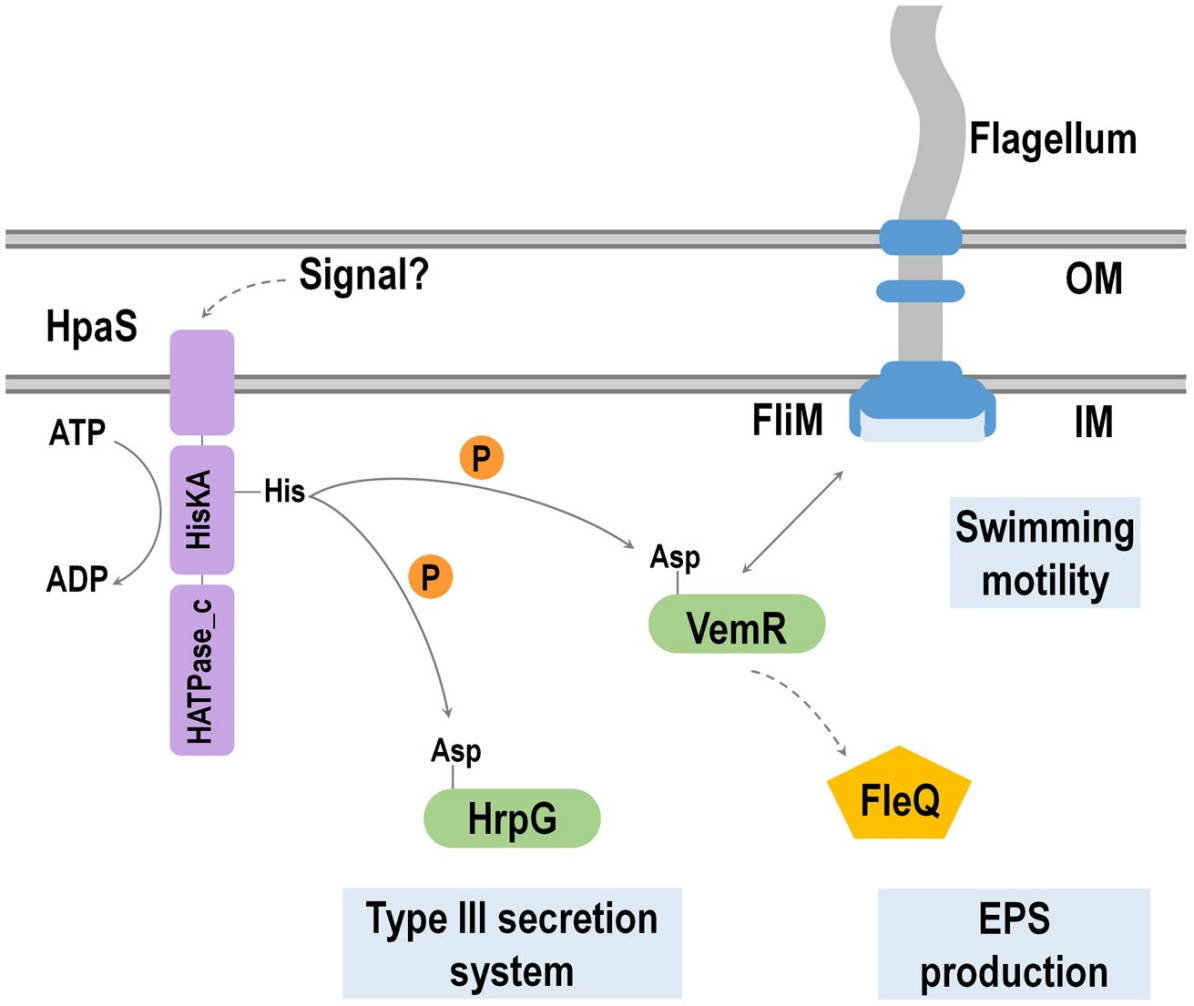
Model for multiple pathways of signal transduction following signal recognition by HpaS in *Xanthomonas campestris*. Signal perception and signal transduction involving the sensor kinase HpaS and response regulator HrpG act to coordinately regulate the type III secretion system and virulence. This study identifies that HpaS also regulates a wide range of addition phenotypes, including extracellular polysaccharide (EPS) production and motility. Interestingly, HpaS appears to co‐opt a second an orphan response regulator VemR in order to regulate swimming motility. VemR appears to achieve this via interaction with the flagellum protein FliM. Additionally, VemR appears to control EPS production independent of HpaS but involving the regulator FleQ. How VemR regulates this process is the subject of further experiments

Many branched two‐component systems probably exist in diverse bacteria. An analysis of predicted two‐component signalling protein‐encoding genes in 207 genomes revealed that there is a large disparity between the number of response regulators (RRs) and the number of histidine kinases (HKs) in most bacteria (Alm *et al*., 2006). In *E. coli*, a total of 30 sensor HKs and 34 RRs have been suggested to exist. Phosphotransfer analysis revealed that seven of the HKs phosphorylated more than one response regulator(s) (Mizuno, 1997; Yamamoto *et al*., 2005). Analysis of sequenced genomes of Xcc strains ATCC33913, 8004, and B100 revealed that this bacterium encodes 32 HKs sensors, 54 RRs, and 20 proteins with HPt domains (da Silva *et al*., 2002; Qian *et al*., 2005; Vorhölter *et al*., 2008). This analysis suggests that branched two‐component pathways of “one‐to‐many” structures exist in Xcc. However, to date no reports have described such a pathway in Xcc or other *Xanthomonas* species for that matter. In the current study, we show that along with the previously described sensor kinase and response regulator pairing of HpaS and HrpG in order to control virulence and HR, HpaS also co‐opts another orphan response regulator VemR to specifically function on motility. Combined with our previous work (Li *et al*., 2014), we hypothesize that HpaS/HrpG/VemR may represent a “one‐to‐many” branched two‐component pathway in which the sensor kinase HpaS regulates T3SS and swimming motility by controlling the phosphorylation of HrpG and VemR, respectively (Figure 8). This is the first report of a sensor controlling multiple phenotypes via different response regulators in *Xanthomonas* spp.

The work described suggests that the sensor kinase HpaS and orphan response regulator VemR are a two‐component pathway that contributes to the control of swimming motility in Xcc*.* Despite these observations, additional work is required to examine the many aspects of this complex signalling pathway. Relevant questions include: What are the specific environmental or host signals that activate the expression and activity of HpaS? What are the specifics behind HpaS phosphorylation of VemR (and HrpG)? Does HpaS interact with other target proteins? Can we gain structural insight into how VemR interacts with FliM? How does this VemR interaction influence FliM action? Overall, this work illustrates the uncharacterized regulatory role of HpaS and the previously unappreciated role that this sensor kinase contributes to a branched two‐component pathway involving the response regulators VemR, HrpG, and HpaR2 in the control of motility and other cellular processes.

## 4 EXPERIMENTAL PROCEDURES

### 4.1 Bacterial strains, plasmids and growth conditions

The bacterial strains and plasmids used in this study are listed in Table S1. *E. coli* strains were grown in in Luria Bertani medium (Miller, 1972) or M9 (67.8 g Na_2_HPO_4_, 30 g KH_2_PO_4_, 5 g NaCl, 10 g NH_4_Cl per litre) at 37°C. Xcc strains were grown at 28 °C in NYG medium (Daniels *et al*., 1984b), NY medium (NYG medium but without glycerol), and minimal medium MMX (Daniels *et al*., 1984a). Antibiotics were added at the following concentrations when required: kanamycin (Kan) 25 μg/ml, rifampicin (Rif) 50 μg/ml, ampicillin (Amp) 100 μg/ml, spectinomycin (Spc) 50 μg/ml, gentamicin (Gm) 5 μg/ml, Sm at 100 μg/ml, and tetracycline (Tet) 5 μg/ml for Xcc strains and 15 μg/ml for *E. coli*.

### 4.2 DNA and RNA manipulations

DNA manipulations followed the procedures previously described (Sambrook *et al*., 1989). Conjugation between Xcc and *E. coli* strains was performed as previously described (Turner *et al*., 1985). The restriction endonucleases, T4 DNA ligase and *Pfu* polymerase were provided by Promega. The total RNA was extracted from Xcc strains using a total‐RNA extraction kit (Invitrogen) and cDNA generated using a cDNA synthesis kit (Invitrogen). For semiquantitative RT‐PCR, the obtained cDNA was diluted and used as template with selected primers for target genes (Table S4).

### 4.3 Deletion mutant construction and complementation

HpaS single‐deletion mutant ∆*hpaS* has been constructed in previous work (Li *et al*., 2014). For cross‐complementation of the deletion mutant ∆*hpaS* (or double‐deletion mutant Δ*hpaS*Δ*vemR*), a 434‐bp (or 381‐bp) fragment of *vemR* gene was PCR‐amplified with the primer set pR3*VemR*‐F/R (or *vemR‐*OF/R) (Table S4) from Xcc wild‐type strain 8004, and cloned into the vector pLAFR3, resulting plasmid named pR3F*vemR* (or pR3O*vemR*) (Table S1). The recombinant plasmids were introduced into the mutant strain Δ*hpaS* (or double mutant strain Δ*hpaS*Δ*vemR*), generating recombinant strain ∆*hpaS*/pR3F*vemR* (or Δ*hpaS*Δ*vemR*/pR3O*vemR*) (Table S1).

To construct the *vemR* (*XC_2252*) deletion mutant, 510‐bp upstream and 526‐bp downstream fragments flanking the ORF of *vemR* were amplified with the primer sets LvemR‐F/R and *RvemR‐*F/R (Table S4), respectively. The two fragments were cloned together into the *Eco*RI/*Pst*I sites of pK18*mobsacB* (Schäfer *et al*., 1994). The resulting recombinant plasmid pK18*mobsacBvemR* was introduced into the Xcc wild‐type strain 8004 by triparental conjugation. The transconjugants were screened on selective agar plates containing 5% sucrose. The obtained *vemR* deletion mutant was further confirmed by PCR and named Δ*vemR* (Table S1). For constructing *fliM* single‐deletion mutant ∆*fliM* (Table S1), 426‐bp upstream and 294‐bp downstream fragments flanking the ORF of *fliM* were amplified using primer sets LfliM‐F/R and RfliM‐F/R (Table S4), and the obtaining DNA fragments were cloned together into *Kpn*I/*Hin*dIII sites of pK18*mobsacB*. For constructing *cheY* single‐deletion mutant ∆*cheY* (Table S1), 495‐bp upstream and 423‐bp downstream fragments flanking the ORF of *cheY* were amplified using primer sets LcheY‐F/R and RcheY‐F/R (Table S4), and the obtained DNA fragments were cloned together into *Eco*RI/*Hin*dIII sites of pK18*mobsacB*.

To construct the *hpaS*/*vemR* double‐deletion mutant, the 412 bp upstream and 475 bp downstream fragments flanking *hpaS* were amplified with the primer sets SU‐F/R and *SD*‐F/R (Li *et al*., 2014), respectively, using the total DNA of Xcc strain 8004 as a template. Simultaneously, Gm‐resistant fragment was amplified with the primer set GF/R (Li *et al*., 2014), using plasmid pPH1JI as a template. These three fragments were cloned into the *Eco*RI‐*Kpn*I‐*Xba*I‐*Pst*I sites of pK18*mobsacB* (Schäfer *et al*., 1994), and the resulting plasmid named pK18*mobsacBhpaS* was introduced into the *vemR* deletion mutant Δ*vemR* by triparental conjugation. The obtained double‐deletion mutant was named Δ*hpaS*Δ*vemR* (Table S1).

To complement the double‐deletion mutant Δ*hpaS*Δv*emR* and deletion mutant Δ*vemR* with a DNA fragment producing 6 × His‐tagged form of VemR, a 381‐bp DNA fragment of *vemR* gene coding sequence fused with 6 × His‐tag encoding sequences was amplified with the primer set *vemR*‐HisF/R (Table S4). The obtained PCR fragment was cloned into the *Bam*HI/*Hin*dIII sites of pLAFR3 (Staskawicz *et al*., 1987). The resulting recombinant plasmid pHis*vemR_lac_* was introduced into the mutant strains Δ*vemR*Δ*hpaS* and Δ*vemR*, respectively, generating reporter strains Δ*hpaS*∆*vemR*/pHis*vemR_lac_* and ∆*vemR*/pHis*vemR_lac_*_._ (Table S1). To complement the *fliM* and *cheY* deletion mutants, a 1011‐bp fragment of *fliM* and 378‐bp *cheY* were PCR‐amplified with the primer set fliM‐OF/R and OcheY‐F/R (Table S4), respectively, and cloned into the vector pLAFR3, resulting in plasmids pR3O*fliM* and pR3O*cheY* (Table S1).

### 4.4 Site‐directed mutagenesis

Site‐directed mutagenesis was carried out a QuikChange II Site‐Directed Mutagenesis kit (Stratagene). The gene *vemR* was cloned into pK18*mob* (Schäfer *et al*., 1994) and amplified by the specific PCR with mutagenic oligonucleotides (primer sets D11‐F/R and D56‐F/R, see Table S4), and the recombinant plasmid pK*vemR* as template. Then, the PCR products were digested with *Dpn*I and transformed into *E. coli* DH5a, resulting in the recombinant plasmid containing *vemR* with two codons mutated (named pK*vemR_D11/56A_*, see Table S1). The mutated *vemR* genes were then PCR‐amplified and cloned into pQE‐30. The variant VemR protein (named VemR_D11/56A_) with position 11 and 56 aspartate residues replaced by alanine was overproduced and purified.

### 4.5 Overproduction and purification of proteins

To overproduce the 6 × His‐tagged form of VemR, FliM, and CheY, the 381‐bp *vemR*, 1011‐bp *fliM*, and 378‐bp *cheY* coding sequences were PCR‐amplified from Xcc wild‐type strain 8004 using the primer sets *vemR‐*OF/R, *fliM*‐OF/R, and *cheY*‐OF/R (Table S4), respectively. The obtained DNA fragments were cloned into the expression vectors pQE‐30, pET‐32a, and pET‐30a to generate the recombinant plasmids pQE‐30‐VemR, pET‐32a‐FliM, and pET‐30a‐CheY (Table S1). The recombinant plasmids pQE‐30‐VemR (or pQE‐30‐VemR_D11/56A_, producing VemR_D11/56A_), pET‐32a‐FliM, and pET‐30a‐CheY were transformed into *E. coli* M15 or BL21, resulting in strains M15/pQE‐30‐VemR (or pQE‐30‐VemR_D11/56A_), BL21/pET‐32a‐FliM, and BL21/pET‐30a‐CheY, respectively. Strains were cultured to an OD_600_ = 0.6, and then induced by the addition of isopropyl β‐d‐thiogalactopyranoside (IPTG) at a final concentration of 0.2 mM. After the culture had been grown for a further 4 hr, the cells were harvested and the fused protein was purified using Ni‐NTA resin (Qiagen). To obtain the HpaS and RavR proteins, *E. coli* strains M15/pQE‐HpaS_LN54_ and M15/pQE‐RavR expressing truncated HpaS (amino acids 55–143, excluding the N‐terminal 54 amino acid transmembrane domain) and RavR (amino acids 22–573), respectively, with a 6 × His‐tag on its N‐terminus (Li *et al*., 2014) were grown and induced by IPTG.

### 4.6 Co‐immunoprecipitation

Co‐immunoprecipitation for identification of the interacting partner of HpaS was performed using the method previously described (Li *et al*., 2014). An Xcc strain expressing HpaS protein fused with 3 × Flag‐tag (HpaS::3 × Flag) at the C‐terminus of HpaS was first constructed. To do this, a DNA sequence encoding a 3 × Flag peptide at the C‐terminus of HpaS was obtained by PCR from the genomic DNA of Xcc wild‐type strain 8004 with the primer set *hpaS*‐FlagF/R (Table S4). This sequence was cloned into pLAFR3, and then the fused plasmid pHpaS‐Fla was introduced from *E. coli* JM109 by triparental conjugation into Δ*hpaS*, resulting in strain Δ*hpaS*/pHpaS‐Fla (Table S1).

The Δ*hpaS*/pHpaS‐Fla strain, and complemented strain CΔ*hpaS* expressing no. 3 × Flag‐tagged fusion protein as a negative control, were grown in the NYG medium overnight and collected by centrifugation at 4 °C for 10 min at 1,800 g, then washed in RIPA buffer (50 mM Tris‐HCl pH 7.4, 150 mM NaCl, 1 mM EDTA, 1% NP‐40, 0.5% sodium deoxycholate). The cells were lysed by resuspending them in 1 ml ice‐cold RIPA buffer containing protease inhibitor and resuspended cells were incubated on ice for 2 hr then centrifuged for 10 min at 4 °C, and the supernatant transferred into fresh tubes. To each sample was added 50 μl of anti‐FLAG (agarose‐conjugated) and the mixture was incubated with gentle shaking at 4 °C for 3 hr. Finally, the agarose was washed with ice‐cold RIPA buffer three times and the protein complexes eluted with 0.25 M glycine (pH 2.5). The eluted proteins were resolved by SDS‐PAGE and stained with Coomassie brilliant blue. Visible protein bands were excised from the gel, and the peptide sequences were deciphered by mass spectrometry (quadrupole time‐of‐flight).

### 4.7 Bacterial two‐hybrid assay

Bacterial two‐hybrid assays for detection of HpaS‐protein using the BacterioMatch II two‐hybrid system (Stratagene) were carried out as previously described (Li *et al*., 2014). The 1080‐bp truncated *hpaS* gene or 1011‐bp of the *fliM* gene obtained by PCR using primer set fliM‐BTF/R (Table S4) was cloned into the bait vector pBT, generating the plasmid pBT*hpaS*_LN54_ (Li *et al*., 2014) or pBT*fliM* (Table S1)*.* The coding sequence of *vemR* (or *cheY*) was PCR‐amplified from the Xcc strain using the primer set vemR‐TRGF/R (or cheY‐TRGF/R) (Table S4) and cloned into the target vector pTRG, resulting in plasmid pTRG*vemR* (or pTRG*cheY*) (Table S1). The plasmid pairs were co‐transformed into the reporter strain XL1‐Blue MRF′. The resulting strains were grown in liquid medium overnight, harvested, resuspended in M9 medium, and adjusted to a concentration of OD_600_ = 1.0 (for both nonselective and selective plates) and 0.1 (for selective plates only). Then 3 µl of bacterial suspension was spotted on the nonselective and double‐selection indicator plates containing 5 mM 3‐AT and 12.5 μg/ml Sm, and incubated at 28 °C for 24 hr.

### 4.8 Protein pull‐down assay

Protein pull‐down assay was performed as previously described (Li *et al*., 2014), with the ProFound pull‐down biotinylated protein–protein interaction kit (Pierce). Briefly, the *hpaS* fusion protein 6 × His::HpaS_LN54_ or FliM fusion protein FliM::6 × His was biotinylated with sulfo‐NHS‐LC‐biotin. Then 50 μl of the purified biotinylated 6 × His::HpaS_LN54_ (0.5 mg/ml) or FliM::6 × His was incubated with 40 μl of streptavidin sepharose beads. After washing, beads were incubated with 100 μl of sample containing 50 µg suspected prey protein 6 × His::VemR at 4 °C for at least 60 min, and then beads were washed and prey protein was eluted in 150 µl. Finally, 20 µl of the eluted sample was eletrophoresed on 12% SDS‐PAGE gel and visualized by Coomassie brilliant blue staining.

### 4.9 Analysis of VemR phosphorylation

The phosphorylation of response regulator VemR in vivo was analysed by the Phos‐tag SDS/PAGE method as previously described (Li *et al*., 2014). Briefly, Phos‐tag SDS/PAGE gels (Wako Pure Chemical Industries Ltd) were prepared according to the manufacturer's instructions. Xcc strains (Δ*vemR*/pHis*vemR*_lac_ and Δ*hpaS*Δ*vemR*/pHis*vemR*_lac_) expressing VemR with a 6 × His‐tag on its C‐terminus were cultivated in MMX medium, and total proteins from the bacterial cells were prepared. Ten (or 20) micrograms of total protein of each sample was loaded per well in a gel and electrophoresed. Simultaneously, samples were loaded onto SDS‐PAGE gel and electrophoresed. Gels were soaked in transfer buffer and then in chilled transfer buffer, followed by western blotting.

VemR in vitro phosphorylation was carried out using the purified 6 × His::VemR (or its derivate 6 × His::VemR_D11/56A_) and lithium potassium acetyl phosphate (AcP) (Sigma‐Aldrich). Proteins were incubated with 50 mM AcP at 37 °C for 30 min in a buffer containing 40 mM Tris‐HCl, pH 8.0, 10 mM MgCl_2_, 40 mM KCl, and 1 mM dithiothreitol. Samples were separated and detected on Phos‐tag SDS/PAGE gels.

### 4.10 Western blotting

Western blotting followed the procedure described previously (Sambrook *et al*., 1989). The proteins separated by Phos‐tag SDS‐PAGE (or SDS‐PAGE) gel were electrotransferred onto a polyvinylidene difluoride (PVDF) membrane (Millipore). After blocking with 5% bovine serum albumen, the protein was incubated with the 1:2,500 diluted anti‐His‐tag (or anti‐Flag‐tag) mouse monoclonal antibody (Qiagen) as a primary antibody, followed by four washes with Tris‐buffered saline with Tween (Tris 20 mM, NaCl 0.3 M, Tween 20 0.08% [vol/vol]) buffer. The diluted 1:2,500 horseradish peroxidase conjugated goat anti‐mouse IgG (Bio‐Rad) was used as secondary antibody. After washing the membrane four times, a luminescent signal was detected according to the manufacturer's instructions.

### 4.11 Transcriptome analysis

Transcriptome analysis of the HpaS mutant was performed as previously described (Cui *et al*., 2018). Briefly, Xcc strains were cultured in NYG medium to OD_600_ = 0.6, and RNA was prepared with an SV Total RNA Isolation System (Promega). Contaminating genomic DNA was removed using RNase‐free DNase I. After the quantity determination and quality assessment, total RNA was sent to Novogene (Beijing, China) for library construction and strand‐specific RNA sequencing. Sequencing libraries were generated using a NEB^Next^ Ultra Directional RNA Library Prep Kit for Illumina (New England BioLabs) and sequenced on an Illumina HiSeq 2000 platform. Clean reads were mapped to the reference genome and the reads per kilobase per million mapped reads (RPKM) method was used to calculate the gene expression levels. False discovery rate (FDR) ≤ 0.05 and |log_2_FC| (log_2_ of the fold changes) ≥1 were considered for differentially expressed genes. For confirmation, several differentially expressed genes (DEGs) were selected randomly to perform semiquantitative RT‐PCR analysis (Table S3).

### 4.12 Extracellular polysaccharide and extracellular enzymes assays

EPS and extracellular enzyme assays were performed as previously described (Tang *et al*., 1991; Su *et al*., 2016). To estimate EPS production, Xcc strains were spotted onto NYG agar plates supplemented with 2% glucose and grown for 5 days. For EPS yield measurement, Xcc strains were inoculated into 100 ml NY liquid medium containing glucose (2% wt/vol) at 28 °C, 200 rpm for 3 days. EPS was precipitated from the culture supernatant with ethanol, dried, and weighed. For quantitative measurement of the activity of extracellular enzymes, Xcc strains were cultured in NYG medium, and the activities of protease, endoglucanase (cellulase), and amylase were measured as previously described (Su *et al*., 2016).

### 4.13 Cell motility assays

Cell motility was tested as previously described (Su *et al*., 2016). To detect swimming motility, an overnight culture (OD_600_ = 1.0) of each Xcc strain was stabbed into 0.28% agar plates composed of 0.03% Bacto peptone and 0.03% yeast extract followed by incubation at 28 °C for 4 days. To test swarming motility, the bacterial cells were inoculated onto NY plates containing 2% glucose and 0.6% agar using a toothpick, then incubated at 28 °C for 3 days. The diameter of the area occupied by strains was measured and the values were used to indicate the motility of Xcc strains. The experiment was repeated three times.

### 4.14 Stress tolerance assay

The well‐established and widely used minimal inhibitory concentration (MIC) method (Wiegand *et al*., 2008) was employed to test the resistance of the Xcc strains to several environmental stresses, including SDS, the organic solvent phenol, hyperosmotic challenge NaCl, and heavy metal salt CdCl_2_. Briefly, Xcc strains were cultured to OD_600_ = 0.6 and diluted, then 100 μl of the diluted culture was plated on NYG plates supplemented with different concentrations of each reagent. The surviving colonies on the plates were counted after 3 days of incubation at 28 °C.

## Data Availability

The data that support the findings of this study are available from the corresponding author upon reasonable request.
