# Supplementary figures and images for: Xanthomonas campestris sensor kinase HpaS co‐opts the orphan response regulator VemR to form a branched two‐component system that regulates motility
Source: Mol Plant Pathol. 2020 Jan 9;21(3):360–75. doi: 10.1111/mpp.12901 (PMC7036368; doi:10.1111/mpp.12901)

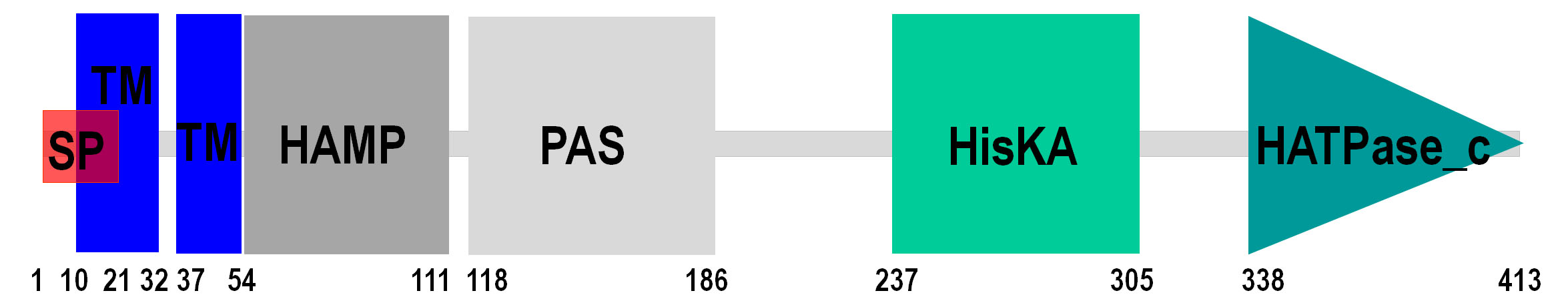

Supplement: Supplementary file 1 [file MPP-21-360-s001.jpg]

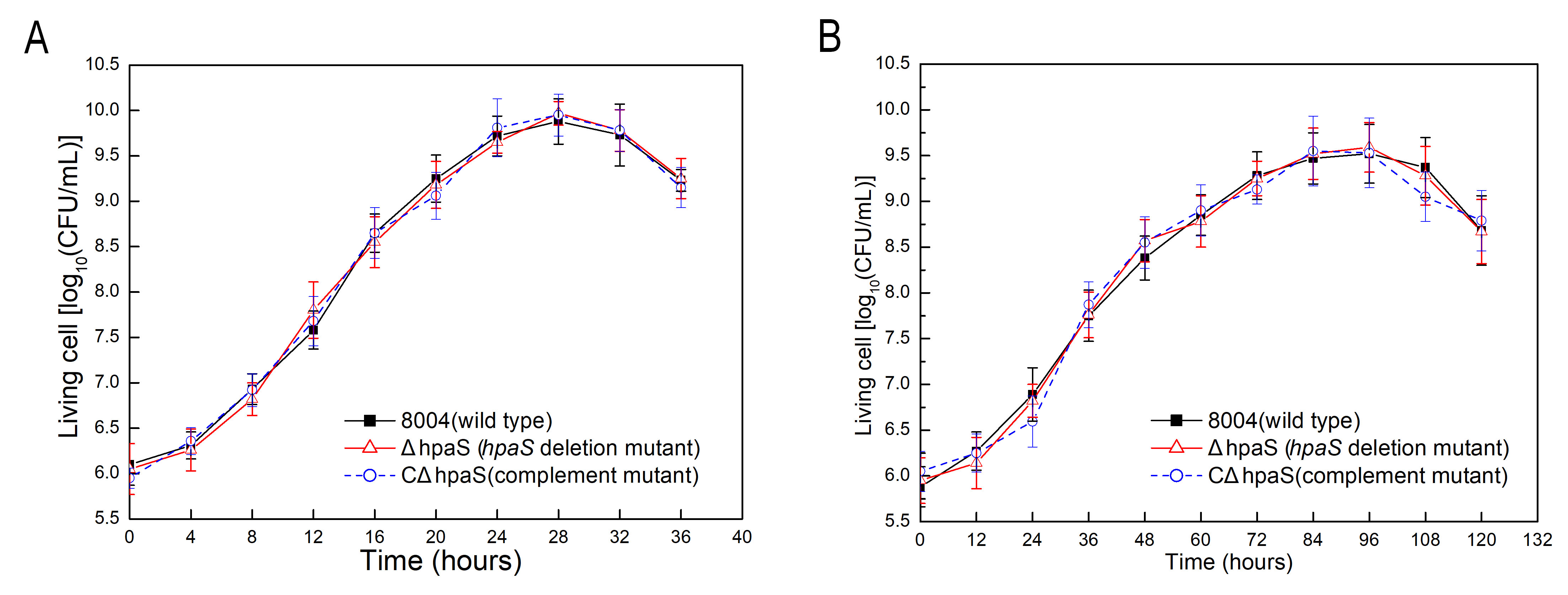

Supplement: Supplementary file 2 [file MPP-21-360-s002.jpg]

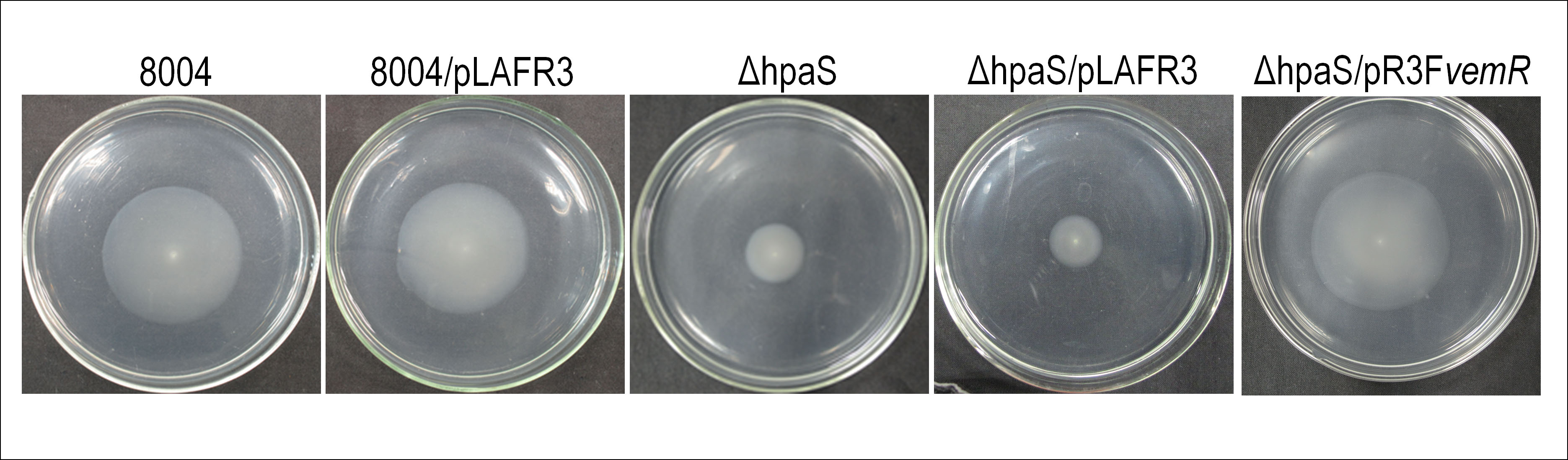

Supplement: Supplementary file 3 [file MPP-21-360-s003.jpg]

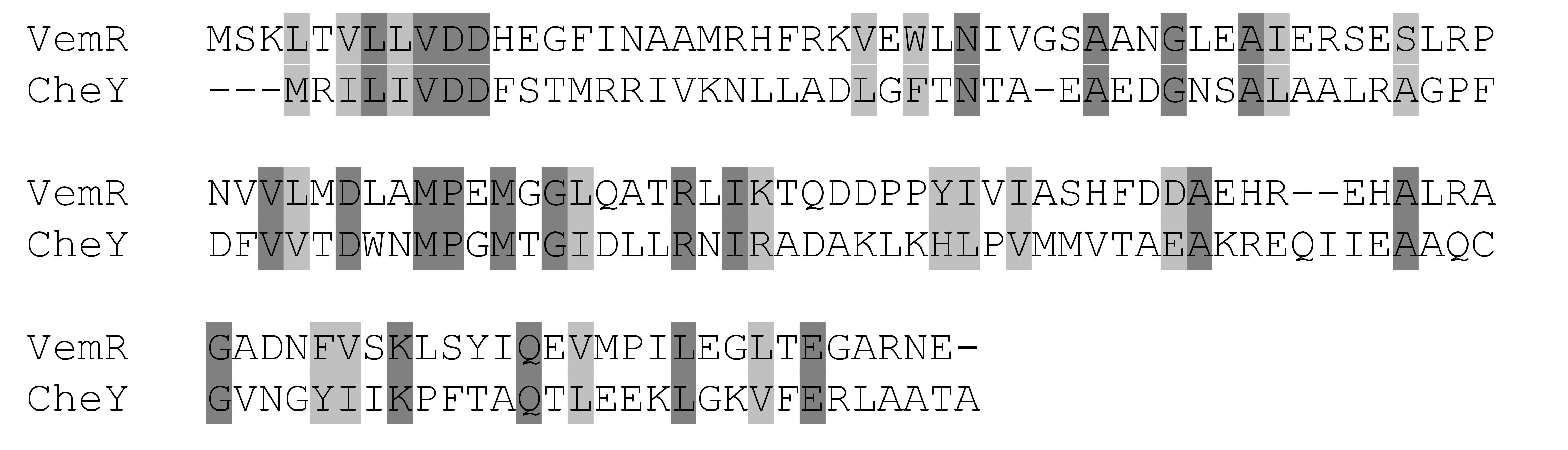

Supplement: Supplementary file 4 [file MPP-21-360-s004.jpg]
