# Supplementary material for: Xanthomonas campestris sensor kinase HpaS co‐opts the orphan response regulator VemR to form a branched two‐component system that regulates motility
Source: Mol Plant Pathol. 2020 Jan 9;21(3):360–75. doi: 10.1111/mpp.12901 (PMC7036368; doi:10.1111/mpp.12901)
Supplement: Supplementary file 5 [file MPP-21-360-s005.doc]

**Table S1. Strains and plasmids used in this study**

| Strains or plasmids | Relevant characteristics | Reference or source |
| --- | --- | --- |
| *E. coli* strains |  |  |
| JM109 | *RecA*1*, endA*1*, gyrA*96*, thi, supE*44*, relA*1  △ (*lac-proAB*)/F’ [*traD36, lacI*^q^, *lacZ* △M15] | Yanisch-Perron *et al*., 1985 |
| DH5α | Φ80△*lacZM*15 *recA1 endA1 deoR* | Gibco BRL, Life Technologies |
| M15 | *lac ara gal mtl recA1 uvr1* [pREP4 *lacI* Kan^r^] | Qiagen |
| BL21(DE3) | F^-^ *ompT gal dcm lon hsdS_B_* (*r^-^_B_ m^-^_B_*) λ(DE3) | Novagen, Germany |
| M15/pQE-30-VemR | M15 harboring plasmid pQE-30-VemR | This work |
| M15/pQE-30-VemR_D11/56A_ | M15 harboring plasmid pQE-30-VemR_D11/56A_. | This work |
| M15/pQE-HpaS_LN54_ | M15 harbouring pQE-HpaS_LN54_ | Li *et al*., 2014 |
| M15/pQE-RavR | M15 harbouring pQE-RavR | Li *et al*., 2014 |
| BL21/pET-32a-FliM | BL21(DE3) harboring plasmid pET-30a-FliM | This work |
| BL21/pET-30a-CheY | BL21(DE3) harboring plasmid pET-30a-CheY | This work |
| XL1-Blue MRF' | Reporter strain, Δ*(mcrA)183* Δ*(mcrCB-hsdSMR-mrr)173 endA1 hisB supE44 thi-1 recA1 gyrA96relA1 lac* [F*´ lacIq HIS3 aadA* Kan*^r^*] | Stratagene |
| XL1-Blue MRF'/pBT*hpaS_LN54_*/pTRG*hrpG* | XL1-Blue MRF' harboring plasmids pBT*hpaS_LN54_* and pTRG*hrpG* | Li *et al*., 2014 |
| XL1-Blue MRF'/pBT*hpaS_LN54_*/pTRG*vemR* | XL1-Blue MRF' harboring plasmids pBT*hpaS_LN54_* and pTRG*vemR* | This work |
| XL1-Blue MRF'/pBT*hpaS_LN54_*/pTRG | XL1-Blue MRF' harboring plasmids pBT*hpaS_LN54_* and pTRG | This work |
| XL1-Blue MRF'/pBT/pTRG*vemR* | XL1-Blue MRF' harboring plasmids pBT and pTRG*vemR* | This work |
| XL1-Blue MRF'/pBT/pTRG | XL1-Blue MRF' harboring plasmids pBT and pTRG | This work |
| XL1-Blue MRF'/pBT*fliM*/pTRG*vemR* | XL1-Blue MRF' harboring plasmids pBT*fliM* and pTRG*vemR* | This work |
| XL1-Blue MRF'/pBT*fliM*/pTRG*cheY* | XL1-Blue MRF' harboring plasmids pBT*fliM* and pTRG*cheY* | This work |
| XL1-Blue MRF'/pBT/pTRG*cheY* | XL1-Blue MRF' harboring plasmids pBT and pTRG*cheY* | This work |
| XL1-Blue MRF'/pBT/pRGT | XL1-Blue MRF' harboring plasmids pBT and pRGT | Li *et al*., 2014 |
| XL1-Blue MRF'/pBT*hpaS*_LN54_/pTRG | XL1-Blue MRF' harboring plasmids pBT*hpaS*_LN54_ and pTRG | Li *et al*., 2014 |
| *Xanthomonas campestris* pv. *campestris* strains |  |  |
| 8004 | Wild-type strain. Rif^r^ | Daniels *et al*., 1984 |
| 8004/pLAFR3 | 8004 harboring an empty vector pLAFR3. Rif^r^ Tet^r^ | This work |
| ΔhpaS | As 8004, but *hpaS* gene (*XC_3670*) deleted. Rif^r^ Kan^r^ | Li *et al*., 2014 |
| C∆hpaS | ΔhpaS harboring a recombinant plasmid derived from the full length of *hpaS* cloned into the promoterless plasmid pLAFR6. Rif^r^ Kan^r^ Tet^r^ | Li *et al*., 2014 |
| ∆hpaS/pLAFR3 | ΔhpaS harboring an empty vector pLAFR3. Rif^r^ Kan^r^ Tet^r^ | This work |
| ΔhpaS/pR3F*vemR* | ∆hpaS harboring the recombinant plasmid pR3F*vemR*, Rif^r^ Kan^r^ Tet^r^ | This work |
| ΔvemR | As 8004, but *vemR* gene (*XC_2252*) deleted. Rif^r^ | This work |
| ΔvemR/pHis*vemR_lac_* | ∆vemR harboring the recombinant plasmid pHis*vemR*_lac_, Rif^r^ Tet^r^ | This work |
| ΔhpaSΔvemR | As 8004, but *hpaS* and *vemR* genes deleted. Rif^r^ Gm^r^ | This work |
| ΔhpaSΔvemR/pHis*vemR_la_*_c_ | ΔhpaS∆vemR harboring the recombinant plasmid pHis*vemR*_lac_, Rif^r^ Gm^r^ Tet^r^ | This work |
| ΔhpaSΔvemR/pR3O*vemR* | ΔhpaSΔvemR harboring the recombinant plasmid pR3O*vemR*, Rif^r^ Gm^r^ Tet^r^ | This work |
| ΔhpaS/pHpaS-Fla | ∆hpaS harboring the recombinant plasmid pHpaS-Fla, Rif^r^ Kan^r^ Tet^r^ | This work |
| ΔfliM | As 8004, but *fliM* gene (*XC_2267*) deleted. Rif^r^ | This work |
| CΔfliM | ΔfliM harboring a recombinant plasmid pR3O*fliM* derived from the ORF of *fliM* cloned into the plasmid pLAFR3. Rif^r^ Tet^r^ | This work |
| ΔcheY | As 8004, but *cheY* gene (*XC_2282*) deleted. Rif^r^ | This work |
| CΔcheY | ΔcheY harboring a recombinant plasmid pR3O*cheY* derived from the ORF of *cheY* cloned into the plasmid pLAFR3. Rif^r^ Tet^r^ | This work |
| Plasmids |  |  |
| pLAFR3 | Broad host range cloning vector, Tet^r^ | Staskawicz *et al*., 1987 |
| pRK2073 | Helper plasmid, Tra+, Mob+, ColE1, Spc^r^. | Leong *et al*., 1982 |
| pK18*mobsacB* | pUC18 derivative, *lacZα*, *sacB*, Kan^r^, *mob* site. Allelic exchange vector (Suicidal vector carrying *sacB* gene for mutagenesis) | Schäfer *et al*., 1994 |
| pK18*mob* | pUC18 derivative, *lacZα* Kan^r^, *mob* site. Suicide plasmid in *X. campestris* pv. *campestris*. | Schäfer *et al*. 1994 |
| pK18*mobsacBvemR* | pK18*mobsacB* containing fragments flanking *vemR*. Kan^r^ | This work |
| pK18*mobsacBhpaS* | pK18*mobsacB* containing fragments flanking *hpaS* and Gm-resistant fragment. Kan^r^ Gm ^r^ | Li *et al*., 2014 |
| pR3F*vemR* | 434-bp DNA fragment of the *vemR* gene (including 50 nucleotides upstream of the start codon) of *Xcc* strain cloned into the plasmid pLAFR3. Tet^r^ | This work |
| pR3O*vemR* | 381-bp DNA fragment of the *vemR* gene coding sequence (*XC_2252*) of *Xcc* strain cloned into the plasmid pLAFR3. Tet^r^ | This work |
| pR3O*fliM* | 1101-bp DNA fragment of the *fliM* gene coding sequence (*XC_2267*) of *Xcc* strain cloned into the plasmid pLAFR3. Tet^r^ | This work |
| pR3O*cheY* | 378-bp DNA fragment of the *fliM* gene coding sequence (*XC_2282*) of *Xcc* strain cloned into the plasmid pLAFR3. Tet^r^ | This work |
| pHis*vemR_lac_* | pLAFR3 containing the encoding sequence of VemR fused with 6×His-tag at the C-terminus of VemR, Tet^r^ | This work |
| pK*vemR* | The suicide plasmid pK18*mob* containing 381-bp *vemR* (*XC_2252*) coding sequence of *Xcc* strain. Used for site-directed mutagenesis. Kan^r^ | This work |
| pK*vemR_D11/56A_* | The suicide plasmid pK18*mob* containing a point-mutated *vemR* gene (aspartate at position 11 and 56 was substituted with alanine). Kan^r^ |  |
| pQE-30 | Expression vector, allowing the production of fusion proteins containing amino terminal 6×His-tagged sequences. Ampr | Qiagen, Germany |
| pQE-30-VemR | pQE-30 containing a 381-bp fragment of *vemR* gene coding region | This work |
| pQE-30-VemR_D11/56A_ | pQE-30 containing a 381-bp fragment of point-mutated *vemR* gene ( replacing aspartate at position 11 and 56 to alanine) | This work |
| pQE-30 Xa | Expression vector, allow the production of fusion proteins containing amino terminal 6xHis-tagged sequences. Ampr | Qiagen, Germany |
| pQE-HpaS_LN54_ | pQE-30 Xa containing a 1080-bp fragment of *hpaS* gene coding region lacking the nt 1 to 162. | Li et al., 2014 |
| pQE-RavR | pQE-30 Xa containing a 1659-bp fragment of *ravR* gene coding region lacking the nt 1 to 63. | Li *et al*., 2014 |
| pET-30a | Expression vector, allow the production of fusion proteins containing amino terminal 6×His-tagged sequences. Kanr | Novagen |
| pET-32a | Expression vector, allow the production of fusion proteins containing amino terminal thioredoxin-tagged and carboxyl-terminal 6×His-tagged sequences. Amp^r^ | Novagen |
| pET-32a-FliM | pET-30a containing a 1011-bp fragment of *fliM* gene coding region | This work |
| pET-30a-CheY | pET-30a containing a 378-bp fragment of *cheY* gene coding region | This work |
| pHpaS-Fla | pLAFR3 containing the encoding sequence of HpaS fused with 3×Flag-tag at the C-terminus of HpaS, Tet^r^ | This work |
| pBT | Two-hybrid system bait plasmid containing the *cat* gene, p15A origin of replication and λ cI ORF. | Stratagene |
| pBT*hpaS_LN54_* | pBT derivative carrying the 1080-bp of *hpaS* gene lacking the 1^th^ to 162^th^ nucleotides. Cat^r^ | Li *et al*., 2014 |
| pBT*fliM* | pBT derivative carrying a 1011-bp fragment of *fliM* gene coding region. Cat^r^ | This work |
| pTRG | Two-hybrid system target plasmid containing the *tet* gene, ColE1 origin of replication, and RNA polymerase α subunit ORF. | Stratagene |
| pTRG*vemR* | pTRG derivative carrying 381-bp fragment of *vemR* gene coding region. Tet^r^ | This work |
| pTRG*cheY* | pTRG derivative carrying 378-bp fragment of *cheY* gene coding region. Tet^r^ | This work |
| pTRG*hrpG* | pTRG derivative carrying the full length of the coding region of *hrpG* gene (789-bp). Tet^r^ | Li *et al*., 2014 |

**References**

Daniels, M.J., Barber, C.E., Turner, P.C., Sawczyc, M.K., Byrde, R.J.W., and Fielding, A.H. (1984) Cloning of genes involved in pathogenicity of *Xanthomonas campestris* pv. *campestris* using the broad host range cosmid pLAFR1. *EMBO J* **3:** 3323–3328.

Leong, S.A., Ditta, G.S., and Helinski, D.R. (1982) Heme biosynthesis in *Rhizobium*. Identification of a cloned gene coding for delta-aminolevulinic acid synthetase from *Rhizobium meliloti*. *J Biol Chem* **257:** 8724–8730.

Li, R.F., Lu, G.T., Li, L., Su, H.Z., Feng, G.F., Chen, Y., *et al*. (2014) Identification of a putative cognate sensor kinase for the two-component response regulator HrpG, a key regulator controlling the expression of the *hrp* genes in *Xanthomonas campestris* pv. *campestris*. *Environ Microbiol* **16**: 2053–2071.

Schäfer, A., Tauch, A., Jäger, W., Kalinowski, J., Thierbach, G., and Pühler, A. (1994) Small mobilizable multi-purpose cloning vectors derived from the *Escherichia coli* plasmids pK18 and pK19: selection of defined deletions in the chromosome of *Corynebacterium glutamicum*. *Gene***145:** 69–73.

Staskawicz, B., Dahlbeck, D., Keen, N., and Napoli, C. (1987) Molecular characterization of cloned avirulence genes fromrace 0 and race 1 of *Pseudomonas syringae* pv. *glycinea*. *J Bacteriol* **169:** 5789–5794.

Yanisch-Perron, C., Vieira, J., and Messing, J. (1985) Improved M13 phage cloning vectors and host strains: nucleotide sequences of the M13mp18 and pUC19 vectors. *Gene* **33**: 103–119.
