# Supplementary material for: Xanthomonas campestris sensor kinase HpaS co‐opts the orphan response regulator VemR to form a branched two‐component system that regulates motility
Source: Mol Plant Pathol. 2020 Jan 9;21(3):360–75. doi: 10.1111/mpp.12901 (PMC7036368; doi:10.1111/mpp.12901)
Supplement: Supplementary file 6 [file MPP-21-360-s006.doc]

**Table S2. Gene expression profile of the *hpaS* mutant strain ∆hpaS when grown in NYG medium.**

| Function Category | Gene ID | Name | Annotation | ∆hpaS fold change |
| --- | --- | --- | --- | --- |
| Amino acids biosynthesis | *XC_0530* | *trpA* | tryptophan synthase alpha chain | 2.17 |
|  | *XC_0548* | *aroG* | phospho-2-dehydro-3-deoxyheptonate aldolase, phe-sensitive | 2.31 |
|  | *XC_2375* | *hisF* | bifunctionalaspartokinase/homoserine dehydrogenase I | 3.36 |
|  | *XC_2377* | *hisH* | amidotransferase | 3.99 |
|  | *XC_2379* | *hisC* | histidinol-phosphate aminotransferase | 3.09 |
|  | *XC_2848* | *asnB* | amino-acid acetyltransferase | 3.59 |
| Biosynthesis of cofactors, prosthetic groups, carriers | *XC_0574* | *mdcB* | beta subunit of malonate decarboxylase | 2.67 |
|  | *XC_0575* | *mdcC* | malonate decarboxylase gamma subunit | 2.33 |
|  | *XC_0576* | *mdcE* | malonate decarboxylase | 2.33 |
|  | *XC_0577* | *citG* | CitG protein | 2.21 |
|  | *XC_1335* | *apbE* | thiamine biosynthesis lipoprotein ApbE precursor | 4.37 |
|  | *XC_2549* | *pqqC/D* | PqqC/D protein | 5.05 |
|  | *XC_2555* |  | methyltransferase | 2.4 |
|  | *XC_0400* | *bioB* | biotin synthase | -2.03 |
|  | *XC_3157* |  | hydroxylase molybdopterin-containing subunit | -5.38 |
| Cell envelope and cell structure | *XC_0232* | *ddlB* | pre-pilin like leader sequence | 3.26 |
|  | *XC_0811* | *oprO* | porin O | 4.05 |
|  | *XC_1434* | *oprM* | outer membrane protein | 5.58 |
|  | *XC_1459* | *phuR* | outer membrane hemin receptor | 2.79 |
|  | *XC_1529* | *oprO* | polyphosphate-selective porin O | 2.47 |
|  | *XC_3129* | *yjdB* | inner membrane protein | 3.69 |
|  | *XC_3445* | *lgt* | prolipoprotein diacylglyceryl transferase | 2.41 |
|  | *XC_3785* |  | ferrichrome-iron receptor 3 | 3.03 |
|  | *XC_4094* | *actII-3* | membrane protein | 2.88 |
|  | *XC_4095* |  | membrane protein | 2.31 |
|  | *XC_4139* |  | ankyrin-like protein | 2.01 |
|  | *XC_0970* | *ompW* | outer membrane protein | -2.5 |
|  | *XC_1545* |  | Oar protein | -2.81 |
|  | *XC_1621* | *fimT* | pre-pilin like leader sequence | -2.02 |
|  | *XC_1622* | *pilV* | pre-pilin leader sequence | -2.04 |
|  | *XC_1624* | *pilX* | PilX protein | -1.85 |
|  | *XC_1626* | *pilE1* | type IV pilin | -2.18 |
|  | *XC_2151* |  | L-sorbosone dehydrogenase | -6.31 |
|  | *XC_2171* | *rfbD* | strX protein | -6.68 |
|  | *XC_3312* |  | membrane protein | -2.1 |
|  | *XC_3769* | *rfbD* | UDP-galactopyranose mutase | -2.5 |
| Cellular processes | *XC_0286* | *tsr* | chemotaxis protein | 4.28 |
|  | *XC_0336* | *tsr* | chemotaxis protein | 3.49 |
|  | *XC_0638* | *tsr* | chemotaxis protein | 3.14 |
|  | *XC_0805* |  | amylosucrase or alpha amylase | 2.71 |
|  | *XC_1410* | *cheR* | response regulator for chemotaxis | 5.61 |
|  | *XC_1412* | *cheW* | chemotaxis protein | 3.19 |
|  | *XC_1413* | *mcp* | chemotaxis protein | 2.61 |
|  | *XC_1414* | *cheA* | chemotaxis protein | 3.3 |
|  | *XC_1801* | *mcp* | chemotaxis protein | 4.05 |
|  | *XC_1802* | *cheW* | chemotaxis protein | 2.87 |
|  | *XC_2136* | *tsr* | chemotaxis protein | 3.16 |
|  | *XC_2223* | *mcp* | chemotaxis protein | 3.05 |
|  | *XC_2231* | *flgM* | flagellar protein | +2.22 |
|  | *XC_2233* | *cheV* | chemotaxis protein | 2.47 |
|  | *XC_2297* | *motA* | MotA protein | 4.26 |
|  | *XC_2299* | *parA* | chromosome partioning protein | 2.04 |
|  | *XC_2300* | *cheW* | chemotaxis protein | 2.17 |
|  | *XC_2302* | *cheY* | chemotaxis response regulator | 3.3 |
|  | *XC_2303* | *cheA* | chemotaxis protein | 2.53 |
|  | *XC_2304* | *tsr* | chemotaxis protein | 2.65 |
|  | *XC_2308* | *tsr* | chemotaxis protein | 4.05 |
|  | *XC_2309* | *tsr* | chemotaxis protein | 5.97 |
|  | *XC_2318* | *cheW* | chemotaxis protein | 4.43 |
|  | *XC_2320* | *tsr* | chemotaxis protein | 4.12 |
|  | *XC_2321* | *cheR* | chemotaxis protein methyltransferase | 6.59 |
|  | *XC_2472* | *amiC* | N-acetylmuramoyl-L-alanine amidase | 4.49 |
|  | *XC_2504* | *mcpA* | chemotaxis protein | 3.69 |
|  | *XC_3724* | *motA* | chemotaxis protein | 2.62 |
|  | *XC_0142* |  | trehalose synthase | -9.77 |
|  | *XC_0143* | *glgB1* | 1,4-alpha-glucan branching enzyme | -3.91 |
|  | *XC_0421* | *glgA* | glycogen synthase | -4.73 |
|  | *XC_0422* | *glgB2* | 1,4-alpha-glucan branching enzyme | -5.05 |
|  | *XC_0424* |  | 4-alpha-glucanotransferase | -7.4 |
|  | *XC_1290* | *cheB* | protein-glutamate methylesterase (CheB) | -3.85 |
|  | *XC_1368* | *osmC* | osmotically inducible protein | -5.26 |
|  | *XC_2163* | *cheB* | protein-glutamate methylesterase | -15.62 |
|  | *XC_2234* | *flgB* | flagellar protein | -2.64 |
|  | *XC_2235* | *flgC* | flagellar biosynthesis, cell-proximal portion of basal-body rod | -2.07 |
|  | *XC_2245* | *fliC* | flagellar protein | -2.31 |
|  | *XC_2247* | *fliS* | flagellar protein | -2.65 |
|  | *XC_2259* | *fliE* | flagellar protein | -2.58 |
|  | *XC_2266* | *fliL* | flagellar biosynthesis protein | -2.24 |
|  | *XC_3415* | *gfo* | glucose-fructose oxidoreductase | -3.64 |
| Central intermediary metabolism | *XC_0152* | *estA1* | carboxylesterase type B | 2.99 |
|  | *XC_0154* | *mhpD* | 2-keto-4-pentenoate hydratase | 3.62 |
|  | *XC_1334* |  | sulfite reductase | 4.46 |
|  | *XC_1881* | *gaa* | glutaryl-7-ACA acylase precursor | 2.41 |
|  | *XC_2325* | *pcaD* | beta-ketoadipate enol-lactone hydrolase | 2.82 |
|  | *XC_2478* |  | D-xylulokinase | 2.19 |
|  | *XC_2480* | *xylS* | alpha-xylosidase | 4.8 |
|  | *XC_2482* |  | sialic acid-specific 9-O-acetylesterase | 3.58 |
|  | *XC_2487* | *dgoA* | vanillate O-demethylase oxygenase subunit | 22.9 |
|  | *XC_2488* | *dgoA* | 4-hydroxy-2-oxoglutarate aldolase/2-deydro-3-deoxyphosphogluconate aldolase | 17.95 |
|  | *XC_2490* | *dgoK* | 2-oxo-3-deoxygalactonate kinase | 3.27 |
|  | *XC_2984* | *galA* | arabinogalactan endo-1,4-beta-galactosidase | 2.23 |
|  | *XC_2985* | *bga* | beta-galactosidase | 2.36 |
|  | *XC_3491* | *dgd* | D-galactose 1-dehydrogenase | 2.33 |
|  | *XC_4119* | *fldZ* | acyl transferase | 2.24 |
|  | *XC_4315* | *glxK* | glycerate kinase | 2.47 |
|  | *XC_4326* | *phoC* | phosphatase precursor | 2.06 |
|  | *XC_0150* |  | L-fucose dehydrogenase | -8.04 |
|  | *XC_0370* | *glpK* | glycerol kinase | -3.71 |
|  | *XC_0372* | *glpD* | glycerol-3-phosphate dehydrogenase | -3.13 |
|  | *XC_0374* | *pobB* | phenoxybenzoate dioxygenase beta subunit | -4.33 |
|  | *XC_0375* | *vanA* | vanillate O-demethylase oxygenase subunit | -2.79 |
|  | *XC_0423* |  | maltooligosyltrehalose trehalohydrolase | -6.89 |
|  | *XC_0425* | *glgY* | maltooligosyltrehalose synthase | -5.99 |
|  | *XC_0427* | *glgX* | glycogen debranching enzyme | -3.82 |
|  | *XC_0853* |  | H+ translocating pyrophosphate synthase | -4.22 |
|  | *XC_1002* | *susB* | alpha-glucosidase | -2.27 |
|  | *XC_1047* | *glgX* | glycogen debranching enzyme | -3.87 |
|  | *XC_1780* | *gabD* | succinate-semialdehyde dehydrogenase | -2.77 |
|  | *XC_2458* |  | mannan endo-1,4-beta-mannosidase | -4.12 |
|  | *XC_2981* | *mmsA* | methylmalonate-semialdehyde dehydrogenase | -3.35 |
|  | *XC_3054* | *lamA* | endo-1,3-beta-glucanase precursor | -2.28 |
|  | *XC_3767* |  | UDP-glucose 4-epimerase | -12.86 |
| Energy and carbon metabolism | *XC_0686* | *adhC* | alcohol dehydrogenase class III | 2.29 |
|  | *XC_1436* |  | oxidoreductase | 4.39 |
|  | *XC_2562* |  | cytochrome like B561 | 4.2 |
|  | *XC_2573* | *dsbE* | C-type cytochrome biogenesis protein/thioredoxin | 3.9 |
|  | *XC_2800* | *dauE* | aklaviketone re | 2.41 |
|  | *XC_3488* |  | phosphoglycerate mutase | 2.94 |
|  | *XC_4120* | *fldA* | FldA protein | 3.11 |
|  | *XC_0216* | *dsbE* | C-type cytochrome biogenesis protein/thioredoxin | -2.68 |
|  | *XC_0979* |  | fructose-bisphosphate aldolase | -2.22 |
|  | *XC_1057* | *gcd* | glucose dehydrogenase | -2.27 |
|  | *XC_1166* | *glk* | glucose kinase | -2.47 |
|  | *XC_1300* |  | quinol oxidase, subunit I | -6.36 |
|  | *XC_1301* | *qxtB* | quinol oxidase, subunit II | -4.55 |
|  | *XC_1384* |  | alcohol dehydrogenase | -2.72 |
|  | *XC_1385* | *yagT* | oxidoreductase | -3.24 |
|  | *XC_1386* | *yagS* | oxidoreductase | -3.2 |
|  | *XC_1387* | *yagR* | oxidoreductase | 3.74 |
|  | *XC_1452* |  | formate dehydrogenase related protein | -12.21 |
|  | *XC_1455* | *qxtB* | cytochrome D oxidase subunit B | -3.03 |
|  | *XC_1793* | *petB* | ubiquinol cytochrome C oxidoreductase, cytochrome B subunit | -2.45 |
|  | *XC_1794* | *petC* | ubiquinol cytochrome C oxidoreductase, cytochrome C1 subunit | -2.59 |
|  | *XC_1885* | *cydA* | cytochrome D ubiquinol oxidase subunit I | -3.04 |
|  | *XC_2188* | *fdh* | glutathione-dependent formaldehyde dehydrogenase | -33.78 |
|  | *XC_2192* | *yxnA* | glucose 1-dehydrogenase homolog | -11.08 |
|  | *XC_2585* |  | dehydrogenase | -5.98 |
|  | *XC_2659* | *gcd* | glucose dehydrogenase | -5.98 |
|  | *XC_2742* | *fumC* | fumarate hydratase | -3.4 |
|  | *XC_2751* | *ldp* | dihydrolipoamide dehydrogenase | -2.06 |
|  | *XC_3081* | *cyoD* | cytochrome O ubiquinol oxidase subunit IV | -2.14 |
|  | *XC_3083* | *cyoB* | cytochrome O ubiquinol oxidase subunit I | -2.22 |
|  | *XC_3156* |  | ferredoxin | -3.78 |
|  | *XC_3167* |  | oxidoreductase | -6.85 |
|  | *XC_3170* |  | oxidoreductase | -12.64 |
|  | *XC_3740* |  | oxidoreductase | -3.38 |
|  | *XC_3762* | *cioA* | cyanide insensitive terminal oxidase | -5.82 |
|  | *XC_3763* | *cioB* | cyanide insensitive terminal oxidase | -5.31 |
|  | *XC_3766* |  | oxidoreductase | -13.6 |
|  | *XC_3774* |  | Zn-dependent alcohol dehydrogenase | -2.85 |
|  | *XC_3901* | *cox3* | cytochrome C oxidase subunit III | -5.09 |
|  | *XC_3902* | *cioA* | cytochrome C oxidase assembly protein ctaG | -3.83 |
|  | *XC_3904* | *ctaD* | cytochrome C oxidase subunit I | -4.29 |
|  | *XC_3905* | *ctaC* | cytochrome C oxidase subunit II | -3.38 |
|  | *XC_4082* | *zwf* | glucose-6-phosphate 1-dehydrogenase | -5.03 |
| Fatty acid and phospholipid metabolism | *XC_0186* | *cls* | cardiolipin synthase | 2.25 |
|  | *XC_0276* |  | lipase | 2.6 |
|  | *XC_0387* |  | lipase | 2.59 |
|  | *XC_0573* | *mdcD* | delta subunit of malonate decarboxylase | 2.44 |
|  | *XC_0809* | *phbB* | acetoacetyl-coA reductase | 2.51 |
|  | *XC_3288* |  | non-hemolytic phospholipase C | 2.45 |
|  | *XC_3424* |  | acid-CoA ligase | 2.44 |
|  | *XC_4092* | *fabG* | 3-oxoacyl-[ACP] reductase | 2.37 |
|  | *XC_1394* |  | phospholipase A1 | -3.34 |
|  | *XC_1395* | *plaS* | accessory protein | -5.31 |
| Regulatory functions | *XC_0246* | *rbcR* | transcriptional regulator | 2.11 |
|  | *XC_0506* |  | transcriptional regulator lysR family | 2.53 |
|  | *XC_0601* |  | transcriptional regulator marR family | 4.04 |
|  | *XC_0773* | *ybhD* | transcriptional regulator | 2.43 |
|  | *XC_0778* |  | transcriptional regulator | 2.58 |
|  | *XC_1435* |  | transcriptional regulator | 6.84 |
|  | *XC_1766* | *rrpX* | transcriptional regulator | 3.2 |
|  | *XC_2510* | *lexA* | LexA | 3.31 |
|  | *XC_2729* |  | transcriptional regulator | 2.89 |
|  | *XC_2840* | *yybA* | transcriptional regulator marR family | 3.21 |
|  | *XC_3216* | *prpR* | propionate catabolism regulatory protein | 2.71 |
|  | *XC_3386* |  | transcriptional regulator | 3.49 |
|  | *XC_3419* |  | transcriptional regulator marR family | 3.78 |
|  | *XC_4021* |  | transcriptional regulator | 2.18 |
|  | *XC_4118* | *fldY* | transcriptional regulator | 2.79 |
|  | *XC_4254* | *slyA* | transcriptional regulator for cryptic hemolysin | 7.07 |
|  | *XC_0325* | *cmfA* | conditioned medium factor | -3.65 |
|  | *XC_2650* | *phaF* | poly(hydroxyalcanoate) granule associated protein | -3.56 |
|  | *XC_3758* | *exsF* | regulatory protein | -6.14 |
|  | *XC_3760* |  | transcriptional regulator NtrC family | -2.05 |
| Replication and DNA metabolism | *XC_1226* | *nudE* | ADP compounds hydrolase | 2.09 |
|  | *XC_1684* |  | excinuclease ABC subunit C homolog | 4.84 |
|  | *XC_2509* | *recA* | RecA protein | 2.03 |
|  | *XC_2612* |  | RadC family protein | 11.39 |
|  | *XC_2785* | *lig3* | ATP-dependent DNA ligase | 4.22 |
|  | *XC_3148* |  | ATP-dependent DNA ligase | 2.42 |
|  | *XC_4239* | *mutM* | formamidopyrimidine DNA glycosylase | 2.38 |
|  | *XC_0633* |  | endonuclease | -3.25 |
|  | *XC_1403* | *nfi* | endonuclease V | -2.42 |
|  | *XC_2186* | *xthA2* | exodeoxyribonuclease III | -2.99 |
| Transport | *XC_0084* | *proP* | proline/betaine transporter | 9.28 |
|  | *XC_0124* | *iroN* | TonB-dependent receptor | 3.71 |
|  | *XC_0155* | *dctP* | C4-dicarboxylate transport system | 2.8 |
|  | *XC_0157* | *ygiK* | C4-dicarboxylate transport protein | 3.24 |
|  | *XC_0167* | *fpvA* | ferripyoverdine receptor | 3.87 |
|  | *XC_0434* | *mexE* | component of multidrug efflux system | 4.54 |
|  | *XC_0435* |  | cation efflux system protein | 3.61 |
|  | *XC_0558* | *pbuA* | ferric pseudobactin M114 receptor protein | 5.45 |
|  | *XC_0642* | *fpvA* | ferripyoverdine receptor | 2.15 |
|  | *XC_0762* | *betT* | high-affinity choline transport | 6.25 |
|  | *XC_0772* | *brf* | putative malic acid transport protein | 3.29 |
|  | *XC_0810* | *citM* | ferrous iron transport protein | 2.44 |
|  | *XC_0915* |  | TonB-like protein | 2.31 |
|  | *XC_0919* | *cirA* | TonB-dependent receptor | 2.42 |
|  | *XC_1104* | *iucA* | iron transporter | 3.53 |
|  | *XC_1108* | *fecA* | citrate-dependent iron transporter | 3.39 |
|  | *XC_1115* | *bfeA* | ferric enterobactin receptor | 2.35 |
|  | *XC_1222* | *iroN* | TonB-dependent receptor | 2.56 |
|  | *XC_1332* | *comEA* | DNA transport competence protein | 6.77 |
|  | *XC_1341* | *fhuA* | TonB-dependent receptor | 5.47 |
|  | *XC_1537* | *yxaH* | transport protein | 2.99 |
|  | *XC_1882* | *cynX* | MFS transporter | 5.38 |
|  | *XC_1947* | *smf2* | manganese transport protein | 2.07 |
|  | *XC_2354* | *feoB* | ferrous iron transport protein B | 2.37 |
|  | *XC_2355* | *feoA* | Mg++ transporter | 4.9 |
|  | *XC_2460* | *sglT* | sodium/glucose cotransport protein | 3.06 |
|  | *XC_2484* |  | TonB-dependent receptor | 9.73 |
|  | *XC_2485* | *fhuA* | TonB-dependent receptor | 5.22 |
|  | *XC_2546* |  | MFS transporter | 2.06 |
|  | *XC_2547* |  | ABC transporter ATP-binding protein | 3.5 |
|  | *XC_2569* | *ccmC* | heme exporter protein C/cytochrome C-type biogenesis protein | 3.09 |
|  | *XC_2690* |  | ABC transporter ATP-binding protein | 3.81 |
|  | *XC_2703* | *ychM* | sulfate transporter | 6.26 |
|  | *XC_2710* | *pstA* | ABC transporter phosphate permease | 2.71 |
|  | *XC_2846* | *fhuA* | iron receptor | 5.7 |
|  | *XC_2907* | *pstC* | ABC transporter phosphate permease | 3.37 |
|  | *XC_2923* | *araJ* | MFS transporter | 4.01 |
|  | *XC_2984* | *btuB* | TonB-dependent receptor | 7.28 |
|  | *XC_3205* | *fecA* | TonB-dependent receptor | 4.18 |
|  | *XC_3289* | *fecA* | TonB-dependent receptor | 2.63 |
|  | *XC_3293* | *cysW* | ABC transporter sulfate permease | 3.15 |
|  | *XC_3294* | *cysU* | ABC transporter sulfate permease | 2.8 |
|  | *XC_3531* | *kdpB* | potassium-transporting ATPase B chain | 2.09 |
|  | *XC_4044* | *piuB* | iron-uptake factor | 3.57 |
|  | *XC_4079* | *mgtE* | ABC transporter ATP-binding subunit | 4.78 |
|  | *XC_4141* | *fecA* | TonB-dependent receptor | 2.11 |
|  | *XC_4247* | *nodT* | outer membrane efflux protein | 2.15 |
|  | *XC_4249* |  | ferrichrome-iron receptor 3 | 2.04 |
|  | *XC_4316* | *ttuB* | MFS transporter | 2.94 |
|  | *XC_0371* | *glpF* | glycerol uptake facilitator protein | -3.76 |
|  | *XC_0405* | *fhuA* | TonB-dependent receptor | -4.76 |
|  | *XC_0409* | *bfeA* | TonB-dependent receptor truncated and shifted | -1.9 |
|  | *XC_0417* |  | ABC transporter substrate binding protein | -4.31 |
|  | *XC_0494* | *bfr* | bacterioferritin | -2.3 |
|  | *XC_0759* | *btuB* | TonB-dependent receptor | -1.77 |
|  | *XC_1004* | *iroN* | TonB-dependent receptor | -3.17 |
|  | *XC_1009* | *mscL* | large-conductance mechanosensitive channel | -2.39 |
|  | *XC_1165* | *fepA* | TonB-dependent receptor | -5.65 |
|  | *XC_1284* | *fecA* | TonB-dependent receptor | -2.44 |
|  | *XC_1451* | *fhuA* | TonB-dependent receptor | -3 |
|  | *XC_1887* | *cydC* | ABC transporter ATP-binding protein | -2.5 |
|  | *XC_2194* | *cirA* | TonB-dependent receptor | -6.59 |
|  | *XC_3201* |  | bacterioferritin | -6.76 |
|  | *XC_4146* | *ppa* | solute:Na+ symporter | -2.63 |
| Translation | *XC_0253* |  | dipeptidyl anminopeptidase | 2.73 |
|  | *XC_0602* |  | aminopeptidase N | 2.84 |
|  | *XC_0667* | *hslV* | ATP-dependent HslUV protease peptidase subunit HslV | 2.51 |
|  | *XC_0804* | *nonF* | NonF-related protein | 2.23 |
|  | *XC_0997* | *pepN* | aminopeptidase N | 2.5 |
|  | *XC_0998* |  | acetyltransferase | 2.31 |
|  | *XC_1296* |  | proline imino-peptidase | 2.03 |
|  |  |  |  |  |
|  | *XC_1715* |  | peptidase | 2.4 |
|  | *XC_2826* | *dcp* | peptidyl-dipeptidase | 2.07 |
|  | *XC_2862* |  | pili assembly chaperone | 5.15 |
|  | *XC_3041* | *trmD* | tRNA (guanine-N1-)-methyltransferase | 3.07 |
|  | *XC_3093* | *rplU* | 50S ribosomal protein L21 | 2.13 |
|  | *XC_3192* | *pepN* | aminopeptidase N | 2.19 |
|  | *XC_3280* |  | peptidyl-Asp metalloendopeptidase | 2.41 |
|  | *XC_3315* | *rplQ* | 50S ribosomal protein L17 | 2.12 |
|  | *XC_3322* | *rpmD* | 50S ribosomal protein L30 | 2.46 |
|  | *XC_3349* | *rplJ* | 50S ribosomal protein L10 | 2.09 |
|  | *XC_3550* |  | serine protease | 4.44 |
|  | *XC_3575* |  | serine protease | 4.99 |
|  | *XC_4122* | *rpmG* | 50S ribosomal protein L33 | 2.69 |
|  | *XC_4123* | *rpmB* | 50S ribosomal protein L28 | 2.53 |
|  | *XC_4144* |  | peptidyl-prolyl cis-trans isomerase | 2.26 |
| Transcription | *XC_0556* | *fecI* | RNA polymerase sigma factor | 3.1 |
|  | *XC_1836* |  | ribonuclease | 2.82 |
|  | *XC_2934* | *algU* | RNA polymerase sigma factor | 4.9 |
|  | *XC_1310* |  | sigma-54 modulation protein | -2.46 |
| Signal transduction | *XC_0420* |  | GGDEF family protein | 3.31 |
|  | *XC_0637* |  | GGDEF family protein | 2.52 |
|  | *XC_0641* |  | GGDEF family protein | 3.21 |
|  | *XC_0769* |  | two-component system sensor protein | 2.28 |
|  | *XC_1050* | *colS* | two-component system sensor protein | 3.24 |
|  | *XC_1476* |  | GGDEF family protein | 5.92 |
|  | *XC_2946* |  | two-component system sensor protein | 2.33 |
|  | *XC_3404* |  | two-component system regulatory protein | 2.22 |
|  | *XC_3669* | *ntrC* | response regulator hybrid protein | 3.02 |
|  | *XC_3998* |  | two-component system sensor protein | 2.15 |
|  | *XC_4030* | *phoQ* | two-component system sensor protein | 2.9 |
|  | *XC_0729* |  | two-component system regulatory protein | -2.45 |
|  | *XC_1150* | *exsF* | two-component system regulatory protein | -2.55 |
|  | *XC_1160* |  | two-component system response regulator | -2.14 |
|  | *XC_1755* |  | two-component system regulatory protein | -2.21 |
|  | *XC_2456* | *regS* | two-component system sensor protein | -2.26 |
|  | *XC_2457* | *regR* | two-component system regulatory protein | -3.63 |
|  | *XC_2578* |  | two-component system regulatory protein | -4.77 |
|  | *XC_3055* |  | two-component system regulatory protein | -1.86 |
|  | *XC_3060* |  | two-component system regulatory protein | -2.01 |
|  | *XC_3714* |  | two-component system sensor protein | -3.84 |
| Mobile genetic elements | *XC_0681* | *ISxac3* | ISxac3 transposase | 2.37 |
|  | *XC_0688* | *IS1480* | IS1480 transposase | 2.71 |
|  | *XC_2107* | *gII* | replication initiation protein | 2.05 |
|  | *XC_2111* | *gVIII* | major coat protein | 3040 |
|  | *XC_2120* |  | major coat protein | 12180 |
|  | *XC_2124* | *gII* | phage-related protein | 9.58 |
|  | *XC_2134* | *IS1480* | IS1480 transposase | 2.52 |
|  | *XC_2625* | *IS1404* | IS1404 transposase | 6.88 |
|  | *XC_2626* | *IS1404* | IS1404 transposase | 3.31 |
|  | *XC_3671* | *IS1404* | IS1404 transposase | 6.88 |
|  | *XC_3917* | *ISxcc1* | ISxcC1 transposase | 2.93 |
|  | *XC_3804* | *ISxac3* | ISxac3 transposase | -73.39 |
| Pathogenicity and adaptation | *XC_0026* | *egl* | cellulase | 3.28 |
|  | *XC_0056* |  | hemolysin III | 2.74 |
|  | *XC_0123* | *iroN* | TonB-dependent receptor | 3.04 |
|  | *XC_0125* |  | pectin methylesterase-like protein | 2.47 |
|  | *XC_0153* | *xynB* | xylanase | 2.95 |
|  | *XC_0241* |  | effector | 2.02 |
|  | *XC_0274* | *ohr* | organic hydroperoxide resistance protein | 2.23 |
|  | *XC_0532* | *cutA* | periplasmic divalent cation tolerance protein | 2.44 |
|  | *XC_0581* | *virP* | VirP protein | 2.75 |
|  | *XC_0705* | *peh-1* | endopolygalacturonase | 4.05 |
|  | *XC_0738* | *xcsC* | type II secretion system protein C | 3.36 |
|  | *XC_0739* | *xcsD* | type II secretion system protein D | 2.44 |
|  | *XC_0740* | *xcsE* | type II secretion system protein E | 2.3 |
|  | *XC_0744* | *xcsI* | type II secretion system protein I | 4.82 |
|  | *XC_0806* | *fyuA* | TonB-dependent receptor | 2.42 |
|  | *XC_1411* | *vieA* | response regulator | 3.53 |
|  | *XC_1432* | *mexA* | multidrug resistance protein | 2.74 |
|  | *XC_1433* | *mexB* | multidrug-efflux transporter | 2.43 |
|  | *XC_1447* |  | extracellular serine protease | 4.39 |
|  | *XC_1811* | *acvB* | virulence protein | 6.38 |
|  | *XC_1849* | *pglA* | polygalacturonase | 2.55 |
|  | *XC_2324* | *pdeA* | c-di-GMP phosphodiesterase A | 2.2 |
|  | *XC_2396* | *fhaB* | filamentous haemagglutinin | 3.89 |
|  | *XC_2483* |  | cellulase | 6.42 |
|  | *XC_2834* | *bglS* | beta-glucosidase | 2.9 |
|  | *XC_3019* | *hrpD5* | HrpD5 protein | 2.2 |
|  | *XC_3077* | *hrpG* | HrpG protein | 2.26 |
|  | *XC_3306* | *gst* | glutathione S-transferase | 2.42 |
|  | *XC_3376* |  | extracellular protease | 3.87 |
|  | *XC_3377* |  | extracellular protease | 6.45 |
|  | *XC_3657* | *copB* | copper resistance protein B precursor | 2.01 |
|  | *XC_3815* |  | O-antigen ligase | 2.26 |
|  | *XC_3859* | *mtrC* | membrane fusion protein precursor | 2.02 |
|  | *XC_3886* | *bglX* | beta-glucosidase | 2.22 |
|  | *XC_0141* |  | alpha-amylase | -6.06 |
|  | *XC_0407* | *sodM* | superoxidase dismutase | -3.65 |
|  | *XC_0639* | *engXCA* | cellulase | -2.15 |
|  | *XC_0672* |  | multidrug resistance efflux pump | -2.98 |
|  | *XC_1005* |  | 1,4-beta-cellobiosidase | -2.52 |
|  | *XC_1076* | *ostA* | trehalose-6-phosphate synthase | -2.37 |
|  | *XC_1298* | *pelB* | pectate lyase II | -3.17 |
|  | *XC_1660* | *gumD* | GumD protein | -2.54 |
|  | *XC_1661* | *gumE* | gumE protein | -2.06 |
|  | *XC_1664* | *gumH* | GumH protein | -2.03 |
|  | *XC_1665* | *gumI* | GumI protein | -2.34 |
|  | *XC_1667* | *gumK* | gumK protein | -2.1 |
|  | *XC_1668* | *gumL* | GumL protein | -3.41 |
|  | *XC_1669* | *gumM* | GumM protein | -2.0 |
|  | *XC_1775* | *oprN* | outer membrane protein OprN precursor | -2.84 |
|  | *XC_1851* |  | general stress protein | -4.8 |
|  | *XC_2160* | *yapH* | YapH protein | -22.03 |
|  | *XC_2191* | *cypC* | fatty acid alpha hydroxylase | -21.63 |
|  | *XC_2329* | *rpfA* | aconitase | -3.04 |
|  | *XC_3153* | *tcmJ* | tetracenomycin polyketide synthesis protein | -4.09 |
|  | *XC_3200* |  | peroxiredoxin | -3.22 |
|  | *XC_3591* | *pel* | pectate lyase | -3.38 |
|  | *XC_3754* |  | Mn-containing catalase | -19.24 |
|  | *XC_4014* | *ecnA* | entericidin A | -2.01 |
| Undefined category | *XC_0341* | *attT* | AttT protein | 18.04 |
|  | *XC_0600* |  | putative transmembrane protein | 2.55 |
|  | *XC_1198* | *W78* | sulfotransferase | 2.47 |
|  | *XC_1201* | *rebB* | RebB protein | 6.54 |
|  | *XC_2156* |  | nodulation related protein | 2.35 |
|  | *XC_2489* |  | regucalcin | 3.38 |
|  | *XC_3387* |  | hydrolase | 5.98 |
|  | *XC_3895* |  | disulphide-isomerase | 2.03 |
|  | *XC_0592* |  | ThiJ/PfpI family protein | -2.55 |
|  | *XC_1254* |  | hydrolase | -4.38 |
|  | *XC_2087* |  | tannase precursor | -3.66 |
|  | *XC_2169* |  | glucose-1-phosphate cytidylyltransferase | -11.58 |
|  | *XC_2170* |  | putative epimerase/dehydratase | -13.53 |
|  | *XC_2172* |  | strU protein | -12.53 |
|  | *XC_3158* | *visC* | hydroxylase large subunit | -9.98 |
|  | *XC_4219* |  | nuclear receptor binding factor related protein | -2.41 |
|  | *XC_4291* |  | microcystin dependent protein | -2.08 |
|  | *XC_4293* |  | microcystin dependent protein | -4.02 |
|  | *XC_4294* |  | acetyltransferase | -3.76 |
| conserved hypothetical protein | *XC_0090* |  | conserved hypothetical protein | 4.74 |
|  | *XC_0091* |  | conserved hypothetical protein | 3.32 |
|  | *XC_0242* |  | conserved hypothetical protein | 5.26 |
|  | *XC_0251* |  | conserved hypothetical protein | 7.34 |
|  | *XC_0258* |  | conserved hypothetical protein | 2.58 |
|  | *XC_0259* |  | conserved hypothetical protein | 4.65 |
|  | *XC_0260* |  | conserved hypothetical protein | 4.99 |
|  | *XC_0261* |  | conserved hypothetical protein | 4.45 |
|  | *XC_0262* |  | conserved hypothetical protein | 3.12 |
|  | *XC_0263* |  | conserved hypothetical protein | 2.97 |
|  | *XC_0337* |  | conserved hypothetical protein | 3.2 |
|  | *XC_0338* |  | conserved hypothetical protein | 2.72 |
|  | *XC_0339* |  | conserved hypothetical protein | 2.69 |
|  | *XC_0340* |  | conserved hypothetical protein | 9.02 |
|  | *XC_0359* |  | conserved hypothetical protein | 4.07 |
|  | *XC_0362* |  | conserved hypothetical protein | 3.72 |
|  | *XC_0364* |  | conserved hypothetical protein | 3.37 |
|  | *XC_0388* |  | conserved hypothetical protein | 2.06 |
|  | *XC_0603* |  | conserved hypothetical protein | 2.25 |
|  | *XC_0604* |  | conserved hypothetical protein | 3.48 |
|  | *XC_0605* |  | conserved hypothetical protein | 2.72 |
|  | *XC_0606* |  | conserved hypothetical protein | 2.65 |
|  | *XC_0608* |  | conserved hypothetical protein | 2.18 |
|  | *XC_0650* |  | conserved hypothetical protein | 2.04 |
|  | *XC_0715* |  | conserved hypothetical protein | 3.22 |
|  | *XC_1066* |  | conserved hypothetical protein | 2.18 |
|  | *XC_1081* |  | conserved hypothetical protein | 2.88 |
|  | *XC_1106* |  | conserved hypothetical protein | 4.2 |
|  | *XC_1107* |  | conserved hypothetical protein | 2.53 |
|  | *XC_1151* |  | conserved hypothetical protein | 3.74 |
|  | *XC_1194* |  | conserved hypothetical protein | 2.06 |
|  | *XC_1202* |  | conserved hypothetical protein | 4.67 |
|  | *XC_1208* |  | conserved hypothetical protein | 2.05 |
|  | *XC_1229* |  | conserved hypothetical protein | 3.06 |
|  | *XC_1336* |  | conserved hypothetical protein | 7.85 |
|  | *XC_1337* |  | conserved hypothetical protein | 8.46 |
|  | *XC_1338* |  | conserved hypothetical protein | 6.46 |
|  | *XC_1339* |  | conserved hypothetical protein | 6.5 |
|  | *XC_1340* |  | conserved hypothetical protein | 6.1 |
|  | *XC_1348* |  | conserved hypothetical protein | 3.86 |
|  | *XC_1485* |  | conserved hypothetical protein | 2.29 |
|  | *XC_1709* |  | conserved hypothetical protein | 4.05 |
|  | *XC_1710* |  | conserved hypothetical protein | 4.06 |
|  | *XC_1740* |  | conserved hypothetical protein | 2.33 |
|  | *XC_2024* |  | conserved hypothetical protein | 2.12 |
|  | *XC_2025* |  | conserved hypothetical protein | 4.81 |
|  | *XC_2027* |  | conserved hypothetical protein | 2.37 |
|  | *XC_2032* |  | conserved hypothetical protein | 3.37 |
|  | *XC_2037* |  | conserved hypothetical protein | 2.25 |
|  | *XC_2045* |  | conserved hypothetical protein | 7.86 |
|  | *XC_2047* |  | conserved hypothetical protein | 3.36 |
|  | *XC_2224* |  | conserved hypothetical protein | 4.48 |
|  | *XC_2230* |  | conserved hypothetical protein | 2.3 |
|  | *XC_2301* |  | conserved hypothetical protein | 3.89 |
|  | *XC_2305* |  | conserved hypothetical protein | 3.39 |
|  | *XC_2317* |  | conserved hypothetical protein | 5.47 |
|  | *XC_2319* |  | conserved hypothetical protein | 3.52 |
|  | *XC_2361* |  | conserved hypothetical protein | 2.75 |
|  | *XC_2436* |  | hypothetical protein | 2.36 |
|  | *XC_2471* |  | conserved hypothetical protein | 2.36 |
|  | *XC_2479* |  | conserved hypothetical protein | 2.77 |
|  | *XC_2481* |  | conserved hypothetical protein | 3.99 |
|  | *XC_2539* |  | conserved hypothetical protein | 2.03 |
|  | *XC_2550* |  | conserved hypothetical protein | 2.62 |
|  | *XC_2554* |  | conserved hypothetical protein | 3.75 |
|  | *XC_2705* |  | hypothetical protein | 2.08 |
|  | *XC_2786* |  | conserved hypothetical protein | 3.74 |
|  | *XC_2787* |  | conserved hypothetical protein | 5.73 |
|  | *XC_2788* |  | conserved hypothetical protein | 3.93 |
|  | *XC_2789* |  | conserved hypothetical protein | 3.51 |
|  | *XC_3080* |  | conserved hypothetical protein | 2 |
|  | *XC_3100* |  | conserved hypothetical protein | 3.76 |
|  | *XC_3145* |  | conserved hypothetical protein | 2.61 |
|  | *XC_3149* |  | conserved hypothetical protein | 3.09 |
|  | *XC_3150* |  | conserved hypothetical protein | 2.96 |
|  | *XC_3367* |  | conserved hypothetical protein | 3.05 |
|  | *XC_3477* |  | conserved hypothetical protein | 2.41 |
|  | *XC_3540* |  | conserved hypothetical protein | 3.15 |
|  | *XC_3782* |  | conserved hypothetical protein | 3.08 |
|  | *XC_3783* |  | conserved hypothetical protein | 4.85 |
|  | *XC_3786* |  | conserved hypothetical protein | 2.06 |
|  | *XC_3812* |  | conserved hypothetical protein | 2.53 |
|  | *XC_3842* |  | conserved hypothetical protein | 2.55 |
|  | *XC_4027* |  | conserved hypothetical protein | 2.35 |
|  | *XC_4028* |  | conserved hypothetical protein | 3.78 |
|  | *XC_4029* |  | conserved hypothetical protein | 3.8 |
|  | *XC_4034* |  | conserved hypothetical protein | 2.28 |
|  | *XC_4035* |  | conserved hypothetical protein | 4.92 |
|  | *XC_4084* |  | conserved hypothetical protein | 2.8 |
|  | *XC_0025* |  | conserved hypothetical protein | -5.42 |
|  | *XC_0034* |  | conserved hypothetical protein | -4.11 |
|  | *XC_0105* |  | conserved hypothetical protein | -2.55 |
|  | *XC_0108* |  | conserved hypothetical protein | -5.51 |
|  | *XC_0117* |  | conserved hypothetical protein | -7.85 |
|  | *XC_0176* |  | conserved hypothetical protein | -3.15 |
|  | *XC_0177* |  | conserved hypothetical protein | -3.83 |
|  | *XC_0278* |  | conserved hypothetical protein | -2.56 |
|  | *XC_0284* |  | conserved hypothetical protein | -2.48 |
|  | *XC_0717* |  | conserved hypothetical protein | -2.15 |
|  | *XC_0723* |  | conserved hypothetical protein | -2.36 |
|  | *XC_0727* |  | conserved hypothetical protein | -9.99 |
|  | *XC_1077* |  | conserved hypothetical protein | -2.11 |
|  | *XC_1388* |  | conserved hypothetical protein | -2.86 |
|  | *XC_1487* |  | conserved hypothetical protein | -3.07 |
|  | *XC_1493* |  | conserved hypothetical protein | -3.79 |
|  | *XC_2152* |  | conserved hypothetical protein | -6.33 |
|  | *XC_2161* |  | conserved hypothetical protein | -2.26 |
|  | *XC_2166* |  | conserved hypothetical protein | -3.03 |
|  | *XC_2167* |  | conserved hypothetical protein | -2.57 |
|  | *XC_2168* |  | conserved hypothetical protein | -5.98 |
|  | *XC_2185* |  | conserved hypothetical protein | -4.05 |
|  | *XC_2187* |  | conserved hypothetical protein | -5.73 |
|  | *XC_2193* |  | conserved hypothetical protein | -5.26 |
|  | *XC_2730* |  | conserved hypothetical protein | -2.28 |
|  | *XC_2805* |  | conserved hypothetical protein | -4.58 |
|  | *XC_2828* |  | conserved hypothetical protein | -2.82 |
|  | *XC_2829* |  | conserved hypothetical protein | -2.68 |
|  | *XC_3152* |  | conserved hypothetical protein | -6.16 |
|  | *XC_3164* |  | conserved hypothetical protein | -3.46 |
|  | *XC_3168* |  | conserved hypothetical protein | -2.72 |
|  | *XC_3171* |  | conserved hypothetical protein | -6.94 |
|  | *XC_3173* |  | conserved hypothetical protein | -2.34 |
|  | *XC_3174* |  | conserved hypothetical protein | -2.89 |
|  | *XC_3549* |  | conserved hypothetical protein | -3.35 |
|  | *XC_3711* |  | conserved hypothetical protein | -2.52 |
|  | *XC_3744* |  | conserved hypothetical protein | -2.47 |
|  | *XC_3752* |  | conserved hypothetical protein | -9.85 |
|  | *XC_3753* |  | conserved hypothetical protein | -41.95 |
|  | *XC_3755* |  | conserved hypothetical protein | -11.59 |
|  | *XC_3756* |  | conserved hypothetical protein | -7.53 |
|  | *XC_3764* |  | conserved hypothetical protein | -2.8 |
|  | *XC_3765* |  | conserved hypothetical protein | -4.73 |
|  | *XC_3768* |  | conserved hypothetical protein | -3.3 |
|  | *XC_3778* |  | conserved hypothetical protein | -4.16 |
|  | *XC_3779* |  | conserved hypothetical protein | -2.08 |
|  | *XC_3855* |  | conserved hypothetical protein | -5.44 |
|  | *XC_3856* |  | conserved hypothetical protein | -2.87 |
|  | *XC_3874* |  | conserved hypothetical protein | -2.26 |
|  | *XC_3882* |  | conserved hypothetical protein | -3.26 |
|  | *XC_3883* |  | conserved hypothetical protein | -4.41 |
|  | *XC_3971* |  | conserved hypothetical protein | -2.82 |
|  | *XC_3975* |  | conserved hypothetical protein | -2.64 |
|  | *XC_3976* |  | conserved hypothetical protein | -11.49 |
|  | *XC_3977* |  | conserved hypothetical protein | -8.47 |
|  | *XC_4147* |  | conserved hypothetical protein | -2.39 |
|  | *XC_4148* |  | conserved hypothetical protein | -3.03 |
|  | *XC_4284* |  | conserved hypothetical protein | -2.63 |
|  | *XC_4303* |  | conserved hypothetical protein | -2.01 |
|  | *XC_4307* |  | conserved hypothetical protein | -2 |
|  | *XC_4308* |  | conserved hypothetical protein | -2.04 |
|  | *XC_4310* |  | conserved hypothetical protein | -2.28 |
|  | *XC_4311* |  | conserved hypothetical protein | -2.35 |

Note: False discovery rate (FDR) ≤0.05 and absolute value of log_2_ fold change ≥1 were used as the cut off values. “+” represents gene up-regulated in the mutant ∆hpaS, and “-”represents gene down-regulated.
