# Supplementary material for: Xanthomonas campestris sensor kinase HpaS co‐opts the orphan response regulator VemR to form a branched two‐component system that regulates motility
Source: Mol Plant Pathol. 2020 Jan 9;21(3):360–75. doi: 10.1111/mpp.12901 (PMC7036368; doi:10.1111/mpp.12901)
Supplement: Supplementary file 7 [file MPP-21-360-s007.doc]

**Table S3. Confirmation of the gene expression profile data of the *hpaS* mutant by semi-quantitative RT-PCR.**

| ID | Gene | Annotation | Expresstion level | Semi RT-PCR wt/∆hpaS |
| --- | --- | --- | --- | --- |
| *XC_2377* | *hisH* | amidotransferase | 3.99↑ | 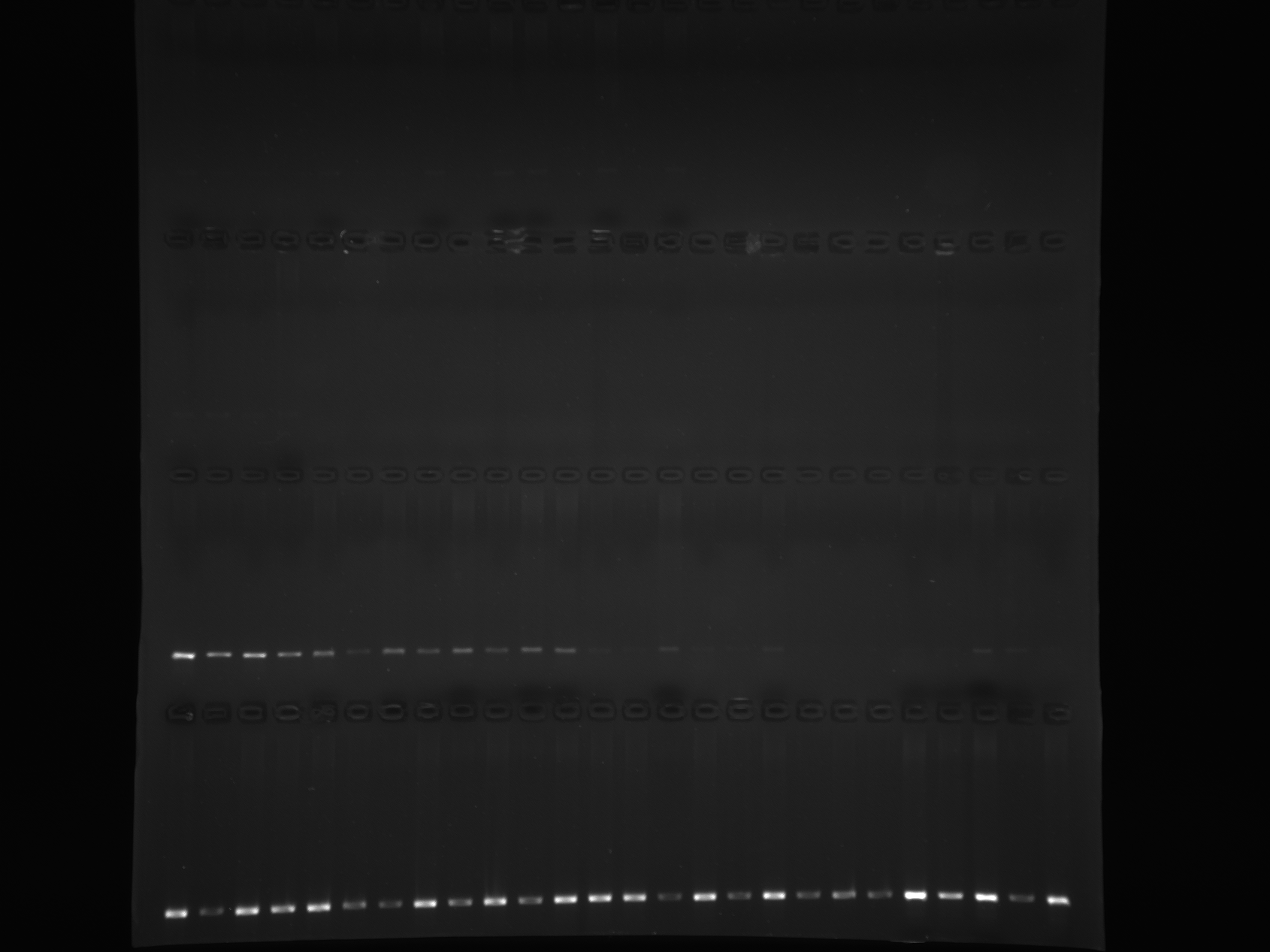 |
| *XC_0576* | *mdcE* | malonate decarboxylase | 2.33↑ | 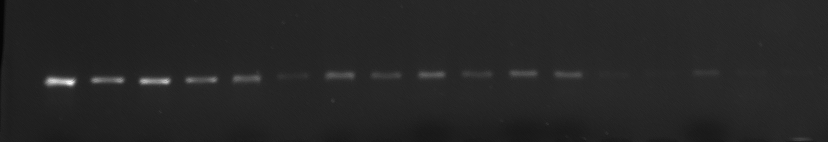 |
| *XC_1335* | *apbE* | lipoprotein ApbE precursor | 4.37↑ | 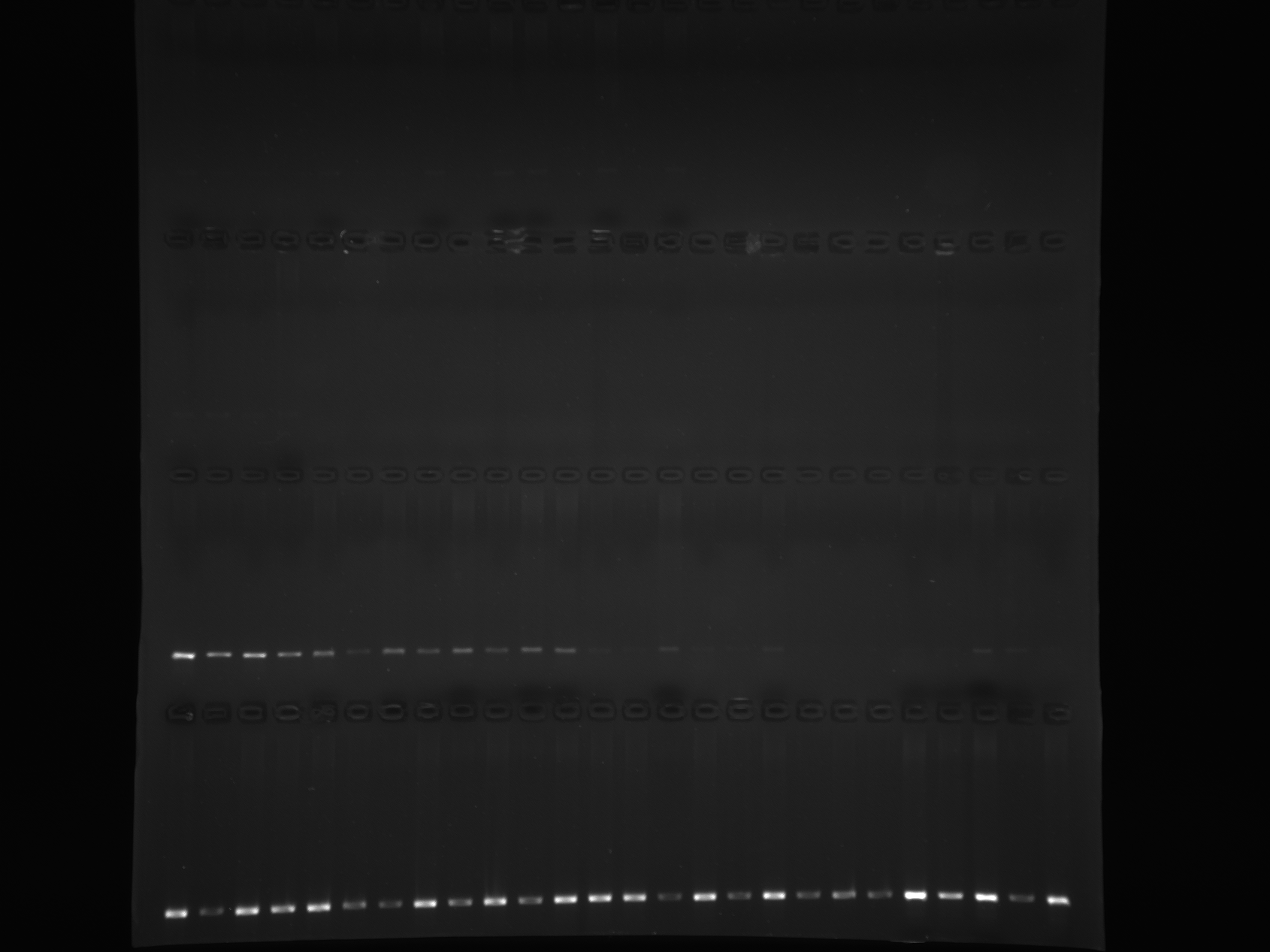 |
| *XC_1434* | *oprM* | outer membrane protein | 5.58↑ | 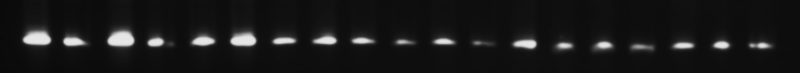 |
| *XC_1626* | *pilE1* | type IV pilin | -2.18↓ | 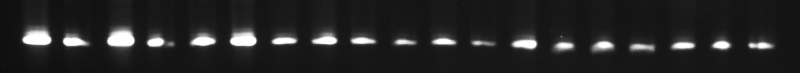 |
| *XC_2231* | *flgM* | flagellar protein | 2.22↑ | 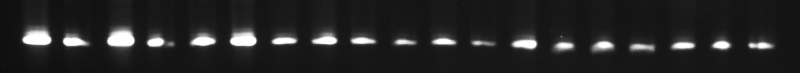 |
| *XC_2302* | *cheY* | chemotaxis response regulator | 3.3↑ | 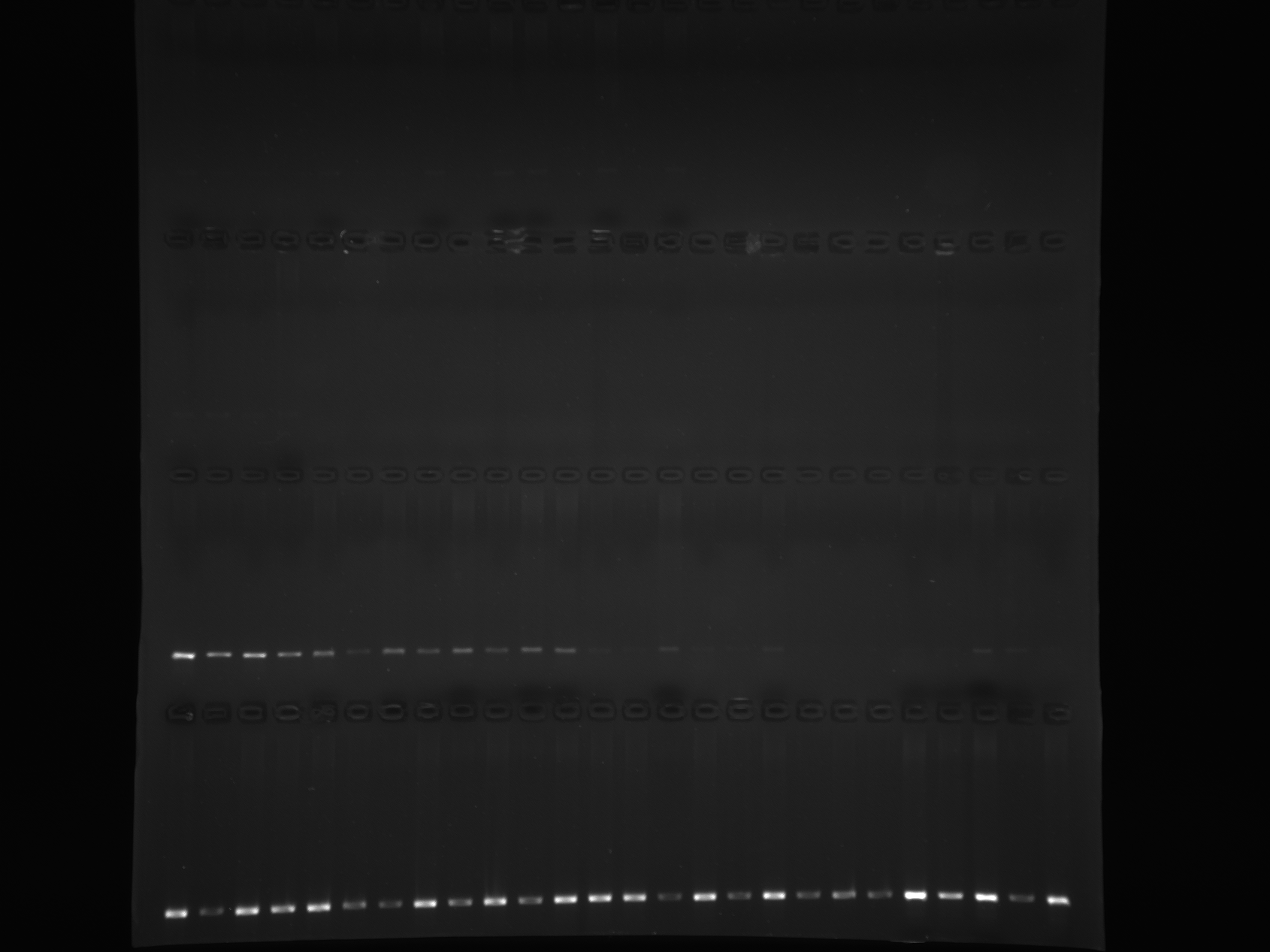 |
| *XC_1290* | *cheB* | protein-glutamate methylesterase (CheB) | -3.85↓ | 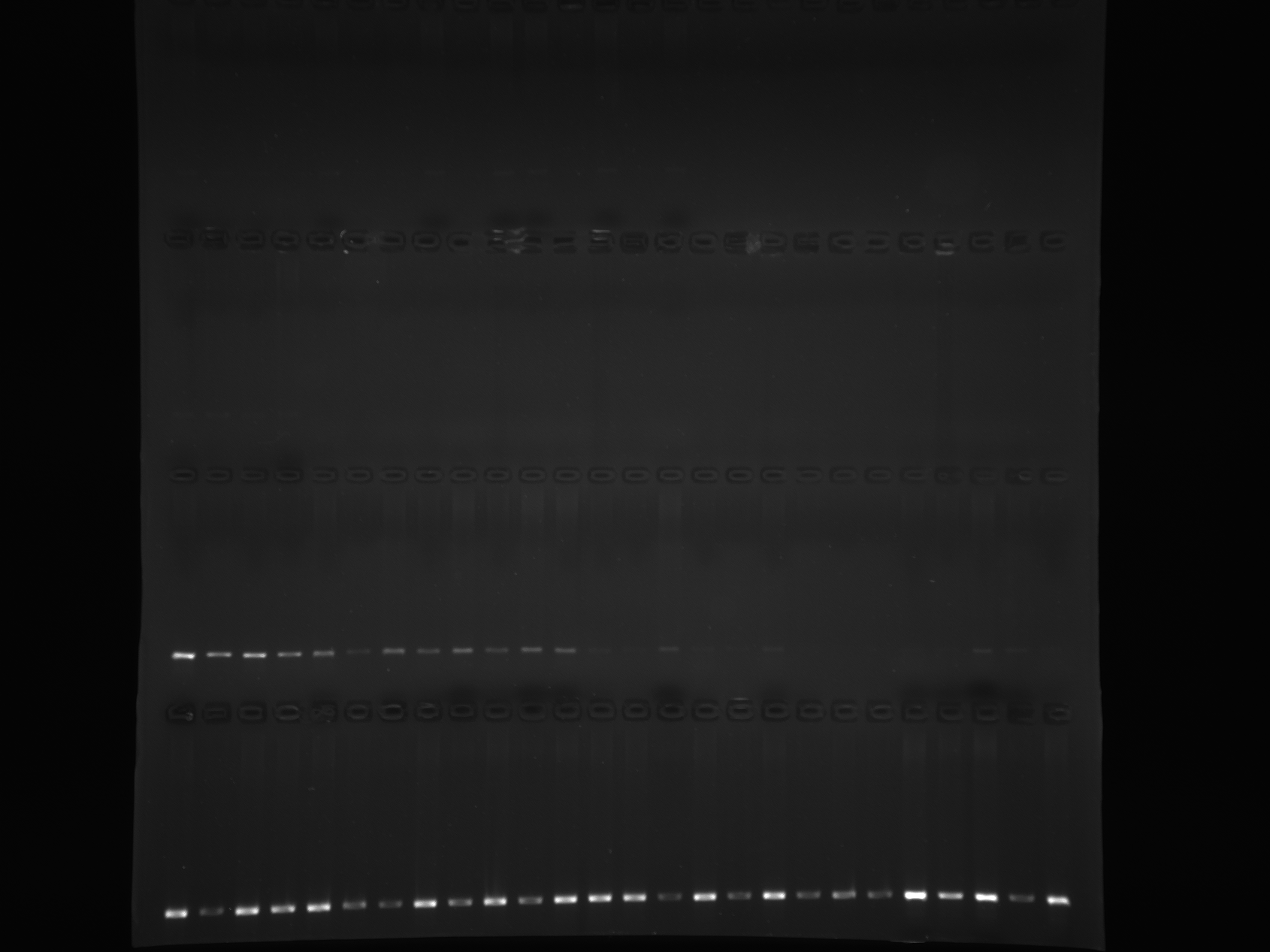 |
| *XC_2266* | *fliL* | flagellar biosynthesis protein | -2.24↓ | 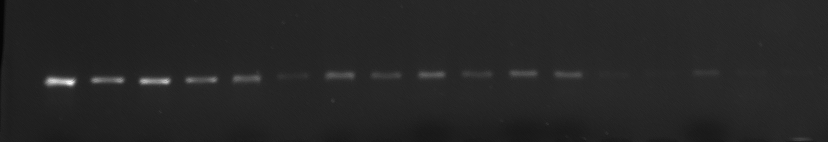 |
| *XC_0152* | *estA1* | carboxylesterase type B | 2.99↑ | 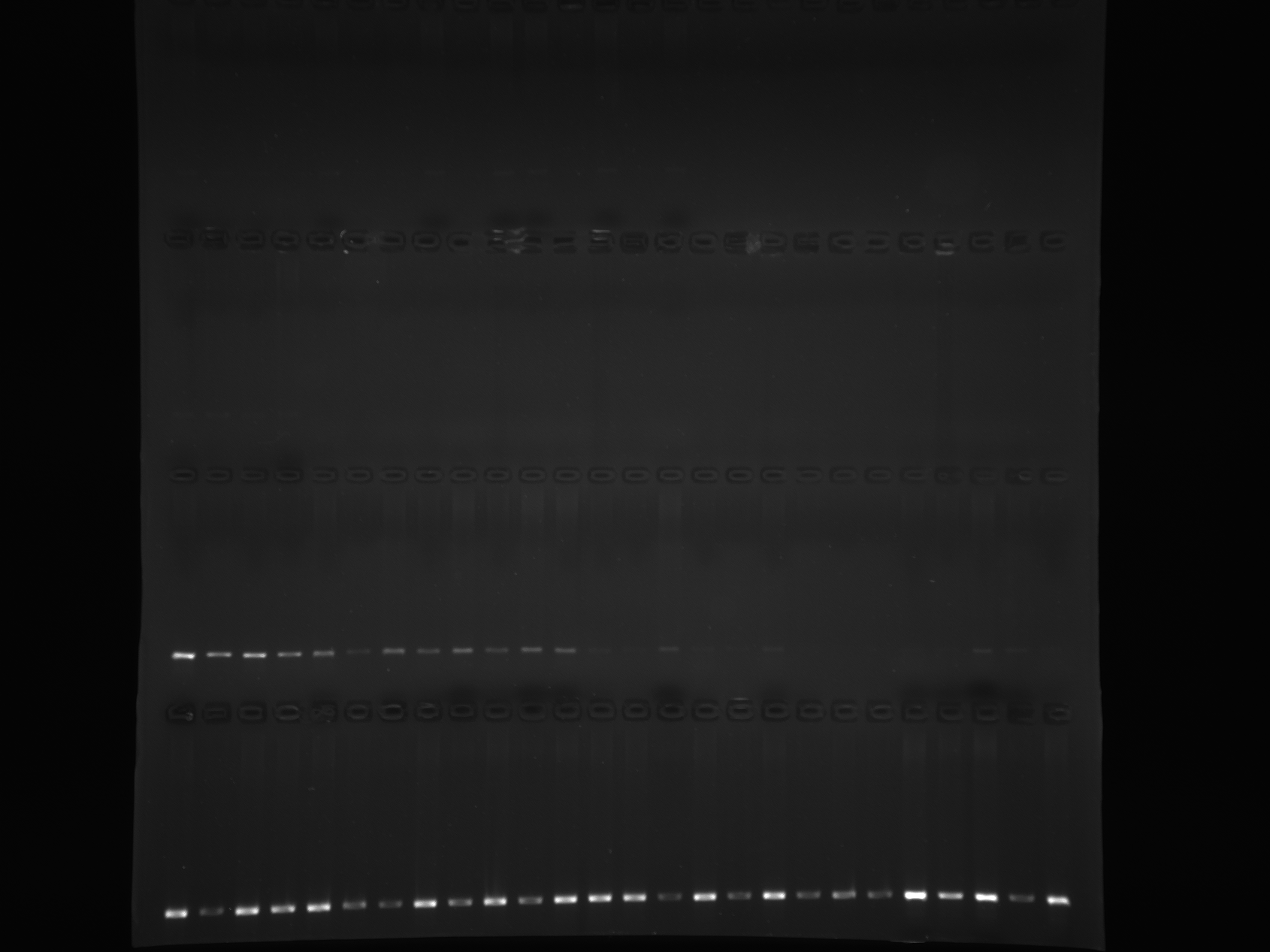 |
| *XC_0427* | *glgX* | glycogen debranching enzyme | -3.82↓ | 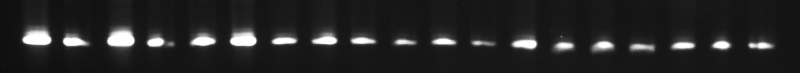 |
| *XC_3054* | *lamA* | endo-1,3-beta-glucanase precursor | -2.28↓ | 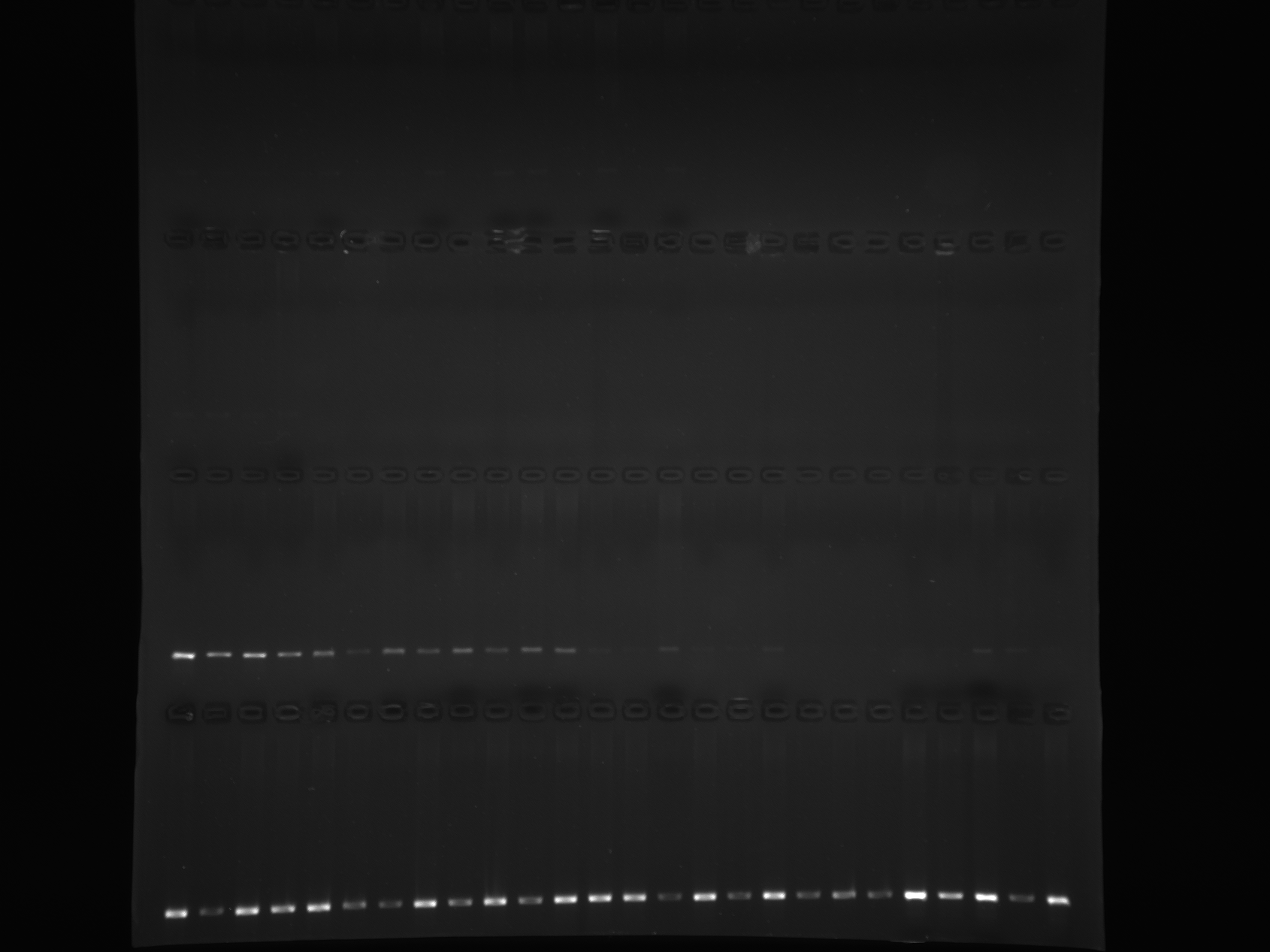 |
| *XC_1386* | *yagS* | oxidoreductase | -3.2↓ | 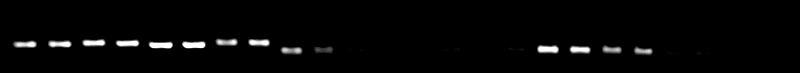 |
| *XC_3760* |  | transcriptional regulator NtrC family | -2.05↓ | 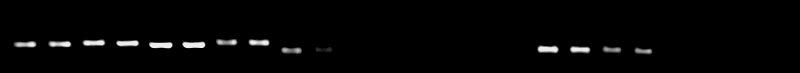 |
| *XC_0167* | *fpvA* | ferripyoverdine receptor | 3.87↑ | 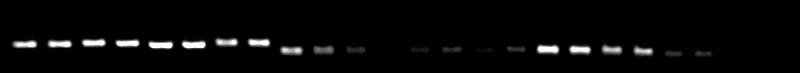 |
| *XC_1004* | *iroN* | TonB-dependent receptor | -3.17↓ | 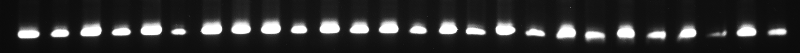 |
| *XC_1050* | *colS* | two-component system sensor protein | 3.24↑ | 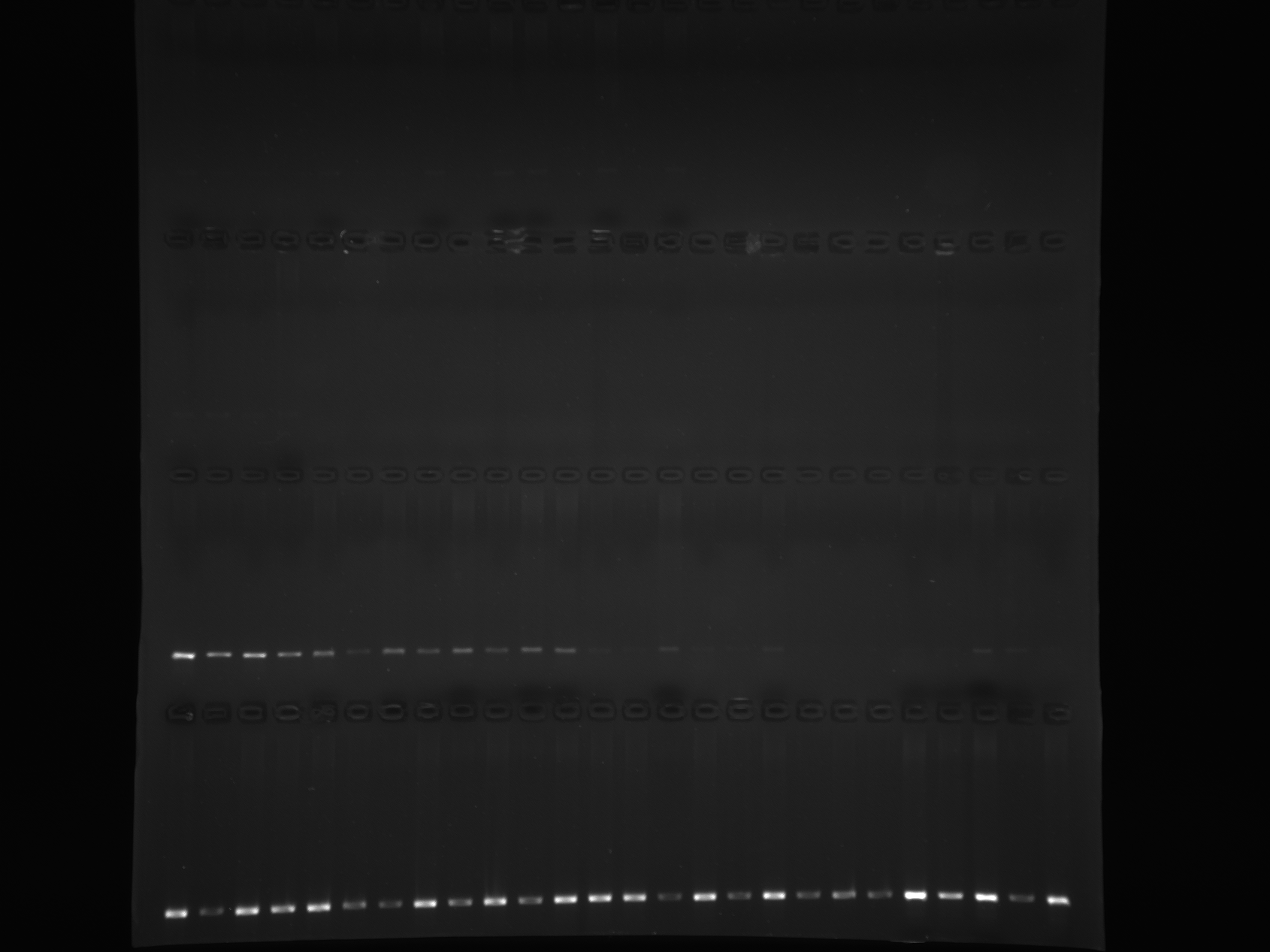 |
| *XC_3060* |  | two-component system regulatory protein | -2.01↓ | 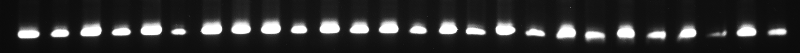 |
| *XC_0738* | *xcsC* | type II secretion system protein C | 3.36↑ | 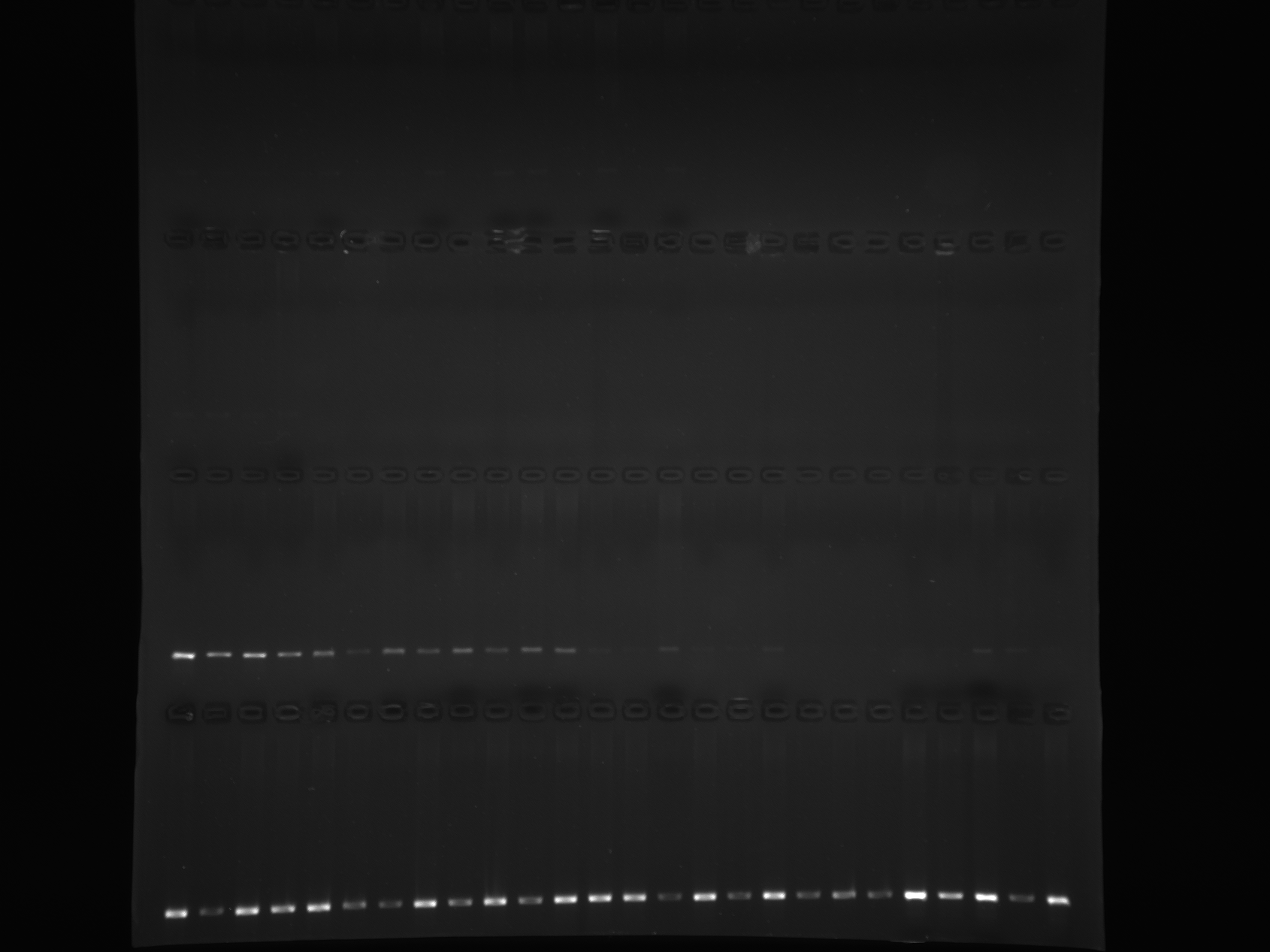 |
| *XC_0744* | *xcsI* | type II secretion system protein I | 4.82↑ | 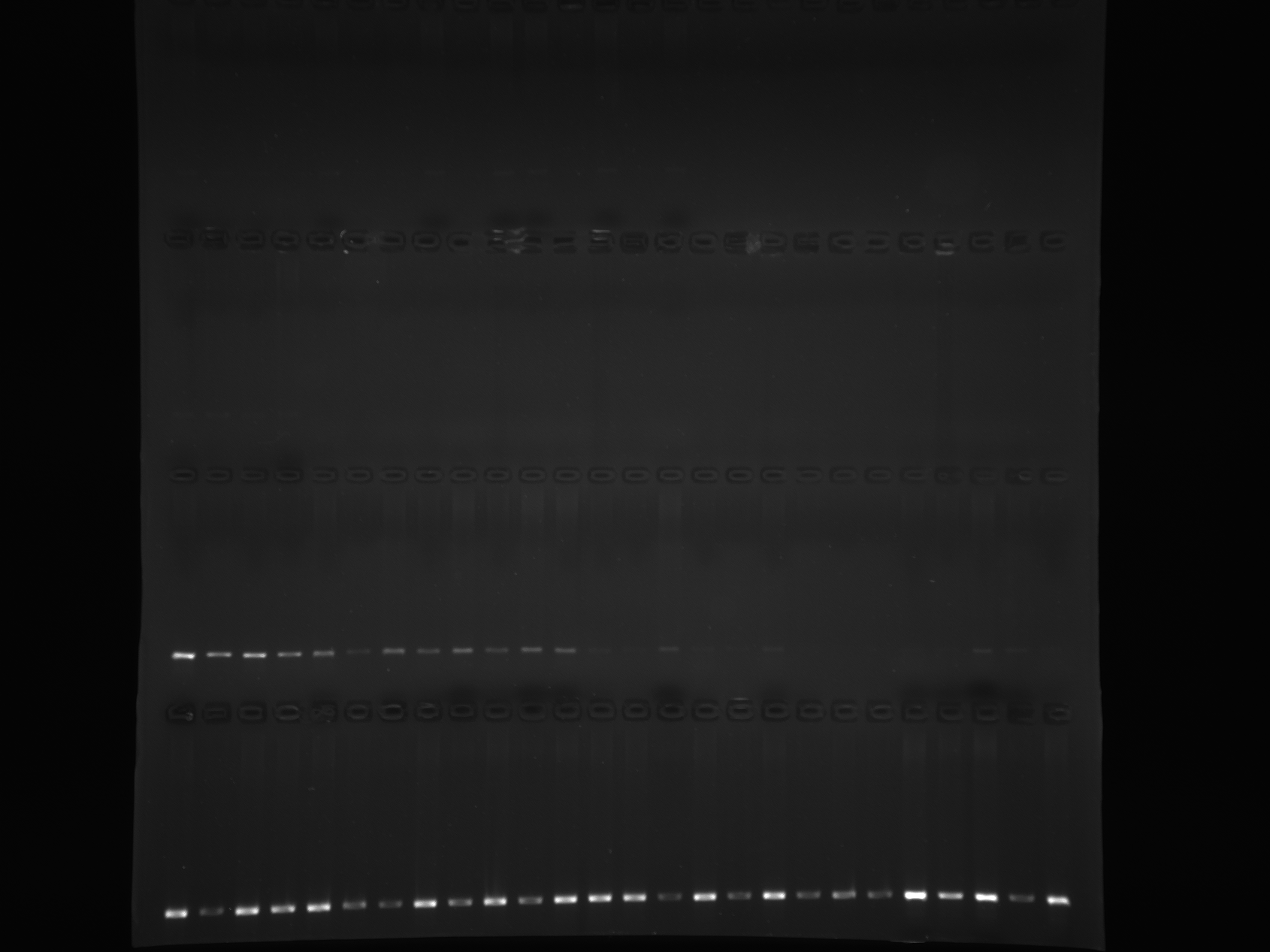 |
| *XC_3376* |  | extracellular protease | 3.87↑ | 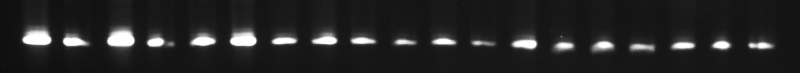 |
| *XC_0141* |  | alpha-amylase | -6.06↓ | 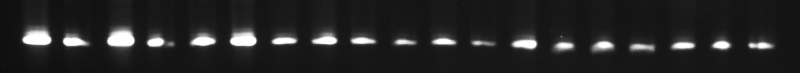 |
| *XC_4293* |  | microcystin dependent protein | -4.02↓ | 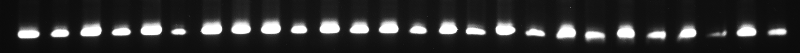 |
| *XC_0260* |  | conserved hypothetical protein | 4.99↑ | 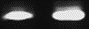 |
| *XC_2166* |  | conserved hypothetical protein | -3.03↓ | 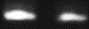 |
| *16S* |  |  |  | 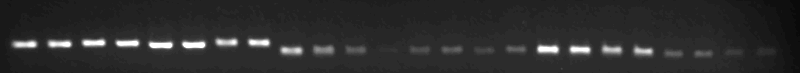 |

**Note:** RNA samples were processed using the same treatments as for RNA-Seq, and cDNA fragments were obtained by using a cDNA Synthesis kit (Invitrogen, Waltham, MA, USA). The 16S rRNA gene of *Xcc* 8004 was used as the internal control to verify the absence of significant variation at cDNA level in the samples. In this study, false discovery rate (FDR) ≤0.05 and absolute value of log_2_ fold change ≥1 were used as the cut off values. The acquired results were accordant to the transcriptome data.↑: up-regulated; ↓: down-regulated.
