# Supplementary material for: Xanthomonas campestris sensor kinase HpaS co‐opts the orphan response regulator VemR to form a branched two‐component system that regulates motility
Source: Mol Plant Pathol. 2020 Jan 9;21(3):360–75. doi: 10.1111/mpp.12901 (PMC7036368; doi:10.1111/mpp.12901)
Supplement: Supplementary file 8 [file MPP-21-360-s008.doc]

**Table S4. Primers used in this study^§^**

| Primer | Nucleotide sequence (5′→3′) | The amplified fragment or the utilization |
| --- | --- | --- |
| pR3VemR-F  pR3VemR-R | GGGGGATCCCGCCGCCGCACCACCAAAC  GGGAAGCTTTCACTCATTCCTGGCTCC | 434-bp DNA fragment of the *vemR* gene (including 50 nucleotides upstream of the start codon). Used for constitutively expression. |
| *vemR-*OF  *vemR-*OR | GGGGGATCC ATGAGCAAACTCACCGTG  GGGAAGCTT TCACTCATTCCTGGCTCC | 381-bp DNA fragment of the *vemR* gene coding sequence. Used for overexpression and pull-down assays. |
| L*vemR-*F  L*vemR-*R | GGGGAATTC CGCGACATGCTGCAGGAA  GGGTCTAGA GTTTGCTCATAGTAGTCC | 510-bp DNA sequence upstream of *vemR* (*XC_2252*), used for constructing the *vemR* deletion mutant. |
| R*vemR-*F  R*vemR-*R | GGGTCTAGA GAATGAGTGAGTCCCGCATTCTG  GGGCTGCAG ACCACTTCCTTGCCGGTG | 526-bp DNA sequence downstream of *vemR*, used for constructing the *vemR* deletion mutant. |
| SU-F  SU-R | ACAGTTGAATTCGGGCAACGTGCGCGAGTT  ACAGTTGGTACCAGCAGCACCACCACGGCC | 412-bp fragment upstream of *hpaS*. Used for constructing the *hpaS* deletion mutant. |
| SD-F  SD-R | ACAGTTTCTAGACAACGGCATGAACGAGGC  ACAGTTCTGCAGCTCATACCGATCACTGCC | 475-bp fragment downstream of *hpaS*. Used for constructing the *hpaS* deletion mutant. |
| GF  GR | GGGGGTACCAATTGACATAAGCCTGTTCGGTTCG  GGGTCTAGATGACAATTTACCGAACAACTCCGC | 826-bp fragment containing Gentamicin-resistant gene. Used for constructing the *hpaS* deletion mutant. |
| L*fliM-*F  L*fliM-*R | GGGGGATCC TGGTTCTTCCTCGGCCACAAG  GGGTCTAGAGGGGACGGTGCCTTACTGGGT | 426-bp DNA sequence upstream of *fliM* (*XC_2267*), used for constructing the *fliM* deletion mutant. |
| R*fliM-*F  R*fliM-*R | GGGTCTAGAATGATCAACTCCGACATCCTC  GGGAAGCTTGTCGTTGATCACCACCACTTC | 294-bp DNA sequence downstream of *fliM*, used for constructing the *fliM* deletion mutant. |
| D11-F  D11-R | GTGCTGCTGGTCGACGCCCACGAGG GCTTCATCAA  TTGATGAAGCCCTCGTGGGCGTCGA CCAGCAGCAC | Used for *vemR* site-directed mutagenesis, replacing Asp11 to Ala11. |
| D56-F  D56-R | CCAATGTCGTGCTGATGGCCCTGGCCATGCCCGAG  CTCGGGCATGGCCAGGGCCATCAGCACGACATTGG | Used for *vemR* site-directed mutagenesis, replacing Asp56 to Ala56. |
| *vemR-*HisF  *vemR-*HisR | GGGGGATCC ATGAGCAAACTCACCGTG  GGGAAGCTTTCAGTGATGGTGATGGTGATGCTCATTCCTGGCTCC | DNA fragment encoding VemR protein fused with 6×His-tag at the C-terminus of VemR. Used for western blot analysis. |
| *fliM-*OF  *fliM*-OR | CGCGGATCC ATGAGCGTCAGTGATCTG  CCCAAGCTT TCATTTGCTGGAGTCCTG | 1011-bp DNA fragment of the *fliM* gene coding region. Used for complemented test, overexpression and pull-down assays. |
| L*cheY*-F  L*cheY*-R | GGGAATTCCCGCCGTGCGCAAGATCG  GGTCTAGACGATCAGGATCCGCATGT | 495-bp DNA sequence upstream of *cheY* (*XC_2282*), used for constructing the *cheY* deletion mutant. |
| R*cheY*-F  R*cheY*-R | GGTCTAGAAGCTGGGCAAGGTGTTCG  GGAAGCTTTCTTGTCCATCAGCGCGC | 423-bp DNA sequence downstream of *cheY* (*XC_2282*), used for constructing the *cheY* deletion mutant. |
| O*cheY*-F  O*cheY*-R | GGGCTGCAGATGCGGATCCTGATCGTG  GGGAAGCTTTCAGGCGGTCGCCGCCAG | 378-bp DNA fragment of the *cheY* (*XC_2282*) gene coding region. Used for complemented test. |
| *cheY*-OF  *cheY*-OR | CCCGAGCTC ATGCGGATCCTGATCGTG  CCCAAGCTT TCAGGCGGTCGCCGCCAG | 378-bp DNA fragment of the *cheY* (*XC_2282*) gene coding region. Used for overexpression and pull-down assays. |
| *hpaS-*FlagF  *hpaS-*FlagR | GGGGAGCTC atgctgtcgcgctccttcac  GGGGGATCCCTAGACTACAAAGACCATGACGGTGATTATAAAGATCATGATATCGACTACAAAGATGACGACGATAAACTCAAAATTGGGGGG | DNA fragment encoding HpaS protein fused with 3×Flag-tag at the C-terminally of HpaS, used for constructing *Xcc* strain producing HpaS::3×Flag protein. |
| *fliM*-BTF  *fliM*-BTR | GGGGGATCCATGAGCGTCAGTGATCTGCTTTCC  GGGCTCGAGTCATTTGCTGGAGTCCTGGGAGG | 1011-bp DNA fragment of the *FliM* gene coding region. Used for bacterial two-hybrid assays. |
| *vemR*-TRGF  *vemR*-TRGR | ggggaattctgagcaaactcaccgtgc  gggctcgagtcactcattcctggctcc | 381-bp DNA fragment of the *vemR* gene coding region. Used for bacterial two-hybrid assays. |
| *cheY*-TRGF  *cheY*-TRGR | CCGGAATTCATGCGGATCCTGATCGTGG  CCGCTCGAGTCAGGCGGTCGCCGCCAG | 378-bp DNA fragment of the *cheY* gene coding region. Used for bacterial two-hybrid assays. |
| O*hpaS*F  O*hpaS*R | GGGGAATTCCGCAGCGCGCCACCGCGCC  GGGGGATCCCTACTCAAAATTGGGGGG | 1080-bp DNA fragment spanning nucleotides 163 (or 162) to 1242 bp of *hpaS* coding sequence. Used for overexpression, phosphorylation, bacterial two-hybrid and pull-down assay (for cloned into pBT, a based indicated by squire was added). |
| 16SF  16SR | GCCTAACACATGCAAGTCGAACGGC  AATATTCCCCACTGCTGCCTCCCG | 325-bp DNA fragment of the 16S rDNA sequence, used for RT-PCR. |
| 2234-F  2234-R | TCCTTCCTAGGCATCCACGG  TAGTGCTTGGCATCGGTGGC | 188-bp DNA fragment spans nucleotides 19 to 206 bp of the *XC_2234*, used for RT-PCR. |
| 2235-F  2235-R | CTCCCGATTTTCGATGTCGC  CGCGTTGGTGGTGGTGATC | 210-bp DNA fragment spans nucleotides 10 to 219 bp of the *XC_2235*, used for RT-PCR. |
| 2259-F  2259-R | GATGCTGCGCGCGGAAATC  TTTCGAACGCCTTGGCCAGC | 154-bp DNA fragment spans nucleotides 31 to 184 bp of the *XC_2259*, used for RT-PCR. |
| 2266-F  2266-R | GACGAGAAGAAGAAGGGCGG ACCAGCTGCACTTCGACCTG | 254-bp DNA fragment spans nucleotides 40 to 293 bp of the *XC_2266,* used for RT-PCR. |
| 1660-F  1660-R | CGTGGGTTGCTGAGTGAGC  ATGACGCAGACCGACCACC | 243-bp DNA fragment spans nucleotides 256 to 498 bp of the *XC_1660*, used for RT-PCR. |
| 1661-F  1661-R | ATCGCGATGATCTTCGGC  TGAACAGCTTGGGCAACG | 163-bp DNA fragment spans nucleotides 211 to 373 bp of the *XC_1661*, used for RT-PCR. |
| 0738-F  0738-R | CCGCTGTCTGTGTGTTGCTC  ATCGCCGGTACTCAGGTACG | 239-bp DNA fragment spans nucleotides 59 to 297 bp of the *XC_0738*, used for RT-PCR. |
| 0744-F  0744-R | CAGGGTTCTCGCTGCTGGAA  GCAGTGCTTCAATCGCCTGG | 162-bp DNA fragment spans nucleotides 26 to 187 bp of the *XC_0744*, used for RT-PCR. |
| 3376-F  3376-R | CGAGCCACCAGCGTTTCATC  TCAGGCTTTCCGAGTCGACC | 210-bp DNA fragment spans nucleotides 197 to 406 bp of the *XC_3376*, used for RT -PCR. |
| 3377-F  3377-R | AGGTGAATCTGTCCGGGCTG  TCTTGAGCACTTCGGAGCCC | 204-bp DNA fragment spans nucleotides 101 to 304 bp of the *XC_3377*, used for RT-PCR. |
| 2377-F  2377-R | ATGACCGATCTCGCGCTCA  TCAGCGGCACCTGCAATTG | 223-bp DNA fragment spans nucleotides 1 to 223 bp of the *XC_2377*, used for RT-PCR. |
| 2231-F  2231-R | CGAAGGCAATCTACCGACCG  AACGCTTCCTTCACCGCCTG | 219-bp DNA fragment spans nucleotides 15 to 233bp of the *XC_2231*, used for RT-PCR. |
| 2245-F  2245-R | CCAACAGTTCGAGCATGGCG  ATACCGTCGTTGGCGTTGCG | 157-bp DNA fragment spans nucleotides 59 to215 bp of the *XC_2245*, used for RT-PCR. |
| 2247-F  2247-R | TTCCAATCGTCAGTACGCCG  TGAGATGGCCGACGATTGC | 203-bp DNA fragment spans nucleotides 9 to 211 bp of the *XC_2247*, used for RT-PCR. |
| 0576-F  0576-R | CTGGTTTGCAGCGGGGTTTC  ACGCAAAGCTGCCGAAGACG | 275-bp DNA fragment spans nucleotides 87 to 361 bp of the *XC_0576*, used for RT-PCR. |
| 1335-F  1335-R | CCTGGAGCGTGAAAATGG  AAGCCCCACAGGTCTACCA | 268-bp DNA fragment spans nucleotides 11 to 278 bp of the *XC_1335*, used for RT-PCR. |
| 1434-F  1434-R | ATCCGAATGACGCTTGCC  TGGATCACCTGTTGCAGCG | 224-bp DNA fragment spans nucleotides 10 to 233 bp of the *XC_1434*, used for RT-PCR. |
| 1626-F  1626-R | GCGATCTTGTCTGCCATTGC  GGGTGGTTTGAAGAGCGAGC | 211-bp DNA fragment spans nucleotides 34 to 244 bp of the *XC_1626*, used for RT-PCR. |
| 2302-F  2302-R | GAGCGCACGTATCTTGGTGG  CAGCATGGGCGTGAACTTGT | 238-bp DNA fragment spans nucleotides 3 to 240 bp of the *XC_2302*, used for RT-PCR. |
| 0141-F  0141-R | TGGCAGCTGGTTCTTCGATG  GCAACGACTCCAGCTGATGG | 173-bp DNA fragment spans nucleotides 615 to 787 bp of the *XC_0141*, used for RT-PCR. |
| 1290-F  1290-R | ACAGCGCAGTGCGTACTTCG  TGCAGGCACCCTTCGGTATC | 245-bp DNA fragment spans nucleotides 14 to 258 bp of the *XC_1290*, used for RT-PCR. |
| 0152-F  0152-R | CGATGCCAGTGCGTACAGTG  TGATCACCACCACATCGCCG | 221-bp DNA fragment spans nucleotides 132 to 352 bp of the *XC_0152*, used for RT-PCR. |
| 0427-F  0427-R | TGCTGGACCCGTATGCCAAG  ACGTGCGCCTCGTAGATCAC | 220-bp DNA fragment spans nucleotides 302 to 501 bp of the *XC_0427* used for RT-PCR. |
| 3054-F  3054-R | ATCGACGGCAACACGTGGAC  TGTCACCAGGCCCTCGACCC | 212-bp DNA fragment spans nucleotides 124 to 335 bp of the *XC_3054*, used for RT-PCR. |
| 1386-F  1386-R | GCACCAACCTGCTGGATCTG  AAATTGCCCGCAGTGGTGG | 247-bp DNA fragment spans nucleotides 92 to 338 bp of the *XC_1386*, used for RT-PCR. |
| 3760-F  3760-R | GCGCCTATGGACCGTCTTTC  ACCACGATCTGCGGAGTGTG | 242-bp DNA fragment spans nucleotides 13 to 254 bp of the *XC_3760*, used for RT-PCR. |
| 0167-F  0167-R | CGAGTCGCTTTTCAGCATCG  TGACCGTGGCAGAGGGAATG | 248-bp DNA fragment spans nucleotides 270 to 517 bp of the *XC_0167*, used for RT-PCR. |
| 1004-F  1004-R | TTTCGGCTGAAGACATCGGC  TCGACGAAATCCGGCTGTGC | 226-bp DNA fragment spans nucleotides 212 to 437 bp of the *XC_1004* used for RT-PCR. |
| 1050-F  1050-R | CGCAGCCGCATCATTCTCTC  TGTGAATGCCATCGCGCAG | 280-bp DNA fragment spans nucleotides 64 to 343 bp of the *XC_1050*, used for RT-PCR. |
| 3060-F  3060-R | ACGAACCCGCCAAGATCCTG  TGAAACGCACGCATCGGGTC | 283-bp DNA fragment spans nucleotides 38 to 320 bp of the *XC_3060*, used for RT-PCR. |
| 4293-F  4293-R | TCGGCGAAATCCAGCTGTTC  AGCTATTGCCCAACTGCCGG | 225-bp DNA fragment spans nucleotides 17 to 241 bp of the *XC_4293* used for RT-PCR. |
| 0260-F  0260-R | AGCGTAGTGCTGTTGCTGGC  CAACCACAGCGACAACACCG | 249-bp DNA fragment spans nucleotides 4 to 252 bp of the *XC_0260*, used for RT-PCR. |
| 2166-F  2166-R | TCCTGATCCAGGTGCTGCTG  GATCCAGGTCATCGGCCATC | 213-bp DNA fragment spans nucleotides 5 to 217 bp of the *XC_2166*, used for RT-PCR. |

^§^The underlined sequences indicate the restriction sites for *Bam*HI, *Eco*RI, *Hin*dIII, *Kpn*I, *Pst*I, *Sac*I, *Xba*I and *Xho*I, respectively.
