# Supplementary material for: Xanthomonas campestris sensor kinase HpaS co‐opts the orphan response regulator VemR to form a branched two‐component system that regulates motility
Source: Mol Plant Pathol. 2020 Jan 9;21(3):360–75. doi: 10.1111/mpp.12901 (PMC7036368; doi:10.1111/mpp.12901)
Supplement: Supplementary file 9 [file MPP-21-360-s009.pdf]

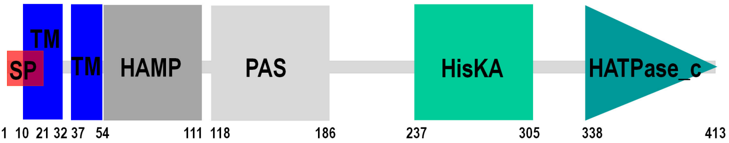

SP
TM
TM
HAMP
PAS
HisKA
HATPase_c
1
10
21
32
37
54
111
118
186
237
305
338
413

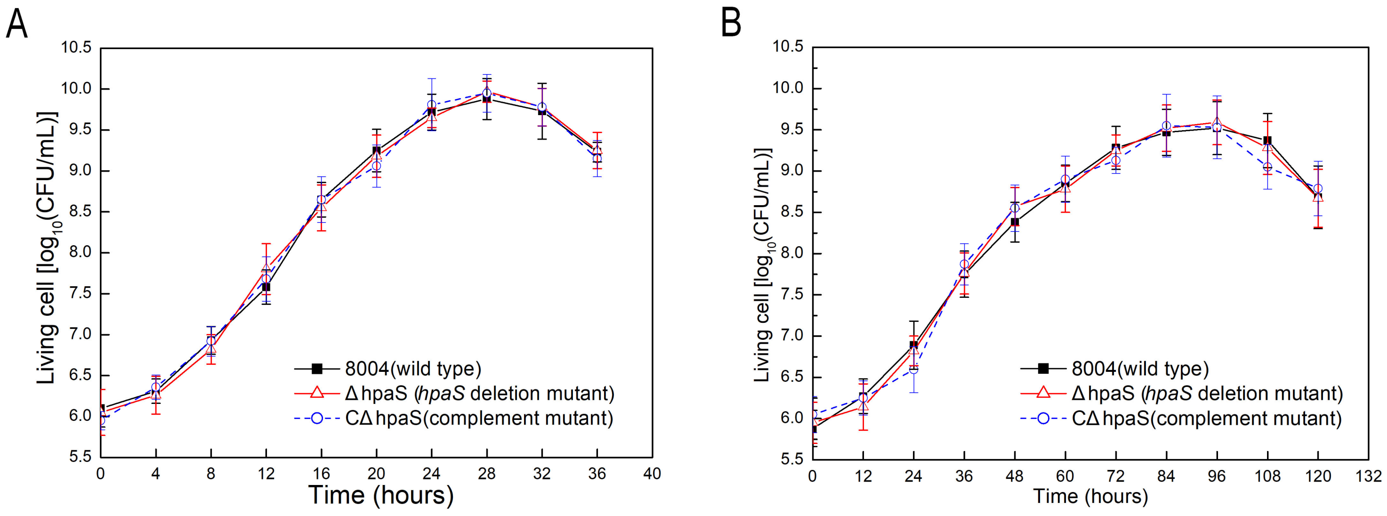

A
B
Living cell [log10(CFU/mL)]
Time (hours)
8004(wild type)
ΔhpaS (hpaS deletion mutant)
CΔhpaS(complement mutant)

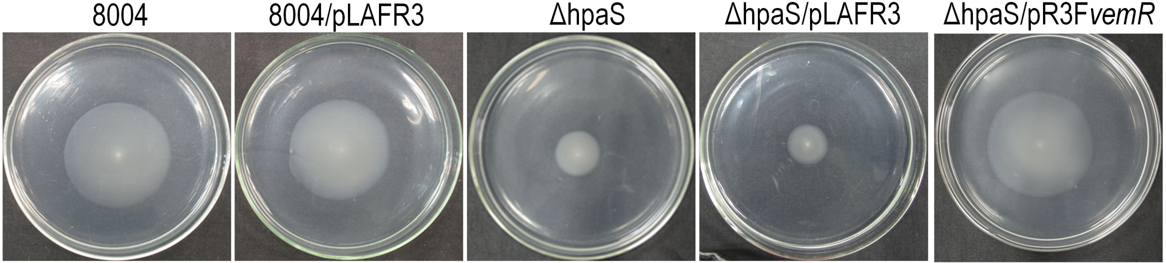
8004
8004/pLAFR3
ΔhpaS
ΔhpaS/pLAFR3
ΔhpaS/pR3F*vemR*

```
VemR   MSKLTVLLVDDHEGFINAAMRHFRKVEWLNIVGSAANGLEAIERSESLRP
CheY   ---MRILIVDDFSTMRRIVKNLLADLGFTNTA-EAEDGNSALAALRAGPF

VemR   NVVLMDLAMPEMGGLQATRLIKTQDDPPYIVIASHFDDAEHR--EHALRA
CheY   DFVVTDWNMPGMTGIDLLRNIRADAKLKHLPVMMVTAEAKREQIIEAAQC

VemR   GADNFVSKLSYIQEVMPILEGLTEGARNE-
CheY   GVNGYIIKPFTAQTLEEKLGKVFERLAATA
```
